# Identification and Expression Patterns of Three Vitellogenin Genes and Their Roles in Reproduction of the Alligatorweed Flea Beetle *Agasicles hygrophila* (Coleoptera: Chrysomelidae)

**DOI:** 10.3389/fphys.2019.00368

**Published:** 2019-04-02

**Authors:** Hong Zhang, Yao Wang, Yiran Liu, Meiting Zhao, Jisu Jin, Zhongshi Zhou, Jianying Guo

**Affiliations:** ^1^State Key Laboratory for Biology of Plant Diseases and Insect Pests, Institute of Plant Protection, Chinese Academy of Agricultural Sciences, Beijing, China; ^2^College of Agriculture, Ludong University, Yantai, China; ^3^College of Plant Protection, Hunan Agricultural University, Changsha, China

**Keywords:** *Agasicles hygrophila*, vitellogenin, RT-qPCR, RNAi, fecundity, ovary

## Abstract

The alligatorweed flea beetle *Agasicles hygrophila* is an insect used for biological control of the aquatic weed *Alternanthera philoxeroides* (alligatorweed). Because these insects are oviparous, synthesis, and transportation of yolk proteins is integral to reproduction. Vitellin, the chief protein constituent in egg yolk, is mainly synthesized in the fat body and its synthesis is regulated by the transcript levels of *Vitellogenin* (*Vg*). In our study, we first cloned and characterized three *Vg* genes from *A. hygrophila* and quantified the expression levels of these *Vgs* in different tissues and developmental stages by RT-qPCR. Analysis of the full-length cDNA sequences of the three *A. hygrophila Vg* genes revealed that the open reading frames of *AhVg1*, *AhVg2*, and *AhVg3* were 5175, 5346, and 5385 bp, encoding 1724, 1781, and 1794 amino acids, respectively. RT-qPCR analysis revealed that these three *AhVgs* have similar expression patterns; expression in the fat body was significantly higher than that in other tissues, and the highest expression was observed in the adult developmental stage. RNA interference was used to explore the functions of the *AhVgs*. *A. hygrophila* female adults injected with dsRNA targeting the *AhVg* genes showed decreased *AhVg* gene expression. Down regulation of all three *AhVgs* significantly affected ovary development, reduced egg laying capacity, and reduced the egg hatch rate compared with the control groups. Our findings provide the basis for further study of the functions of *Vg* genes in other insect species.

## Introduction

Vitellogenin (Vg), the precursor protein of egg yolk vitellin (Vn), provides the energy and material for the development of ovaries and embryos in insects (Sappington and Raikhel, 1998; Tufail and Takeda, 2008; Veerana et al., 2015). Vgs are mainly synthesized and processed in the fat body and then secreted into the hemolymph (Raikhel and Dhadialla, 1992) and transported into oocytes by the Vitellogenin receptor (VgR). Vgs are incorporated into oocytes and stored as Vns, a crystalline substance, and reserved for development of the ovary and future embryo (Sappington and Raikhel, 1998; Tufail and Takeda, 2008). In insects, Vg synthesis is regulated by the expression levels of *Vg* (Raikhel and Dhadialla, 1992), and previous research has shown that ecdysone, juvenile hormone, and neuropeptides each play a role in *Vg* gene regulation (Engelmann and Friedel, 1974; Wyatt and Davey, 1996).

The first insect Vg was discovered in the cecropia moth (*Hyalophora cecropia*) as a female-specific protein precursor for Vn or yolk protein (Pan et al., 1969). Insect Vgs were then shown to be multipart oligomeric glycolipophosphoproteins (Anderson, 1974). The molecular weights of insect Vgs vary in size from 200 to 250 kDa, with the large subunit ranging from 150 to 200 kDa and the small subunit from 40 to 65 kDa (Dhadialla and Raikhel, 1990; Kim et al., 2010). Comparison of insect Vg amino acid sequences revealed that they are highly conserved (Chen et al., 1997; Sappington and Raikhel, 1998). The similarity between Vgs is also evident based on their similar antigenicity (Tufail and Takeda, 2008). Vitellogenesis is important for fecundity and ovary development (Boldbaatar et al., 2010). For example, overexpression of Vg resulted in increased fecundity in *Tetranychus cinnabarinus* (Xing et al., 2016), and Vgs were shown to be crucial for ovary development in *Haemaphysalis longicornis* (Boldbaatar et al., 2010).

*Alternanthera philoxeroides* is commonly considered an invasive species and has spread to many countries (Julien et al., 1995; Zhang H. J. et al., 2016). The spread of *A. philoxeroides* has had a negative impact on the economy, environment, and society (Andres, 1977). *Agasicles hygrophila* (Selman & Vogt) (Chrysomelidae), the alligatorweed flea beetle, is a classical biological agent that has proven effective in controlling alligatorweed and is presently used in many countries (Napompeth, 1991; Buckingham, 1996). The life cycle of *A. hygrophila* can be completed in about 25 days in the appropriate habitat and temperature (∼22°C), and it can produce four generations per year (Zhao et al., 2016). *A. hygrophila* was introduced to the United States to control alligatorweed in the late 1960s (Zhao et al., 2016), and subsequently brought from Florida to China in 1987, where it was released in several provinces, including Jiangsu, Yunnan, Sichuan, and Hunan (Pan et al., 2006). Many studies have focused on the geographical distribution of *A. hygrophila* and its ability to control alligatorweed; however, studies of the reproductive physiology of *A. hygrophila* are rare. Knowledge of reproduction-related genes and proteins involved in ovary development and fecundity are necessary for better understanding the molecular mechanisms of reproduction in this species. Because *Vg* genes are known to be important reproductive genes regulating ovary development and fecundity in other insects, understanding the expression patterns and functions of these genes is essential for revealing the mechanism of reproduction in *A. hygrophila*.

In our study, we identified *Vgs* in *A. hygrophila*, characterizing *Vgs* at multiple levels with the use of gene cloning and sequence analysis. We established the full length *AhVg* cDNA sequences and compared the molecular and structural characteristics of these genes to *Vgs* from other insect species. We further analyzed the expression levels of *AhVgs* in different tissues and across developmental stages. Finally, we analyzed the role of the *AhVgs* in *A. hygrophila* ovary development and fecundity by utilizing RNA interference (RNAi).

## Materials and Methods

### Host Plants and Experimental Insects

Roots of *A. philoxeroides* were collected from standing water at the Institute of Plant Protection, Hunan Academy of Agricultural Sciences and planted in sterilized soil in plastic boxes (40 × 18 × 15 cm). Plants were grown in the greenhouse at Langfang Experimental Station, Chinese Academy of Agricultural Sciences (LF, CAAS), and watered every other day. *A. philoxeroides* were selected for experiments when they reached the four- to six-internodes stage.

*Agasicles hygrophila* adults were collected from a field in Changsha, Hunan province and were maintained on *A. philoxeroides* plants in the laboratory at the Chinese Academy of Agricultural Sciences (BJ, CAAS) under controlled conditions: 28 ± 2°C, 75 ± 5% relative humidity (RH), and a 12 h light:12 h dark regime. The insects were cultivated for three generations to eliminate maternal effects.

### Sample Collection

For cloning the *A. hygrophila Vg* genes, the abdomens of *A. hygrophila* females were dissected in 1× phosphate-buffered saline (PBS) under an Olympus stereomicroscope (SZX16, Olympus, Tokyo, Japan). For analysis of expression during different developmental stages, the freshly pupated pupae were collected daily, and female adults were collected every 2 days after emergence. For analysis of expression in different tissues, head, thorax, ovary, fat body, midgut, and wing tissues were dissected from 6-day-old females under an Olympus stereomicroscope (SZX16, Olympus, Tokyo, Japan) in PBS. All samples were frozen immediately in liquid nitrogen and subsequently kept at −80°C until further experimentation.

### RNA Isolation and Gene Cloning

TRIzol reagent (Life Technologies, Carlsbad, CA, United States) was used to extract total RNA from the above samples according to the manufacturer’s protocols. RNA integrity and concentration were assessed as described in An et al. (2016). The cDNA of *A. hygrophila Vg* genes was synthesized from total RNA isolated from the abdomen. We mined *A. hygrophila* transcriptome data to obtain three expressed sequence tags (ESTs) showing similarity to other insect Vg proteins. Gene-specific primers were designed based on these *A. hygrophila Vg* ESTs (Supplementary Table S1). Cloning was performed following the procedure from Ding et al. (2018). In brief, 5′ rapid amplification of cDNA ends (5′-RACE) and 3′ rapid amplification of cDNA ends (3′-RACE) to obtain the full length cDNA sequences of the *AhVgs* were done using the SMART RACE cDNA amplification kit (Clontech, Mountain View, CA, United States) following the manufacturer’s protocol. PCR amplification was carried out using the Phusion DNA Polymerase mix (New England BioLabs, Ipswich, MA, United States #M0530). The PCR conditions were as follows: 98°C for 30 s, followed by 35 cycles of 98°C for 10 s, 65°C for 10 s, and 72°C for 1 min, with a final 5 min extension. The RACE PCR products were analyzed by 1% agarose gel electrophoresis and purified using an AxyPrepTM DNA Gel Extraction Kit (Axygen, West Orange, NJ, United States). Finally, the purified distinct single-band PCR products were cloned into the pEASY-Blunt vector (Transgen, Beijing, China) and sequenced.

### Sequence Analysis and Phylogenetic Analysis

Open reading frames (ORFs) of the *AhVgs* were obtained using the NCBI ORF finder.^1^ The putative molecular weights, isoelectric points, and signal peptide positions were determined using the Signal IP 4.1 Server. For phylogenetic analysis, the Vg sequences from insects in Coleoptera, Hemiptera, Dictyoptera, Lepidoptera, Hymenoptera, and Diptera were downloaded from the NCBI reference sequences (RefSeq) database (Supplementary Table S2). *A. hygrophila* Vg sequences were aligned with these insect *Vgs* using ClustalW and MEGA 5.0 software (Tamura et al., 2011), following the methods of Zhang W. N. et al. (2016).

### Double-Stranded RNA Synthesis and RNAi

For each of the three *AhVgs*, we synthesized two double-stranded RNAs (dsRNAs) using gene-specific primers; the T7 promoter was added to the 5′-end of each primer (Supplementary Table S1). *EGFP* (GenBank accession number: AIR08541.1) dsRNA (ds*EGFP*) was synthesized as a negative control. dsRNA was synthesized using the HiScribe^TM^ T7 Quick High YieldRNA Synthesis Kit (New England BioLabs, Ipswich, MA, United States #E2050S) following the manufacturer’s protocol. The concentration of dsRNA was evaluated using a NanoVue spectrophotometer (GE-Healthcare, Germany), and the purity was verified by running the dsRNA on a 1.0% agarose gel (Supplementary Figure S1). In order to ensure that the volume of dsRNA injected into the control group and treatment group was the same, we adjusted the concentration of all synthesized dsRNA to 10,000 ng/μl (Supplementary Table S3).

The freshly emerged adult *A. hygrophila* females (<12 h following eclosion) were collected for dsRNA injection utilizing a PLI-100 Pico-Injector (Harvard Apparatus, Holliston, MA, United States) with an MP-255 Micromanipulator (Sutter, Novato, CA, United States) under an Olympus stereomicroscope. The dsRNA solution was injected into the conjunctivum on the abdomen of *A. hygrophila* female adults. The amount of dsRNA injected for different groups is shown in Supplementary Table S4. Each experiment was repeated at three times. Each repeat included 60 female individuals. Based on the results of the preliminary experiment, we injected about 80–90 females for our experiment to ensure the survival of 60 females for later experimental observation after injection. The female adults and freshly emerged wild adult males were kept in plastic bottles (8 × 10 cm) with fresh *A. philoxeroides* stems and a piece of moistened filter paper at the bottom to maintain a moist environment. Each pair of adults kept in a separate bottle (Guo et al., 2011). Of the 60 adult females, 20 were used for observation of oviposition, 30 were used for expression analysis of the three *Vg* genes, and 10 were used for observation of ovary development and measuring the lengths of the ovarioles. The plastic bottles with *A. hygrophila* adults were transferred to a climate chamber maintained at 28 ± 2°C, 75 ± 5% RH, and a 12 h light:12 h dark photoperiod (Guo et al., 2011).

### Expression Analysis of AhVgs

Total RNA was extracted with TRIzol reagent (Life Technologies, Carlsbad, CA, United States) following established protocols. qRT-PCR was conducted with the TransStart Green qPCR SuperMix Kit (Transgen, Beijing, China) on an ABI Prism 7500 instrument (Applied Biosystems, Carlsbad, CA, United States). We designed specific primer pairs for *AhVgs* with Beacon Designer 7.9 software (PREMIER Biosoft International, CA, United States) (Supplementary Table S1). qRT-PCR reactions and cycling were performed according to the instruction manual of the TransStart Tip Green qPCR SuperMix Kit (TransGen, Beijing, China). The qRT-PCR reactions were conducted in a 20 μl mixture containing 10 μl of 2 × TransStart Green qPCR SuperMix, 0.4 μl of each primer (10 μM), 0.4 μl of 50 × Rox Reference Dye, 200 ng of sample cDNA and 7.8 μl of ddH_2_O. The qRT-PCR cycling parameters were as follows: 94°C for 30 s, followed by 40 cycles of 94°C for 30 s, 55°C for 30 s, and 72°C for 10 s, with melt curve stages at 95°C for 15 s, 60°C for 1 min, and 95°C for 15 s. To ensure reliability, each experiment included three biological and three technical replicates. Relative expression for each gene was calculated using the comparative Ct method (2^−ΔΔCt^) (Livak and Schmittgen, 2001). *CoxI* (GenBank accession number: FJ977926.1) was used for normalization (Supplementary Figure S2).

### *Agasicles hygrophila* Ovary Development and Fecundity After RNAi

Ten 4-day-old female adults from each treatment group were randomly selected to observe ovary development. Ovaries were dissected under an Olympus stereomicroscope (SZX16, Olympus, Tokyo, Japan) using high-precision tweezers (IDEAL-TEK, Balerna, Switzerland). The dissected ovaries were washed three times with 1 × PBS. Photography was performed as described in Liu et al. (2017). Observation and measurement of ovarioles was done as described by Zhao et al. (2016). Each pair of *A. hygrophila* adults was observed and laid eggs were counted and collected once a day. The egg hatch rate was assessed for a total of 1000 eggs every 12 h for 6 days until the unhatched eggs started to rot (Zhao et al., 2016).

### Data Analysis

All analyses of experimental data were conducted with SPSS 18.0 (SPSS Inc., Chicago, IL, United States) and are shown as means ± SD (standard deviation). Experimental data were checked for normality and homoscedasticity, and if needed, were arcsine square-root or log-transformed before analysis. Egg hatching rates and ovariole lengths were arcsine-transformed. The number of eggs was square root-transformed. We performed the least significant difference (LSD) test after one-way analysis of variance (ANOVA) to analyze differences in *AhVgs* expression levels between different tissues and developmental stages, fecundity and egg hatch rate. *AhVgs* expression levels after injection and ovariole lengths were analyzed by Student’s *t*-test. *P*-values of 0.05 or lower were considered significant.

## Results

### Sequence and Structural Analysis of the AhVgs

**FIGURE 1:**
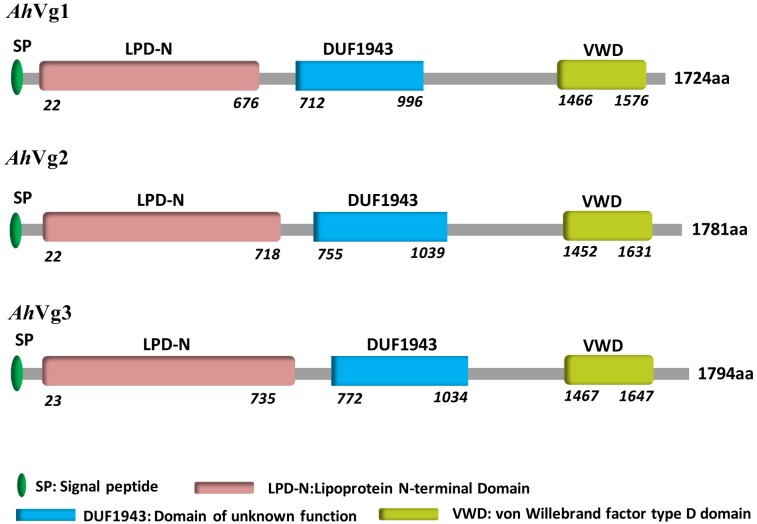
Domain architecture of *Agasicles hygrophila* vitellogenins (*Vgs*).

We obtained the full-length cDNAs of the three *A. hygrophila* genes *AhVg1*, *AhVg2*, and *AhVg3* by performing RACE-PCR and submitted them to NCBI (GenBank accession numbers: MH423679, MH423680, and MH423681). Information about these full-length *AhVg* cDNAs including their 5′- and 3′-untranslated regions (5′-UTRs and 3′-UTRs), ORFs, molecular weights and isoelectric points are presented in Table 1. Analyses of the *Ahvg1*, *AhVg2*, and *AhVg3* protein sequences revealed that all of them have three conserved domains similar to those in other insect *Vgs*: lipoprotein *N*-terminal domain (LPD_N), domain of unknown function 1943 (DUF1943), and von-Willebrand factor type D domain (VWD) (Figure 1). Additionally, *AhVg1*, *AhVg2*, and *AhVg3* all possess a signal peptide, and each protein is predicted to be cleaved between amino acids 16 and 17.

**Table 1:** Information about *Agasicles hygrophila* vitellogenin DNA and protein sequences.

Gene name	Full-length (bp)	ORF (bp)	5′-UTR (bp)	3′-UTR (bp)	AA (length)	Mw (kDa)	PI
*AhVg1*	5278	5175	38	64	1724	196.26	8.21
*AhVg2*	5512	5346	40	126	1781	201.98	6.42
*AhVg3*	5547	5385	40	122	1794	204.47	7.43

### Phylogenetic Analysis of AhVgs Sequences

**FIGURE 2:**
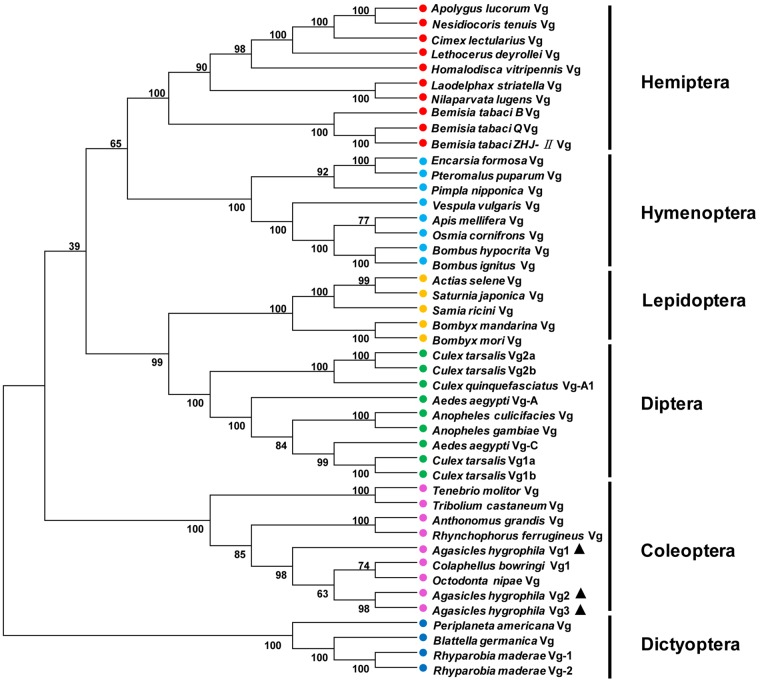
Unrooted consensus neighbor-joining trees for vitellogenin (Vg) proteins. Phylogenetic trees were generated with MEGA 5 using the amino acid sequences of Vgs from insect species from Coleoptera, Hemiptera, Dictyoptera, Lepidoptera, Hymenoptera, and Diptera.

A phylogenetic tree was constructed to evaluate the phylogenetic relationships of AhVgs with other insect Vgs (Figure 2). Vg sequences from Coleoptera were clustered in one branch, indicating that these Vg sequences had relatively similar amino acid sequences. The clade formed by Coleoptera Vgs was most closely related to those formed by Dictyoptera and Diptera Vgs, suggesting that Vgs in Coleoptera shared a more recent common ancestor with Dictyoptera and Diptera Vgs than with Vgs in other insect species.

### Expression Patterns of *AhVgs* Across Tissues and Developmental Stages

**FIGURE 3:**
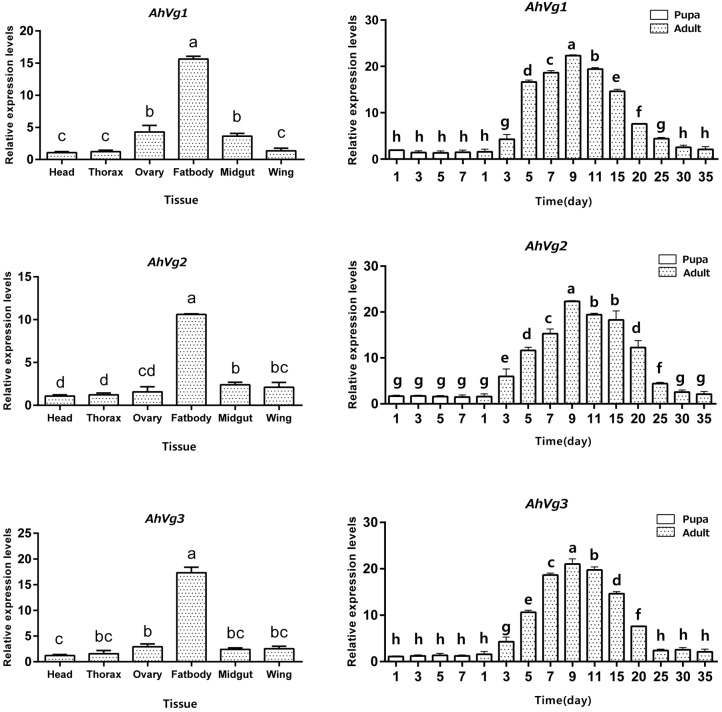
Tissue- and developmental stage-specific expression patterns. The relative mRNA levels were normalized to those of the *CoxI* gene and analyzed using the 2^−ΔΔCT^ method. All values are shown as the mean ± SD. The data were analyzed by the least significant difference (LSD) test after one-way analysis of variance (ANOVA). Different letters indicate significant differences between means (*P* < 0.05).

*AhVg1*, *AhVg2*, and *AhVg3* mRNA expression levels across different tissues and developmental stages of *A. hygrophila* were determined by qRT-PCR. *AhVg1*, *AhVg2*, and *AhVg3* were all mainly expressed in the female fat body (Figure 3); the expression level of each *Vg* was significantly higher in the fat body than in other tissues. During the pupa and adult stages, expression of *AhVgs* in the fat body was first detected in the newly emerged 3-day-old females (Figure 3). Additionally, *AhVg1*, *AhVg2*, and *AhVg3* were each maximally expressed in 9-day-old individuals, after which the expression level decreased significantly.

### Effects of dsRNA Injection on *AhVg* Expression

**FIGURE 4:**
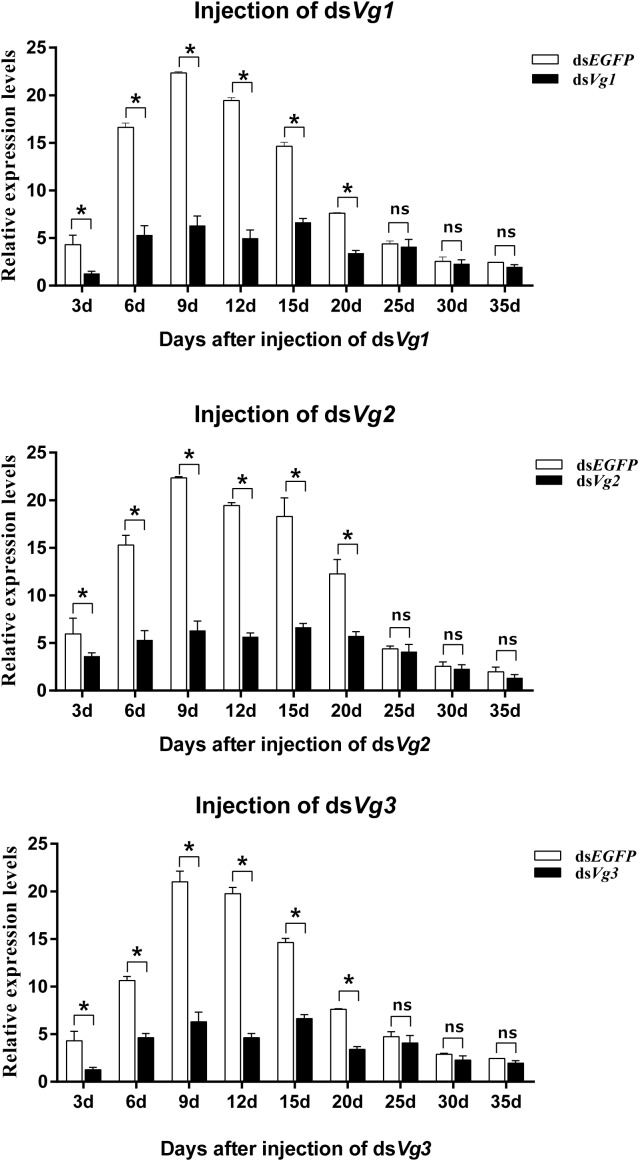
Relative expression levels of *AhVg1*, *AhVg2*, and *AhVg3* after injection of dsRNA into freshly emerged female *Agasicles hygrophila* adults. All values are shown as the mean ± SD. The data were analyzed by Student’s *t*-test. ^∗^*P* < 0.05. ns, not significant.

Injection of ds*AhVg1*, ds*AhVg2*, and ds*AhVg3* into freshly emerged *A. hygrophila* female adults significantly inhibited endogenous expression of *AhVg* mRNAs at each time point sampled (3, 6, 9, 12, 15, 20, 25, 30, and 35 days) (Figure 4). From 3 to 20 days after the injection of ds*AhVg1*, ds*AhVg2*, and ds*AhVg3*, the expression levels of these genes decreased significantly by 57.14–78.9% (Figure 4) compared with the ds*EGFP* control group. However, the decrease in expression levels of *AhVg1*, *AhVg2*, and *AhVg3* was not significant after 20 days, perhaps because of the timeliness of dsRNA.

### Knockdown of *AhVgs* Affected Female *A. hygrophila* Fecundity

**FIGURE 5:**
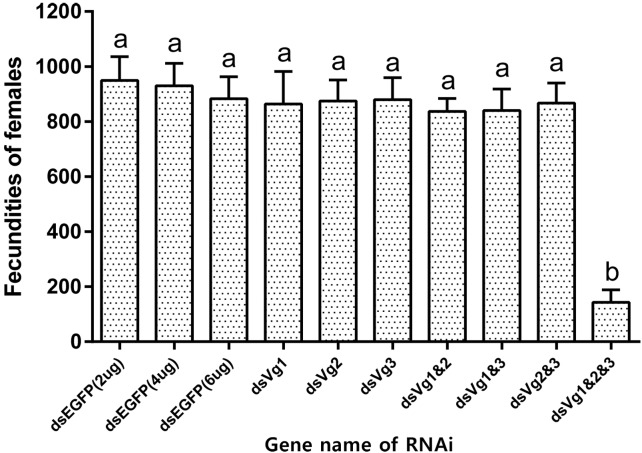
Fecundities of *Agasicles hygrophila* after injection of ds*AhVg1*, ds*AhVg2*, and/or ds*AhVg3*. Different amounts of ds*EGFP* were injected as a control. All values are shown as the mean ± SD. The data were analyzed by the least significant difference (LSD) test after one-way analysis of variance (ANOVA). Different letters indicate significant differences between means (*P* < 0.05).

**FIGURE 6:**
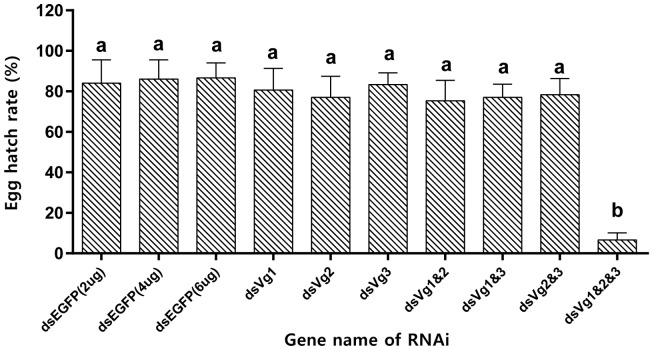
Egg hatch rate of *Agasicles hygrophila* offspring after injection with ds*AhVg1*, ds*AhVg2*, and/or ds*AhVg3*. Different amounts of ds*EGFP* were injected as a control. All values are shown as the mean ± SD. The data were analyzed by the least significant difference (LSD) test after one-way analysis of variance (ANOVA). Different letters indicate significant differences between means (*P* < 0.05).

After injection of ds*AhVg1*, ds*AhVg2*, and ds*AhVg3* into the abdomens of freshly emerged female adults, we observed that the number of laid eggs for the ds*AhVg1*&*AhVg2*&*AhVg3* group was significantly lower than that of the ds*EGFP* group, while the number of laid eggs for the other injection groups was not significantly different compared with the ds*EGFP* group (Figure 5). Additionally, the egg hatch rates of the eggs collected from the ds*AhVg1*&*AhVg2*&*AhVg3* group, but not the other groups, was obviously lower compared with the ds*EGFP* group (Figure 6). The finding that injection of one or two of the three *AhVg* genes had no distinct effect on *A. hygrophila* fecundity indicates that there is strong functional compensation among *AhVg1*, *AhVg2*, and *AhVg3*.

### Knockdown of *AhVgs* Inhibited Female *A. hygrophila* Ovarian Development

**FIGURE 7:**
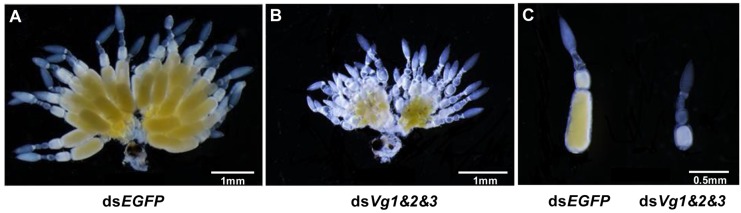
Effect of injection of ds*Vg1*&*Vg2*&*Vg3* on *Agasicles hygrophila* ovary development. **(A)** Image of an ovary that developed normally from an adult female injected with ds*EGFP*. **(B)** Image of an abnormal ovary from an adult injected with ds*Vg1*&*Vg2*&*Vg3*. **(C)** Image of ovarioles from **A**, **B**.

**FIGURE 8:**
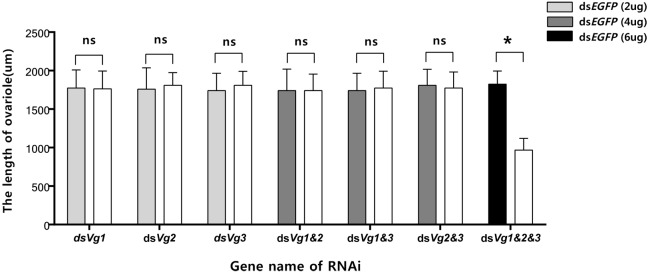
Lengths of the *Agasicles hygrophila* ovarioles after injection with ds*AhVg1*, ds*AhVg2*, and/or ds*AhVg3*. Different amounts of ds*EGFP* were injected as a control. All values are shown as the mean ± SD. The data were analyzed by Student’s *t*-test. ^∗^*P* < 0.05. ns, not significant.

Because the ds*AhVg1*&*AhVg2*&*AhVg3* group showed an obvious difference in the number of laid eggs and egg hatch rate compared with the ds*EGFP* group, we dissected the ovaries of 4-day-old female adults from these two groups to observe ovary development. The ovaries dissected from the triple injection group showed a reduction in deposition of yolk protein and a decrease in oocyte yolk uptake compared with the ds*EGFP* group (Figure 7). The lengths of the ovarioles of the ds*AhVg1*&*AhVg2*&*AhVg3* group were significantly shorter than those of the ds*EGFP* group, while the lengths of the ovarioles among other injection groups were not significantly different from those of the ds*EGFP* group (Figure 8). These results show that *A. hygrophila* ovarian development was only inhibited after injection with all three *AhVgs*.

## Discussion

The hemolymph proteins Vgs are the precursors of egg yolk, and are conserved across many eukaryotes, including insects (Tufail and Takeda, 2008). In insects, these large Vgs (∼200 kDa) are synthesized in the fat body, and the number of cleavage sites in these proteins and their expression patterns can vary (Tufail and Takeda, 2008; Tufail et al., 2014). In our study, we cloned and characterized three *Vg* genes from the alligatorweed flea beetle, *A. hygrophila*. This is the first study to investigate the functions of *AhVgs* in *A. hygrophila* reproduction. We observed inhibition of ovarian development and reduced fecundity only when silencing of all three *AhVgs*, which indicates that there is strong functional compensation among the three *AhVgs*. Our findings provide the basis for studying the functions of *Vg* genes in other insects.

Previous studies of *Vgs* have shown that they are members of a multi-gene family, with some insect species possessing more than one representative. For example, *Neoseiulus barkeri* has three *Vg* genes (Ding et al., 2018) and *Caenorhabditis elegans* expresses six *Vg* genes (Blumenthal et al., 1984). Our study represents the first attempt to clone the three *A. hygrophila* genes. The ORFs of *AhVg1*, *AhVg2*, and *AhVg3* were 5175, 5346, and 5385 bp, respectively. Sequence analysis of *AhVg1*, *AhVg2*, *AhVg3*, and *Vgs* of other insects revealed that all three *AhVgs* possess domains conserved in *Vgs*; LPD_N, DUF1943, and VWD. The calculated molecular weights of *AhVg1*, *AhVg2*, and *AhVg3* are all ∼200 kDa (Table 1), which is similar to the molecular weight of most insect Vgs, such as *Thitarodes pui* Vg (204.43 kDa; Wu et al., 2018), *Spodoptera litura* Vg (198.73 kDa; Shu et al., 2009), and *Harmonia axyridis* Vg (211.88 kDa; Zhang et al., 2017). Phylogenetic analysis indicated that *AhVgs* is homologous to other Coleoptera Vgs, including those from *Tribolium castaneum*, *Anthonomus grandis*, *Colaphellus bowringi*, and *Tenebrio molitor* (Figure 2). Additionally, the *AhVg2* and *AhVg3* sequences were more similar to each other than to *AhVg1* (Figure 2).

In our current study, *AhVg1*, *AhVg2*, and *AhVg3* showed similar expression patterns (Figure 3), with highest expression observed in the fat body, which is consistent with previous studies showing that insects typically synthesize Vg in the fat body (Ding et al., 2018). The VgR takes Vg up from the hemolymph and transports it into developing oocytes (Sappington and Raikhel, 1998). Thus, based on the expression pattern of *Vgs* in *A. hygrophila*, we speculate that the specific expression pattern of Vg indicates its function in synthesizing nutrition in the fat body. Although Vg protein synthesis in insects usually takes place in the fat body (Tufail and Takeda, 2008; Tufail et al., 2014), there are some exceptions. For instance, in *Rhodnius prolixus*, Vg can be synthesized in part in the follicle cells and in part in the fat body, and these both are later incorporated into the oocytes (Melo et al., 2000). After analysis of the expression levels of *AhVgs* at different stages, we found very high levels of *AhVgs* mRNA in adult insects but no expression at the pupal stage, so we conclude that *AhVg1*, *AhVg2*, and *AhVg3* expression is not required during the pupal stage. We propose that *AhVg* expression is upregulated during sexual maturation in *A. hygrophila*. Similar expression patterns have also been reported in other insects, such as *Bemisia tabaci* (Upadhyay et al., 2016) and *Actias selene* (Qian et al., 2011). However, upregulation of *Vg* expression in some insects was observed earlier, upregulation beginning during the pupal stage. For example, *Vg* expression was first detected during the late pupal stage in *Spodoptera litura* (Shu et al., 2009).

Gene silencing using RNAi is an efficient method to explore gene function (Hannon, 2002), and its use has proven successful in Coleoptera (Arakane et al., 2005; Shi et al., 2016). In our study, the target genes *AhVg1*, *AhVg2*, and *AhVg3* were silenced with high efficiency in *A. hygrophila*, but only suppression of all three *AhVgs* simultaneously negatively affected fecundity and ovary development, causing a significant difference in egg laying, egg hatch rates, and ovary development (Figure 5–8). This phenomenon indicates that *AhVg1*, *AhVg2*, and *AhVg3* play the same role in reproduction and that there is strong functional compensation among these three *AhVgs*. Previous studies indicated that successful reproduction in insects depends on two key steps: (1) creation and deposition of Vg and (2) the uptake of Vgs by the VgR into developing oocytes (Sappington and Raikhel, 1998). The silencing of *AhVgs* significantly affects reproduction in *A. hygrophila*, ultimately resulting in a decline in egg laying. Silencing of *Vgs* also results in depressed fecundity and abnormal ovary development in other insects. For instance, suppressing *Cimex lectularius Vg* caused ovarian tissue atrophy and reduced egg production (Moriyama et al., 2016). Additionally, Shang et al. (2018) demonstrated that *Aphis citricidus Vgs* are crucial for development during different stages.

## Conclusion

We present the first study of three *A. hygrophila Vg* genes. The amino acid sequences of the three AhVgs contain conserved domains (LPD_N, DUF1943, and VWD) found in Vg sequences from other insects. The tissue- and developmental stage-specific mRNA expression patterns were also similar to those in other insects. We found that all three *AhVgs* were most highly expressed in the adult stage. Our results provided further information about these highly conserved Vgs in insects. We showed via RNAi bioassays that *AhVgs* play important roles in *A. hygrophila* fecundity and ovary development, and that there is strong functional compensation among these three *AhVgs*. Whether these three *AhVg* genes have the same function in regulating reproduction in *A. hygrophila* needs to be further investigated.

## Data Availability

All datasets generated for this study are included in the manuscript and/or the Supplementary Files.

## Author Contributions

HZ and JG conceived and designed the experiments. HZ, JG, and YW performed the experiments. HZ, YL, JJ, MZ, and ZZ analyzed the data. HZ and JG wrote the manuscript.

## Conflict of Interest Statement

The authors declare that the research was conducted in the absence of any commercial or financial relationships that could be construed as a potential conflict of interest. The handling Editor declared a shared affiliation, though no other collaboration, with several of the authors, HZ, YW, YL, ZZ, and JG, at the time of review.

## Footnotes

1 http://www.ncbi.nlm.nih.gov/gorf/orfig.cgi
