# Supplementary material for: Identification and Expression Patterns of Three Vitellogenin Genes and Their Roles in Reproduction of the Alligatorweed Flea Beetle Agasicles hygrophila (Coleoptera: Chrysomelidae)
Source: Front Physiol. 2019 Apr 2;10:368. doi: 10.3389/fphys.2019.00368 (PMC6454870; doi:10.3389/fphys.2019.00368)
Supplement: Supplementary file 1 [file Data_Sheet_1.doc]

Supplementary Information for

**Identification and Expression Patterns of three Vitellogenin genes and their roles in reproduction of the alligatorweed flea beetle *Agasicles hygrophila* (Coleoptera: Chrysomelidae)**

Hong Zhang^1^, Yao Wang^1^,Yiran Liu^1^, Meiting Zhao^2^, Jisu Jin^3^, Zhongshi Zhou^1^, Jianying Guo^1^*

^1^ State Key Laboratory for Biology of Plant Diseases and Insect Pests, Institute of Plant Protection, Chinese Academy of Agricultural Sciences, Beijing, China

^2^ Ludong University, Yantai, Shandong, China

^3^ College of plant protection, Hunan Agricultural university, Changsha, Hunan, China.

* Corresponding author:

Jianying Guo, E-mail: [guojianying@caas.cn](mailto:guojianying@caas.cn)

Supplementary Figures

Supplementary Figure 1. Gel electrophoresis of ds*Vg1*, ds*Vg2*, and ds*Vg3*.

Supplementary Figure 2. Melt curves and standard curves for *CoxI*, *AhVg1*, *AhVg2*, and *AhVg3*.

Supplementary Tables

Supplementary Table 1. List of primers used for cDNA cloning, real-time quantitative PCR, and dsRNA synthesis.

Supplementary Table 2. Names and sequences of the Vg proteins used in Figure 2.

Supplementary Table 3. Concentrations of dsRNAs targeting *AhVg1*, *AhVg2*, and *AhVg3* before concentration adjustment.

Supplementary Table 4. Amount of ds*Vg1*, ds*Vg2*, and ds*Vg3* injected in each group.

Supplementary Figure 1. Gel electrophoresis of ds*Vg1*, ds*Vg2*, and ds*Vg3*.


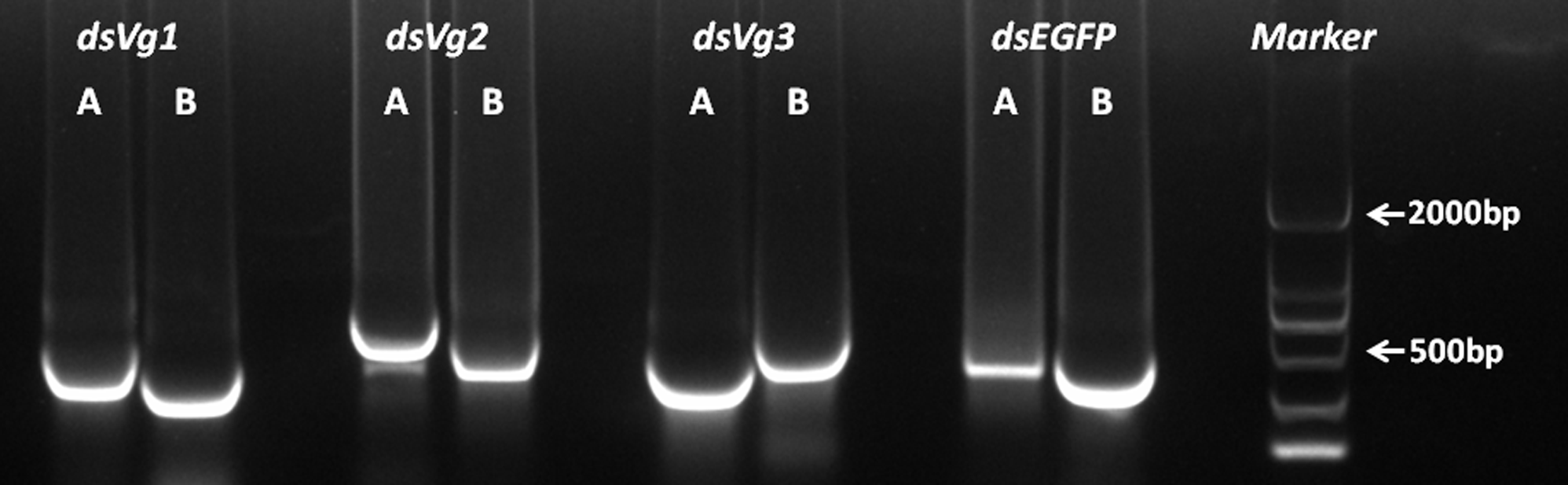


Supplementary Figure 2. Melt curves and standard curves for *CoxI*, *AhVg1*, *AhVg2*, and *AhVg3*. A: Melt curve and standard curve of *CoxI*; B: Melt curve and standard curve of *AhVg1*; C: Melt curve and standard curve of *AhVg2*; D: Melt curve and standard curve of *AhVg3* .


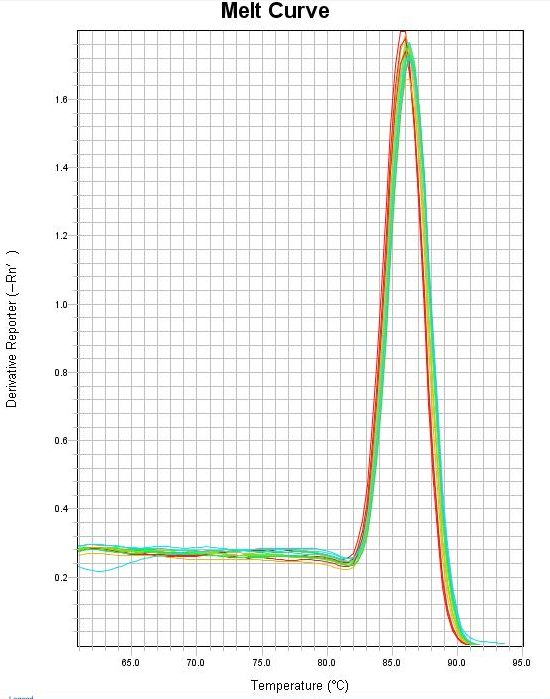


**A**


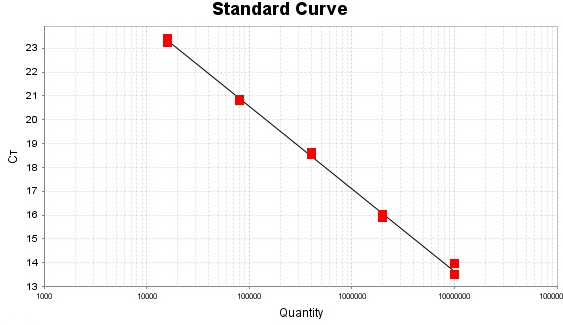

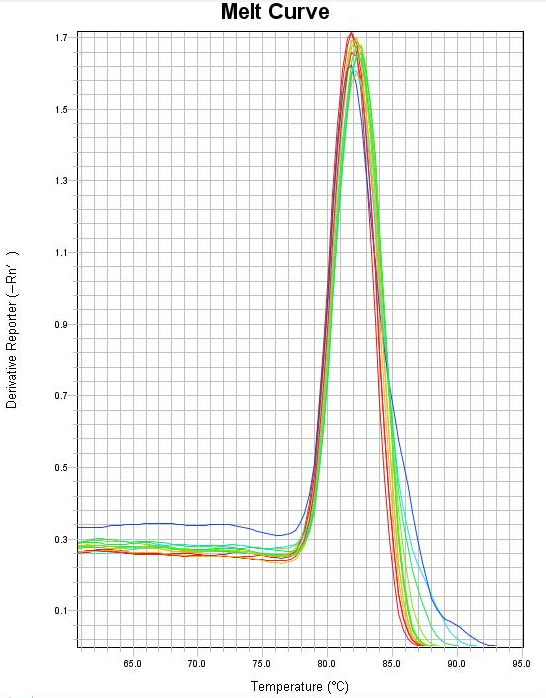

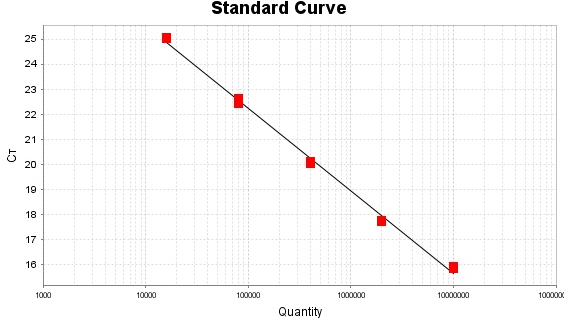


**B**


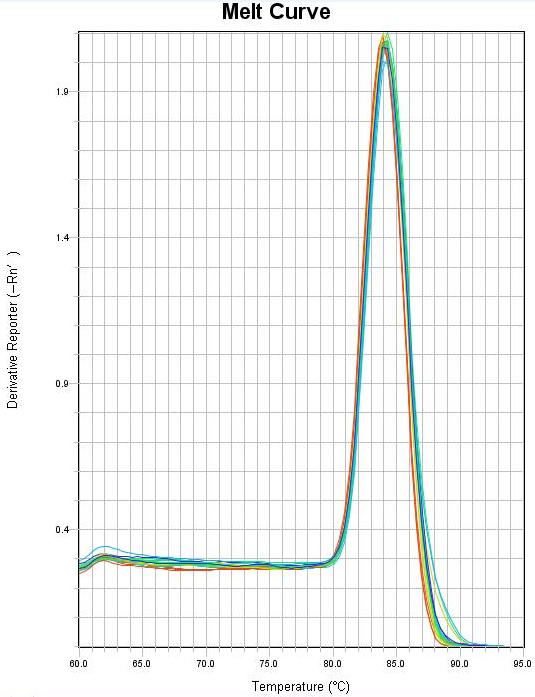

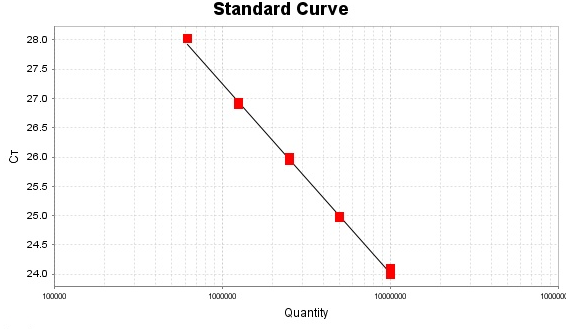


**C**


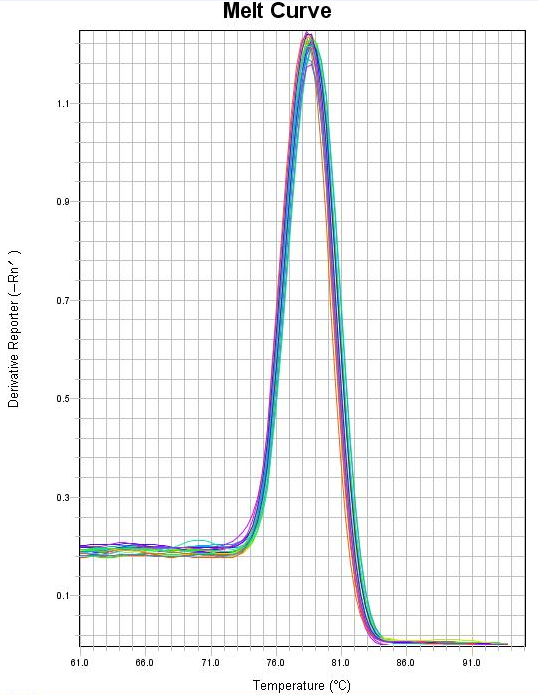

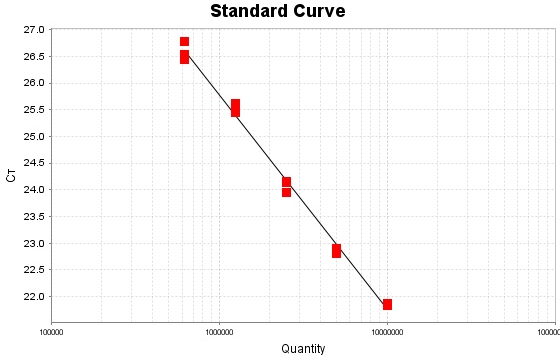


**D**

Supplementary Table 1. List of primers used for cDNA cloning, real-time quantitative PCR, and dsRNA synthesis.

| Name of Primer | Primer sequences (5’-3’) | Length of PCR product |
| --- | --- | --- |
| *AhVg1*-F | AATGGTTTAATTACTGATTTAAT | 4693 bp |
| *AhVg1*-R | GGAAATAGCCCATGGTTTGG |
| *AhVg2*-F | CATACGCATCTCAAAACC | 4263 bp |
| *AhVg2*-R | GGACGTGGAACACAGGATGG |
| *AhVg3*-F | CTGGAAAGGAGTACGTC | 5207 bp |
| *AhVg3*-R | AGCTTCGGGGCTGTGACGG |
| *AhVg1-*5’RACE | TATACTTGGAACTTGGCTGAATT | 521 bp |
| *AhVg1-*3’RACE | GAAGATCCGCAAGGCAACCAATG | 340 bp |
| *AhVg2-*5’RACE | CTGTCAATGTTCTTCCTTCAATCT | 155 bp |
| *AhVg2-*3’RACE | AACAAGAAATGGAAGAGGGAAACA | 1535 bp |
| *AhVg3-*5’RACE | GAAGTTCTCCCATAATTTCGTCGTA | 290 bp |
| *AhVg3-*3’RACE | AGATTCGTGGAAGATAACAAGAAGAC | 362 bp |
| qPCR*-AhCoxI-*F | TTAACGGGAGTGGTCTTAG | 107 bp |
| qPCR*-AhCoxI-*R | GCAAATACAGCTCCTATAGATAG |
| qPCR*-AhVg1-*F | GAGATGTCTACCGAAGGATT | 180 bp |
| qPCR*-AhVg1-*R | CAAGTTGATTGTTGGATGGA |
| qPCR*-AhVg2-*F | AAGGTCCAGAAGCAGTTAT | 149 bp |
| qPCR*-AhVg2-*R | ATCAGCGAGTGTAGCAATA |
| qPCR*-AhVg3-*F | GGACTGAGGAAGGAGAAC | 119 bp |
| qPCR*-AhVg3-*R | AATAGAGCGGACCAAGA |
| ds*EGFP-*A-F | TAATACGACTCACTATAGGGCATGAAGCAGCACGACTT | 433 bp |
| ds*EGFP-*A-R | TAATACGACTCACTATAGGGCAGCAGGACCATGTGATC |
| ds*EGFP-*B-F | TAATACGACTCACTATAGGGGAGGCCCGCACCGATCG | 412 bp |
| ds*EGFP-*B-R | TAATACGACTCACTATAGGGCCTCGGCGCGGGTCTTGTAG |
| ds*Vg1-*A-F | TAATACGACTCACTATAGGGCGCATTATCCTATAAACGCTTTC | 312 bp |
| ds*Vg1-*A-R | TAATACGACTCACTATAGGGCAATAATACGAATTAGAACG |
| ds*Vg1-*B-F | TAATACGACTCACTATAGGGCGCCCATTAGAATACTGTCAATAG | 245 bp |
| ds*Vg1-*B-R | TAATACGACTCACTATAGGGTTGAGATAGTTCAGGTTTAGA |
| ds*Vg2-*A-F | TAATACGACTCACTATAGGGCCACAGATGAGCGAACAC | 483 bp |
| ds*Vg2-*A-R | TAATACGACTCACTATAGGGTGTTCTCATTCTGTCGTA |
| ds*Vg2-*B-F | TAATACGACTCACTATAGGGGGTCCAGTATCAGCATTG | 363 bp |
| ds*Vg2-*B-R | TAATACGACTCACTATAGGGTTACTTGATTAGGAGCAT |
| ds*Vg3-*A-F | TAATACGACTCACTATAGGGATCTAATTCGCAGAGTGTA | 292 bp |
| ds*Vg3-*A-R | TAATACGACTCACTATAGGGGCCAAGACCATTAACAAG |
| ds*Vg3-*B-F | TAATACGACTCACTATAGGGCACAAGAACGAGCAATCT | 398 bp |
| ds*Vg3-*B-R | TAATACGACTCACTATAGGGTTTCCACAAAGTCCAAGA |

Supplementary Table 2. Names and sequences of the Vg proteins used in Figure 2.

>AFW97644.1 vitellogenin [Apolygus lucorum]

MQWTPFLLLVAVGLTAAEHGWKSGSQYKYQVRGRSMAGLQQVADQYVGIVMKAKLQVTPKSDSELSLQVQDAQYADVHANLTGGWNGDIPEEKLHYQQLPINNKPFELKFKNGVVDKMVVDKNLPTWELNMLKAIASQLQVDTKAENLKSSRINSLPNKDQAYGVYKTMEDTVAGEVETIYDISPLPQYVIQSRPQLAPLPKLRGDGQLIDIVKTRNFSNAEQRMAYHYGFTGLTDWEPASNQMGNFLSRSSVSRVIISGSLERYTIQSSVTTDKIAIAPHLYNNQKGVVGSRMNLTLESVNPSSGSPSSVQNPRSVKNLVYEYNAASQQEGAQNHNAYKQYQSSEDSSSSSSSDSSSSSSSSSSGSSSSDSSSSASNSTSASLSDSSSSSSSDSSSSSSESSQANNNKAHQHSQQNPSKSHRSQERTSKNRLRRSIPAAKNALNENSSSSSSSSNSSESNSSSPSSSSSSSGSSESNERLSLLPGQHGAGHKSQRNSSDSSSSSSSSSSSSSSSSSSSSSSSDSDSSSSSSSSSTSSSEEFWQPKPKLNMPPQTPFLPYFIGYQGNSIQASKQIDGVQIVQKLAKQIGAEVQQPNAIPGDNTLSKFEIMARVIRTMSADQLKKATEHIYYPYSKASPKSSQDAQSYQAWTSFRDAVAQAGTGPALLTLKDWIKSGKVEGQEAAELVSAVQNTARTPTPEYMDAFFNLATSEEACRQWFLNTSAILSFSNLVRKAQVNNDTAHNRYPSHVFGRLYPRKQAQKAVAEKYIPYLQTQLRKAVSQSDSPKIQVYIRALGNMAHPKILSVFEPYLEGKEPMSDFQRLTIVASMDKMTKTYPKLARSVLFKIYQNAGDAPEVRVAAVMQLMKTNPPAQILQRMAENTNSDHSKQVNSAVKSAIESAARLRTPSAHELAQNAKSAVNMLTPKNYGAQYSKNALRSYIVQEQQLGYQSQYTTIQSGDSIMPSSMFWALRKNLGGYKRQEFQASYMTSSADDLMDLLCDQFESQQESSSHKKSGSARRSQPSSGSSHWSVDKVANMLNIETDDAEQLEGNILISALSGKKFFAFDNHTIEQLPRLAKKAASKLREGQHFNYTKMYNQFSAKIGFPTATGLPFVFSFTVPTYAYVGGSIQAKSHPDISDSKDSIKIPKSINASADVEITYSAKARGKMGFVTPYNHKRYTAALHKNIHVHVPLRVAFDIDVENNKIAAKVQPLDANHKHKLFEYGTVAYTAKHDILDMQPELNANNDVEIVHVRSPRRNETTVGQDSTGFAFDVKVHSEQKFLDWATPLNALRRHDAISALLYTTAEKSINNNNVTITYNPEKSSAKSAQITASYDSEDETNNSSGESGNHQSHNKHRGSQERGSSSGNAAAEAAAASSSPDSAQRKEQFLRKAGAGIQDSYAQMVDMSIQFNGEHKAQYVATAAYASSGVSEKNRFLFYIGMSPARNGKDYQIALDVEAKMPNVPLVNYRKALEADASSHISAQLNFGERGSSGAQVSLQGKLVQTSERRDYVRHHPVSALCEQQMQQGNYIQQACRNATAAANMLDQYRFTINYEKVPESVKNATYKAYALARYFGNLYVSESIVNPNNKQGQVEIQVNLGKDLQSVNVSMAAPAISASFQNVPLNPYVAAAVAVHPEYNAADRVGQHLLLSQQFPTCSIDKNQATSFDNKSYPINLGGSWHVMAYWEPKSHFDSNSASSASSSEQQAFTILVRQSGSDKKEIIAVLGNDIVEILPQSEGQKAGIVKFNGKKATFDVQSSDTFQDENGNTVIQVFALPDGTVRVLGQKHGLEIMFDGQRVKLQVSNSYRGQMRGLCGVFDGEPVNDFTSPKNCILRNPYEFAASYAIPDSSLTSPAKELRQKAENADCYRQTVMLGDVISENEAGRSTKRSSSKSSNSIGNRNSKMSSPARLRVLVIHANGQTCFSTRPQLSCQSQSQEANTAQKNVDFHCVSDATAAKRWEDQIKRGASPDFSKKGANYKHSMKLPSRCNA

>ADU04392.1 vitellogenin [Bemisia tabaci B]

MWTPVLLCLLVAAANAQYGWKNGNLYKYEVNGRTLTALNQVADQYAGVLFRANFIVQPLSSDRLSAQIQNAEIAQVHTELPAGYESHIPSSQLNYKNMPLSEQPFEINLKQGVVSNLRVNKNVSDWELNIIKAVVSQIQVDTQGQNLKKSSHNQLPKENKPYGVYKTMEDSVTGECETLYDVSPLPEITLQTRPWLVPFPKLRENGQVIDIVKATNYSKCEERSAYHFGITGLTNWKPASNQMGQFLSRSNINRVIISGNVKQYHIQSSVSTNKIVISPQMYESQKGMVVSVMNLXLASFHQSNGSPRMVSNARKINNLVYDYNAASPNAYAQHYNNNGASSSSSSSSSSSSDSSSSSSSSSSSSSSSSSSSSSSSSEEDQYYRRHNNNNRNNNNNNNNNNNRNNNHNNNNNANDNNNSANNSNNNNSNNSANNNNNNNKNNNRNNDENVSRSRSRRDISQYKYNSFENNNNNDNEDEKNNSGRNGHNGHNGNNGHNGNNGHNGHNGHNGNNGQNNSNGSSSSSSSSSSSSSGSSSSEENNSRYNNGKFASFARHNGSGSSSSSSSPDSSDSSSSSSSSSSSSSSSSSSSSSSSSSSEDNSSFGSSVSSSSEEDYEPRPSTYKAPQTPFFPYFIGNYGNSIQSAKQVNGVALARKLAQEIAEELNDPRQITQKSTLAKFNMLVEELRTLDAKQMEQASQELHFNSAQASSHSRQDALKSLAWKSFCDALVEAGTGPAFLQIQKIIEHQQVSDAEAARMISRLPVTARFPDKEYMNSFFNFVRSNNVQHQNQLNETALLAFAELCRKADVNARNAHNYYPVHVYGRVLPEHAKAVAHQYLPYYEQNLKRAVANGDSRKIQAYIRAIGNFAHPKILEVFEPYLEGKVPISNFQRTVMVLSLNELARVYPNLARNVLFKIYQNTQENQGVRVAAVFLIFGTNPSAQTLQRMAQFTNEDQDQQVNAAVKSALENAAKAHSESRQELAQAAQSAIALLSPKTYGLQYSKKWLRDYIVKEENLAYRVSADMIQSEDSLIPNQVYVALHRYLGGFAQRVASFRAMTSSASDLVEKIQEQFTNGEEYQQQSEMNQQFSAEQIFRQFNIKPDYPQEVEALLQYTVFGAKRWAFFDEEFFNQIPRRLNDAPSKVQNGQSFNSTKFYNDISLSLAFPTATGLPFSYTLKVPTLVQAGGEVQARVQGHNSNNNNNNNNLFRIPEAVNVTAEIEIVYATELKSELGFVTPFNHERYVAGLAKNIFVNIPVKVAANVDIANTKVEFYMKPMNNQNEQKIFHYGSYPYTAIQNIFDFRPLQENENTKYIFANENKNKFEKVYGEEKTGFAFRCQYKGDQQSFQFADFYNFAKRNDFFSAAFFPWAEKTIQYNNFDVYYDPARSAAKSAKFALNYANKYANKENNNEGNSNNHNNNQNDAVPSSYQPDNEQRMNQFASRAQSGVQSANIDVIGISAQFFGQKNADYVATLAYARSPVAEKARFLFYAGANNANNNKNKVAVDATAYMPNVPLVNAAQAFNADANSRFYANVKSGENLDNGAQFQFQANMKQSQEFRDYFRQSQMYKQCSQQMEQGEYMMPACRNATVVANRLNEAHFSINFDKVSDAVKNYTYQAFAYARHLGYQYQSENFGNPNGQHNKIDGYFKFSPKFDFAQFYFNAPSVAASFKNVPVHQYVADFFAPHPVYSGFDRLMQDTFQAKYQAACVADKMHATTFDNKTYPLHMQQNNWYVLMTYVNRNNYYNNQYNSYFQGNNKNQYSYRDYNQKRFYTTVYARQNSNGQKELKIVLNNGEYEIFMQPASSRAGLHSSNSGKNNAAIKVFINKQEQQFNDKQFTDFHGHNGKIYAQFYALPDGAIRFFAPQSGLQAIYDGARIKIQAANQYRGAVRGMCGTYSNQYADDFTAPQNCVYKNPEDFAAVYAVIDSSSPSQVKSQKERAQQNFCAHKNNQFGNYVSRSDAGYGYKYNNNDKYYESAYKNTKYYDPSNSQYNKYYKNNKYAKDQDSSYSSSSSSSSDSSSSSSSDSSSSSSSSSSSSSSSSSDSSSSQSNDNNRNNNNRNNNNRQQQQQQPQQQQQPQQQQQQQQPQQQPQQQPQQQQQQQQQQPRSQLRTEKPKRTLYPQTVPCHQPRRRHVLHHQRYPNLQIPSQARWKRQENG

>ADU04394.1 vitellogenin, partial [Bemisia tabaci Q]

MWTPVLLCLLVAAANAQYGWKNGNLYKYEINGRTLTALNQVADQYAGVLFRANFYVQPFSSDRLSAYIQNAETAQVHAELPSGYESHIPSSQLNYKSMPLSHEPFEIYLKKGVVSNLRVNKNVSDWELNIIKAVVSQIQVDTQGQNLKKSSHNQLPKENKPYGVYKTMEDSVTGECETLYDVSPLPEITLQTKPWLVPFPNFRENGQFIDIVKTTNYSKCEERSAYHFGITGLTNWKPASNQMGQFLSRSNINRVVISGNVKYYTIQSSVSTNKIVISPQMYESQKGMVISVMNMTLASFHQANGSPRNGHNGHNGNNGHNGNNGHNGHNGNNGQNNSNGSSSSSSSSSSSSSGSSSSEENNNNNDNEDEKNNSGAYAQHYNNNGASSSSSSSSSSSSDSSSSSSSSSSSSSSSSSSSSSSSSEEDQYYRRHNNNNRNNNNNNNNNNNRNNNHNNNNNNANDNNNSANNNNNNNSANNNNNRNNDENVSRSRSRRDISQYKYNSFENNNNNDNEDEKNNSGRNGHNGHNGNNGHNGNNGHNGHNGNNGQNNSNGSSSSSSSSSSSSSGSSSSEENNSRYNNGKFASFARHNGSGSSSSSSSPDSSDSSSSSSSSSSSSSSSSSSSSSSSSSSEDNSSFGSSVSSSSEEDYEPRPSMYKAPQTPFFPYFIGNYGNSIQSAKQVNGVALARKLAQEIAEELNDPRQITQKSTLAKFNMLVEELRTLDAKQMEQASQELHFNSAQASSHSRQDALKSLAWKSFCDALVEAGTGPAFLQIQKIIPYTTMFNTRTNSTKLLSLLSLSFARKADVNARNAHNYYPVHVYGRVLPEHAKAVAHQYLPYYEQNLKRAVANGDSRKIQAYIRAIGNFAHPKILEVFEPYLEGKVPISNFQRTVMVLSLNELARVYPNLARNVLFKIYQNTQENQEVRVAAVFLIFGTNPSAQTLQRMAQFTNEDQDQQVNAAVKSALENAAKAHSESRQELAQAAQSAIALLSPKAYGLQYSKKWLRDYIVKEENLAYRVSADMIQSEDSLIPNQVYVALHRYLGGFAQRVASFRAMTSSASDLVEKIQEQFTNGEEYQQQSEMNQQFSAEQIFRQFNIKPDYPQEVEALLQYTVFGAKRWAFFDEEFFNQIPRRLNDALSKVQNGQSFNSTKFYNDISLSLAFPTATGLPFSYTLKVPTLVQAGGEVQARVQGHNSNNNNNLFRIPEAVNVTAEIEIVYATELKSELGFVTPFNHERYVAGLAKNIFVNIPVKVAANVDIANTKVEFYMKPMNNQNEQKIFHYGSYPYTAIQNIFDFRPLQENENTKYIFANENKNKFEKVYGEEKTGFAFRCQYKGDQQSFQFADFYNFAKRNDFFSAAFFPWAEKTIQYNNFDGYYDPARSAAKSAKFALNYANKYANKENNNEGNSNNHNNNHNDAVPSSYQPDNEQRMNQFASRAQSGVQSANIDVIDISAQFFGQKNADYVATLAYARSPVAEKARFLFYAGANNANNNKNKVAVDATAYMPNVPLVNAAQAFNADANSRLYVNVKSGENLDNGAQFQFQANMKQSQEFRDYFRQSQMYKQCSQQMEQGEYMMPACRNATVAANRLNEAHFSINFDKVSDAVKNYTYQAFAYARHLGYQYQSENFGNPNGQHNKIDGYFKFSPKFDFAQFYFNAPSVAASFKNVPVHQYVADFFAPHPVYSGFDRLMQDTFQAKYQAACVADKMHATTFDNKTYPLHMQQNNWYVLMAYVNRNNYYNNQYNSYFQGNNKNQYSYRDYNQKRFYTTDYARQTSNGQKELKIVLNNGEYEIFMQPASSQAGLHSSNSGKNNAAIKVFINKQEQQFNDKQFTDFHGHNGKIYAQFYALPDGAIRFFAPQSGLQAIYDGARIKIQAANQYRGAVRGMCGTYSNQYADTSPPLKTVSTRTQKDFAASYAVIDSSSPSQVKSQKERAQQNFCARKNNQFGNYVSRSDAGYGYKYNNNDKYYESAYKNTKYYDPSNSQYNKYYKNNKYAKDQDSSYSSSSSSSSDSSSSSSSDSSSSSSSSSPSSSSSSSDSSSSSSSSQSNDNNRNNNNRNNSNNNNNRNNNRNNNNNNNSSSSQEASYEQRNQNGPSIRKLYRAINQGDDMCFTINAIPTCRYPAKPVGSAKKMVDFYCAPKSSSEAQHFSKLIAKGAAPSQLSLKKPNQKFEVNIPEYCVA

>ADU04393.1 vitellogenin [Bemisia tabaci ZHJ-Ⅱ]

MWTPALLCLLVAAANAQYGWKNGNLYKYEINGRTLTALNQVADQYAGVLFRANFYVQPFSSDRLSAYIQNAETAQVHAELPSGYESHIPSSQLNYKSMPLSHEPFEIYLKKGVVSNLRVNKNVSDWELNIIKAVVSQIQVDTQGQNLKKSSHNQLPKENKPYGVYKTMEDSVTGECETLYDVSPLPEITLQTKPWLVPFPNFRENGQFIDIVKTTNYSKCEERSAYHFGITGLTNWKPASNQMGQFLSRSNINRVVISGNVKYYTIQSSVSTNKIVISPQMYESQKGMVISVMNMTLASFHQANGSPRSVNNYRKVNNLVYDYMAASPNAYAQHYNNNGASSSSPSSSSSSSDSSSSSSSSSSSSSSSSSSSSSSSSEEDQYYRRHNNNNRNNNNNNNNNNNNNRNNNHNNNNNANDNNNSANNNNNNNNNNSANNNNNNNKNNNRNNDENVSRSRSRRDISQYKYNSFENNNNNDNEDEKNNSGRNGHNGHNGNNGHNGNNGHNGHNGHNGNNGQNNSNGSSSSSSSSSSSSSGSSSSEENNSRYNNGKFASFARHNGSGSSSSSSSPDAYAQHYNNNGASSSSSSSSSSSSSSEENSSFGSSVSSSSEEDYEPRPSMYKAPQTPFFPYFIGNYGNSIQSAKQVNGVALARKLAQEIAEELNDPRQITQKSTLAKFNMLVEELRTLDAKQMEQASQELHFNSAQASSHSRQDALKSLAWKSFCDALVEAGTGPAFLQMQKIIEHQQVSDAEAARMISRLPVTARFPDKEYMNSFFNFVRSNNVQHQNQLNETALLAFAELCRKADVNARNAHNYYPVHVYGRVLPEHAKAVAHQYLPYYEQNLKRAVANGDSRKIQAYIRAIGNFAHPKILEVFEPYLEGKVPISNFQRTVMVLSLNELARVYPNLARNVLFKIYQNTQENQEVRVAAVFLIFGTNPSAQTLQRMAQFTNEDQDQQVNAAVKSALENAAKAHSESRQELAQAAQSAIALLSPKTYGLQYSKKWLRDYIVKEENLAYRVSADMIQSEDSLIPNQVYVALHRYLGGFAQRVASFRAMTSSASDLVEKIQEQFTNGEEYQQQSEMNQQFSAEQIFRQFNIKPDYPQEVEALLQYTVFGAKRWAFFDEEFFNQIPRRLNDALSKVQNGQSFNSTKFYNDISLSIAFPTATGLPFSYTLKVPTLVQAGGEVQARVQGHNSNNNNNNNLFRIPEAVNVTAEIEIVYATELKSELGFVTPFNHERYVAGLAKNIFVNIPVKVAANVDIANTKVEFYMKPMNNQNEQKIFHYGSYPYTAIQNIFDFRPLQENENTKYIFANANKNKFEKVYGEEKTGFAFRCQYKGDQQSFQFADFYNFAKRNDFFSAAFFPWAEKTIQYNNFDVYYDPARSAAKSAKFALNYANKYANKENNNEGNSNNHNNNQNDAVPSSYQPDNEQRMNQFASRAQSGVQSANIDVIDISAQFFGQKNADYVATLAYARSPVAEKARFLFYAGANNANNNKNKVAVDATAYMPNVPLVNAAQAFNADANSRFYANVKSGENLDNGAQFQFQANMKQSQEFRDYFRQSQMYKQCSQQMEQGEYMMPACRNATVAANRLNEAHFSINFDKVSDAVKNYTYQAFAYARHLGYQYQSENFGNPNGQHNKIDGYFKFSPNFDFAQFYFNAPSVAASFKNVPVHQYVADFFAPHPVYSGFDRLMQDTFQAKYQAACVADKMHATTFDNRTFPAHFQNNWYVLMAYMNRNNYYNNNFNQYLQQNKNQHSYRDYNEKRFYTTVYARQNSNGQKELKIVLNNGEYEIFMQPASSQAGFHSSNSGKNNAAIKVFINKQEQQFTDKQFTDFHGHNGKIYAQFYALPDGAIRFFAPQSGLQAIYDGARIKIQAANQYRGAVRGMCGTYSNQYADDFTAPQNCVYKNPEDFAAVYAVVDSSSPSQVKSQKERAQQNFCARKNNQFGNYVSRSDAGYGYKYNNNDKYYESAYKNTKYYDPSNSQYNKYYKNNKYAKDQDSSYSSSSSSSSDSSSSSSSDSSSSSSSSSPSSSSSSSDSSSSSSSSQSNDNNRNNNNRNNSNNNNNRNNNRNNNNNNNSSSSQEASYEQRNQNGPSIRKLYRAINQGDDMCFTINAIPTCRYPAKPVGSAKKMVDFYCAPKSSSEAQHFSKLIAKGAAPSQLSLKKPNQKFEVNIPEYCVA

>BAU36889.1 vitellogenin [Cimex lectularius]

MWTKILLLALVGLASAEIAWKVGNQYKYEIRGRTVTGLHQVADQYAGMLMKGKLTVQPKSENVITLQIGQAQYADVHANLTGGWSSYIPDNKLNYRQLPFSNKPFELHLKNGVVDKMVVASSVATWELNMLKAIASQLQVDTQGEHLMKSKINQVPNKGDHMGVYKTMEDTVTGLCETIYDVSPLPEYVLQSRPYLAPLTHYRGDGQFIDIVKTMNYSNCDQRVAYHFGISGLTDWEPASNQMGTFMSRSSQTRVIVSGSLSHYTIQSSVTTNKIVLAPHLYNSQKGIVASRMNLTLEAVSQSGNVPSVPNPRTVKNLVYEYNAATEQEDKQNHNAYKVYNSREETSRPSSSSSSSSSSESSEKIQSSHKTATNTHQQTRSRSRRSVVRDEHTIEDSYSSSSSSESHEQLGQMKQGQKRLSSASSSSESSMSSSEEYWQPEPELQKAPTSPFLPYFIGYQGNSIQAARQVDVVQQVQKLAQQIGSEVHKPNGLTGENTLTKFVILTRVLQTMTVDQLKRATEQLYYPYSKASPRSTSDAERYQAWVAFRDAMSQAGTGPALLCLKEWINTGKVQYEEAAELVAVLPQTARYPTNEYMNAFFDLATSEKVTKQRFLNTSAIFAFTSLVRKAQVDKDTTYNRYPVYTYGKFALNNTKDVVNKYIPYLEKQLTKAISQGDSIKIQVYIRALGNTAHPKILSVFEPYLEGQQHVSDYQRLTMVASLDKMTKVYPTRVRSVLYQIYQNTGDTSEVRVAAVMQLMKTNPPAQLLQRMAQFTNSDHSKQVIAAVQSAIKSAASLTTPEHYELAENAKSAVNMLNYRDFGLQYSTHYIRSYLVAQEPQHGHLSYKGDFTAIEGEDSLVPSSVFYNLRENLGGFKRGQYQMSYMTSSADELMELLSNQFTCEHCRNSKSSHSKPDNTWSFEKIAKMLNVVVNKPEQVEGNLFLSTFGGKRFFAFDNHTVEQLPNIAKYVAQSLHQGQHFNFTKMYSKSGFKIGFPTAMGLPFLYSYDVPTFAYLGGEMRAQSHPDIASYPKNEIPVPKTVNVTTDMEFTYVVKTQGKMGFVTPFDNQRYIAGLQRNIHLHLPIRARVDVDVENNKVWAKLESFEQNHKHKLFEYSTVAYTAKHDILTFTPVVDGPHAEHIHVRPVQRRESTVGHESTGFAFDVKAETEQKFYDFATVYNALKRHDPLSALVYGHFEQSISNNNFTVVYNPQRSTAQYANFTFVYGKTGHSAEKGVHSSHNKHRAANVRNSAAQSDSKPDSLERQAQFLQNAAAGIQDYTAHMVDMSVQFEGQSKAKYVATASYASSPVSDKSRFLFFVSKTPAKQTYTTKPYEAALELTATSPNVPLVNFKKALETQPTSQINGEFSFGEKASSGAKVTFQGKLSQTNERRDFVREHPVSKLCESEMREGNYIQPACRNATASANFFDQYKFTFHYEQVPQVFKNFTYKAYDLARYFAYPYVHEDTVSAKNQQGEVDVVVRFEKDLKAVNISVEAPTLTAQFRHLPVNRWVRPAVVFHPHYNTADRLGQTYFRAQQFPTCVVDKNLATTFDNKSFPVKLGDCWHVLAHSLGVFTHDPEDKYFGALVREHESDKKELVLVFGENVVEVKPTSSSDKVGVVKVNGQTAEFTQTKVAKFEDNFGYTFFQVYALPTGAVRMYSPLAGVEVVYDGARVKLQVSNTFRGQLRGLCGTFNGEDVDDFTCPNNLILKDPYLFAGSYAYADETCKGPAKEMHDKAQNAKGYKKYVVLGNVLNSRKSQHQKLPTSNIAEPSMKVERKPYRSVVKVVEYNGQTCFSARKMPECLPGYKTEGYEVRNEKFHCLTDNATAQRWRESVSKGNSLRFGDRKPNHQQNVQVPKKCVKN

>AAZ06771.1 vitellogenin [Homalodisca vitripennis]

MWTPLLLCLLAFAATHADNLAWKPNTQYEYAVRARTMAALHQVAPQYVGLVLNAKLYLQQASNNVVTLQLHNAQYANVHANLSQGWSTPIPESQRHYQPIPMSNKPFQLVYKNGVISRMVVSKGVPTWELNILKSIASQFQVDTQAENLQKSRINSLPTQETVNGVYKTMEDSVSGECETLYDISPLPKVVLQNKPQLAPMPHLQADGQLIDIVKTMNFSNCDVPSAYHFGITGLTNWEPASNQMGQFLARSSVSRIIIAGNLKRYSIQSSVTTNKIIASPFLYEKQNGMVVSRMNLTLVDVKSASSSPQSPPSPQVIKNLVYEYNPASHQENTQKQYREYNGNENLSSSSSSSSSSTSSDSSNSDSSSSSSSESQEQNNYQQKNRFARSASSRRNQQSNYNQDTSSFSSSSSSSSSSSSSDSDSSSSSSFSWDSSEEENWQPKPTLSQAPNFPFLPMFIANQGNSIQSAQEIQGVSVVKKIAQQIGYEMQSPSAIPGQKTLGMFTILTSVLRTMNSKQMEEATRELYFPLSRASSTSSSDAVKYQAWVAYRDAIAQTGTGPALLTIKEWIQSKKVQGEEAAQILAALPYSARFPNVEYMNTFFALATSSEVQHQHFLNTSAVLSFTVLARKAFVSNSTAHNRYPVHAFGALKDKNQQPVVQQYIPYFAGALKQAVQQEDSIKIRTMTLALGNLAHPKILDVFEPYLEGQKPMTQFQRLTVVASLRKLAQLYPKVARPVLFSIYQNTGEATEVRVAAFYALMKTNPPAQLLQRSAQFTNFDQNPQVISAVQSAIKTAAYNQKLPSELSKNAQAAVNLLNPKVMPAQYSRKNIRSYYNQQLNLGYKQAFSYIGSDDGFLPSNIFYSLRKQMGGFNHKAVGFAAMSSSLDVLTDLLEEQFSDAPSSSSHRSQKQPQQQGPWSFEKINNMLHIQPSEQEQVEASLLVSYMGGKRFFAIDNHTVELLPQLFKEAAASLRSGRSFNYTKMANTFALNVAFPTAMGLPFTFSLQIPTLLYIGGQAQAKSHPDLANGNNHEIQVPQTINASVDFQFVYSLQAQSSMGFVTPFNHQHYTAGVYKNFQVNLPIRAQVDLDIVNNKVAAKVQPLDQHNKQNLFEYSTVPYTSQHSVLQMAQNPQEQTHGNSNNKRVHVRHANQIHKTFGQDSTGYAFDFNYHSEQKFIDFAALYNEAHKFKDPIAVVMYACAEDSMNINNITFTYNPQKSSARYARFTAAYLDNSENDSQGQNYQGQNYRNSERSDSQQASPNEQVSASMAHPSSSAPDSQSRQQQFLQKVGQGIQDSDNIVVDVSAEFQGQNKAQYVATIAVATSSVSAKSRFLMFAEKNPVNSNKQGKMYVAAESSMPIVPAMNYKQALNADPTSYFNAELAFDDAKVQLKGKMQQSQARRQYLNNYPLAQKCQQQMQQGNTVLYACRNVTLQANVFDNFKMSVHYDKIPSYWRNVTYKAYAALRYAAYQYVSEDIISVQNPSNQIYFEANLAPNLRTVNFTMATPLLNAKLQNLSPPRYIQPFVWWHPQYTSFEMYANHIFKGQQFPTCVVDNNWAQTFDNKSYPIKLGKCWHAMFHYTPKEDPTSSESTNDYDEDEISILVQEASSSNEKELMIVLGGYNIYMQPTPGNSPAQVTVNGQQTPVSKSYLTELFDQNGNTLAQMYARPNGEVHFYAAQQDIKVQYDGTAVKVKAQNSYRSETRGLCGTFNTQPVDDFTTPQGYILQNPYEFAATYALESSSCQGPAKELKARAQQQIAGGHYSRNVVIYGNVVTDADAYHYSRSNSNKYGRLRILNKEKYVSCSQRRLMTMHKDGKQCFSVQPQLHCTDQCSPQGYVNKEVQFMCVLTSSVSEHWKKLVNRGINPDFSDKHAYFQTYNVQVPQSCQAN

>AGJ26478.1 vitellogenin [Laodelphax striatella]

MKGITLIFCVIAVAGVSASGSGPWNSNQLYHYKVQGRTLSAVQQAGALQYVGMHMKAEVSVAAKNENQAVFKISNAQYADVHQNLTEGWQQELRSNELQYKQLPLSQANQAFEVNYKQGSVRSLQVNRNTPTWELNMIKGIVSLFQVDVTGQNAIKSRRNILPQSDSNQQVSGSFKAMEDSVTGKRETHYDVDELPMRVVQQHPEIAPLAVQNSNQQQHQRIIQVVKSRNFSNCDNPVTYHFGFTQETDWEPASNQMGNLVNRASTSRVILSGQPNSFTIQSSVTQNEISISPFGYNKQKGVVGTLMNATLASMSHASGSPQSVQNPQKINDLVYEFNPASNNENNAANRRSSQSNRQNDADDSSSSSSSSSSSDSSSSSSSSSSSSSEENNKNGKNNNNNNWNKNKRNNDDDNNRHNSNDNKRRNSNDNRSQANDNDDDAYWRNQQKTKSRSRRSVLKNFNNDDDEDNNQNNKRYNNQNRNNNNDSSSSEDSNENNQNNKNRNNRQNNDNNNKYYNNQNNKNRNNNNDNDDSSSSSSSDSSSSSSSSSSSSSSSSSSSSSSSSSSSSDLDSSEENWQQKPDMNDAPSTPFLPHFVGVRGNSIQADKQVDIVNEAQKVAMRIGAQVQKPSAIPGQNTLTSFTILTRMIQTMSAKQIQEVKQRLFIDRNNANGKSSADAKKLQSWEAFKHATANAGTGPALQAIKNWVEKGDVRNEKAAELVAVLPRTARLPTDKYIKTFFEFATSSNVVNQKYLNSTIIIGFSEILRKAQVDSDTKHMRYGVHSFGHLTSKNDQSLQQEYMPYLEEKLKSAFEKGDSQKIIVHIQALGNTAHPRLLKTFEPYLEGKKSASRFQRLLMVASLYQMTRVHPTTARAVLYRIYKNPGEAPELRVAALHLLANTNPPAAMLQRMAQQTNWEQSKEVISATQSFIKSAANMDENPDSIELARNAQAAVDMLNPNDYGYSMSKNYLSSYVIDNIDKSYESQLSTIGSFDSIIPSSVFVNFMANDGGYKHQVFHHSAMFSSVNDLLELVNTQFKNNNNNNNSNNNRRNNRSGSNDNEDNQNNRNSNNEWTAENVLKALNIQKDQAEQLEGNILLTMLGGKRAFAINNHTIEKIPSIFKEAAQKLKHTSFNLTQFYSKNTMKVAFPTPMGLPFVYASSVPTMVYVGGETKVNSHPDLANGNNNYVNIPQYINMSADIEAVYSMQANSKFGTVAPFNHHEYYASVEKNLQFYFAVQTQADIDLENNEVQLRMQPLNKEEKQNVFQYSTVLFTTKSNILNFNPALQEDGTERVHVGKAKQIQKNFGRDSTGFAFEASYWSENGFGDLASLYEEVSKFDVQSALASPWAQSSLNPNNMTLTYRPSESTSKVAKFTVSYNDNSNNNNNNNNNSQNDHDSSNNNRAENINYSAAQPSSNAANSRSRQNEFLHKVASGISGADAMVVDVSAEFQGSHGQSSAQYVATLAMANSDASPNARMLFFASMDPANSGSKAQVCAAAASHFPNVPLMNFNDALKANPDSHITADIAFGEKCNAGGHIRADAKMSQTQEFQDFAKNRPMAKKCFQLMQQGQALEYACQNATKVANMLNNYEVSLKYDRVPNALKNATYNIYSALAQVAFPYLSENMFSQHSNPAGKIDLNARFNYNLRYFNASINSPFFTANFKNVEVNPAVRPLVIFHPSLNSLELMSYNENYDYPTCSVSKNSISTFDNKTYSADLEGWHVMFASTPKNYNDNSGRYSASNSQSNSFYKYKKVAILAKNSGSQRKAVKMLLGDNVIDITPSGSESNNNSPNANVQVNGNKMHIANNRLASFEDFDGETLVEISVNDNGEVQVQSPSHGIAVNHDGANFMIDADSYYRGEVRGLCGTYSGDKYTDFTTPKKCVLREAKLFAATYALPGSSNSNVEQLKRQADQVSCFKRHEILADVITSNDYDRSSSSSSNQRNNKNNNNNNNRYNKNSSMKYQTSDSSSSTDLIQDIKNNGDHVCFSIRPIPKCQHGSSPAGSSEKEVQYLCISQGKNADYWAEQIRSGRYVNLEQKQPNATFKKNIPQRCVRDN

>BAG12118.1 vitellogenin [Lethocerus deyrollei]

MRSSAILFLFAAVVASSSAESWSNATAWKTNTVYKYILRGRSMTGLHQVSNQYSGILLKAQVAIQLKTQNLLSLKVGKAEYAELHANLSKGWNTEIPDNKLHWQPMPLAGKPFELKLKNGTVDRMYVDKSVPTWEENWYKSIASQLQIDTAARNLQKSRINQLPAMHKPMGVYKTMEDTVTGECETVYDVSPLPDYLLQSKPELAPLPHLRADGRIIEIVKTRNFSECDQRVAFHFGITGPTNWEPAGNQMGPFMARSSVSRVIISGKINKYTIQSSVTTNKVILAPHLYNKQKGIVASRMNLTLESVQPAQGQPQSIPVPRTIKDLVYEYNAASEDSHKQGHEIGQDSSSSSSSSSSSESGESSSSSSSSSSSSSESSQEQQAQKPKHARNRREAVRPKQNRQAEDSSSSSSNSSSESSNSTSSDESSSSSSSSSSSSSSSSSSSSSSSSSSSSSESSSSSSSSTEEDYQPRPKLRIPPNSPLLPFFIGYQGNTIQAAHKINVVQQAQELAKEIALDVQTPNAITGEDTLTKFTVLIRALRTMSPEQIKQVAQQLYFPAHKASNHTVTDAKKYQAWAVYRDAVAQAGTGPALVVIQDWIKNEKVFHERAAELVATLQDSARHPTLEYMNTFFELVKSPQVMKQMYLNTSALISFTHLVRKAMVNNETLHHRYPVHAYGRLTRKNSTVVVLEKYIPYLAEKLRRAVEQEDSPKIQVYTQALGNIGHYKILSAFEDYLEGKVNVTDYQRLVMVTALHKLTVVYPKRALPVLYKIYQNIGETPEVRVAAIMLIMRTNPPAQILQRMAESTRFEHSIDVRSAIQSAIQSAAKLTGPKYYELAQNAKAAVHMLNPTQYGYHYSKHHMRSYIAEQQNLAYKHDISLIQSSDSIIPSSLFYNLKRKLGGYRTQVFKLAYMTSSVDDLLELIFDQFEDEEPEHKSKRPSPGPEDWTLQKIKEILDFDVDVQEQVEGNIQATLFGSKRFMAWDNHSIEALPYIVKEAAKSLKNGQPFNMTKWYNPLNIELAFPMATGLPFVYSYRTPTYFSIGGEVRAKTTPDLAKGDDDEIKIPDTVNATIQARVIYSTKTQATMGFVTPFNHQRYIAGLDRNIHVYLPISGKLDVDIENTKLWATLYPLRPNRYQKLLEYSTVAYTTRHDILDLSPPAQNDEAEEIHVRPPMRYSATVGQHSTGFAYDLKAETEKDVLELSTVYNALQRHDIVSALLYSSHEMSITNSNFSVAYNPQKTSAKAAKIVFTYDDDDDDESSAEQKHRSHNKHRGVNIAYPISTDSDSAESQEQYLDAAGEGIEDSLARMIDVSVQFEGDSKAEYVATAAYADSPVSNYSRWLLFLSTEPAQKTQQTKPFQLTLNVTTDFPDVPILNYKRALRADPTSQVYAHLNFGENAQHGAYVYLEGTLEQTKPRIQYVQRHPLAKLCEQQMEEGHNILPACRNITMRANFLDSYSFDVEYKDVPEYAKNLSYHAYAMIRHMFYPYVSENFIDPENEHGKLSVDVDFAANLKTVNVSIDTPLFSSEFRNVSVHPWVRPIVVSHPEYYAYDRFVYKAYRAQYFPVCAVDKSYATTFDNKTYPIRLGNCWHVMAYHTPDESPESSEEEDEDEAEFAVLVREVSSNKKELRVILGDDIIELQPSGQRPDVIVNGRKIKYAQESLTEVNDAEGETLLEVYALPAGGVRMYAPQHVLEILYDGERVKLHASNWYRDEIRGLCGTFDGEKVTDFLAPRNCILKNPSLFVASYSLPGHTCHHPAAEELRRKAQNAPCYEPEVILGEVVSERDLGKVRKQPKPQWSNSLERSDSKRACTHYRVKVLEVGKKMCFSLRAHIACGAGCQPASKLEKKVDFHCIENSAAAQHWADSIAKGHNPDFSKKQPNYRATVRLPENA

>AGV05363.1 vitellogenin [Nesidiocoris tenuis]

MMWTPILILALATFSAAEHGWKSGTQYVYNVRGRTMTGLHQVADQYVGIVMRAKLSVQPKSDSELVLKVNNAEYADVHANLTGGWNGYIPDSKLSYQQLPISSKPFELRMENGAVDKMMVDKSLPTWELNMLKAIASQLQVDTQAENLQKSRINSLPSKDQAYGVYKTMEDTVAGEVETIYDIPPLPQYIIQSRPELAPMPKLRADGQLIDIVKTRNFSNADQKMAYHYGFTGLTDWEPASNQMGNFLSRSSVSRVIISGSLDRYTIQSSVTTDKIVIAPHLYNNQKGIVASRMNLTLEAVNSASGSPGSVSNPRTVKNLVYEYNAATQQEGSQNHNAYKQYNSNEDSSSSSSSSSSSSSSSSSSSSNSASNSTDSSSSSSSSSSDSSSSSSEDSQNNSANSPSNKNQGAAKNRSRRSASPQSHGKKNQQGNASNNSGSSSSSSDSNESNSNSTSSSSSSSSSSSSSESNESMNAKLRSGNSSESSSSSSSSSSSSSSSSSSSSSSSDSDSSSSSSSSTSSSEEFWQPKPKLDMPPHSPFLPYFIGYQGNSIQASKQIDGVQAVQKLAKQIGAEVQEPNAIPGENTLTKFSIMARVIRTMSYEQLKKASEAIYYPYSKASPKSDSDAQHYQAWTAFRDAVAQAGTGPALLTINDWIQSHKISGQEAAECVSALQNAARLPTPQYMDAFFNMATSQECSKQWFLNTSAILSFSNLVRKAQVNNDTAHNRYPSHAFGRLSPKNAKAVAEKYIPYLQSQLRKAVSQGDSPKXQVYIRALGNTAHPKILSVYEPYLEGKEPVSDFQRLTMVASLDKMTKTYPKLARSVLFKIYQNAGDAPEIRVAAVMQLMKTNPPAQILQRMAEATNSDHSEQVNAAVKSAIESAALLHTPQQQELAQNARTAVNMLTPRQYGAQYSKNALRSYIVEQQHLAYQGQLSSIQEDDSILPSSMFLSLRKNLGGYQRQEYQAGYMTSSADDLLSLLSDQFSQSQQSRNSKKSAKHQASSGSSSSSHWSVDKVANMLNIEMDDADQVEGNVFISMLSGRKFFAFDNHTIEQLPKLAREAASKLREGQHFNFTKLYNQYSAKIGFPTATGLPFVFSFNVPSYAYVGGAIQARSHPDIGSGSHSQIQFPNTVNASADLEITYSAKAQGRLGFITPYNHKRYSAALHKNIHVHVPLRASVDIDVENNKVYAKMQPLEGHQKHKLFEYSTVAYTVKHDILDLYPALNGNNAEIIHVRSLRRNETTVGQDCSGFAFDIKLESEQKFLDWATPLNALRRHDAISALLYPTAEQSINYNNFSITYNPNKSSAKVARVTFAYDDQDESNDNSAESGHQSHNKHRGSQESSESGNAAASSSASPDSAQRQQQFLRKAGAGIQDSYAQMVDVSFQFDGEHKAQFVATAAYASSPVSEKSRFLLFLGMSPAKNGKDQQIAVQVTAKMPNVPLANYQKAQSADPTSYISAQVNFGEKASSGSKVTVEGKLRQSSERREYVRQHPMSALCEQQMQQGNYIQPACRNATISANMLDQYRFTVKYDHVSEALKNATYKAYAIARYFGNLYVSEDVISPNNKNGQLDIDVNIAQNLRSVNVSMDAPAISASFNNVPLNPYVAAAIVAHPEYNAAQRVAQDAYLSQQFATCAIDKNRATTFDNRTYPINLGRSWHVMAYSQPKSSYRFQLSLLFIQRRAVLQAFWFVKLDQTRRRLWLSFENDILSILPQSGGQNAAEVKFNGKKASFKPKVISLLSKMKMVTLSSKVYALPSGTVRLIGQKNGIEILFDGQRVKLQASNNYRGQMRGLCGTFDGQSADDFTSPKNCVLKNPYEFAASYAVPDSXLTSPAKELRQRAEQADCYKQTVMLGDVINENEAGRSSKKLWSKSSSNSNGRSQASTSPSSLRVKVIEHNGQTCFSLRPYVTCASRSQEANKIEKSVDFHCVSDAQAARRWVEQIKKGANPDFSQKGANHRATIRLPGKCNA

>AEL22916.1 vitellogenin [Nilaparvata lugens]

MNGLTLLLCAIAVAGVSASGSGPWNSNQQYRYHVQGRSLSAMHQSGSNQYVGMHLRAELEVEAKNENQAVFKISKAEYADVHQNLSGGWQQELRSNELQYKQLPLSQANQVFQVNYKQGAVRSLQVNRNTPTWELNMIKGFVSLFQVDVTGQNAIKSRRNIVPNGQQVSGSFKVMEDSVTGKCETHYDVDELPMRVVQQHPEIAPLAVKQQGQGQGQCHSRLIQVVKSRNFSNCDNPVTYHFGFTQESNFEPASNQMGNLVSRAAMGRIIIAKEPDSYTIHSSVTQNEIAISPFGYNQQKGVVGTLMNATLVSVSHASSGSPQSVQNPQKINDLVYEFNPASNSESNQRSSHYTRQQADNEDDSSSSSSSSDSSSSSSSSSSSSSSSSSSSEENNKNSKKNNKNWNNNKNWNNKNQKNNNNNNRNNHNYNDNNQDNSNENNNDDAYWRSQQKTKSRSRRSILRNYNNDNDDDNNQNRNQNRNNNNNNDSSEESNENQNNNNKHNNKNWNNNDNNKNWNNNDNNKNWNNNNKNRNNNDNDDSSSSSSSSSSSSSSSSSSSSSSSSSDLDSSEENWQQKPGMNDAPRTPFLPHFVGVRGNSIQADKQVDIVNEVQKVAMRIGAQAQRPSAIPGQNTLTSFTILTRMIQTMSAKQIQEVKQRLFIDRNNANGKSSADAKKLQSWEAFKHATANAGTGPALEAIKNWVEKGDVRNEKAAELVAVLPRTARLPTDQYIKTFFQFATSSNVQNQKYLNSTIILGFSEILRKAQVDSDTKHMRFGVHSFGHLTSKHGQSLHQEYMPYLEEKLKSAFEKGDSQKIIVYIQALGNTAHPRLLKTFEPYLEGKKSASRFQRLLMVASLYQMTRVHPTTARAVLYRIYKNPGEAAELRVAALHLLANANPSAAMLQRMAQQTHWEQSKEVISATQSFIKSAARMDQNPNSIELARNAQAAVDMLNPNEYGSSLSKNFLSSFVIDNIDKSYESQLSSIGSVDSIIPSSVFVNFMANDGGYKHQVFHHSAMFSSVNDLLELVNTQFKNNNNNNNNRRNNKSGSHDNEDNHNNRNSNNEWTAENVLKALNIQKDQAEQLEGNVFLTMLGGKRAFAINNHTIEKIPSIFKEAAQKLKHTSFNLTQFYSKNTMKVAFPTPMGLPFVYTSSVPTMVYVGGETKVNSHPDLANGNNNFVNIPQYINISADIEAVYSMQSNSKFGMVTPFNHHEYYASVEKNMQFYMAVQTEANIDLENNEVEFTVQPLNKEDKQNVFQYSTVLYTTKSNILNFNPALQEDGTERVHVGKAEQIEMKFGKESTGFAFEASYWSENGYGDFATLYNEVSKFDFQSAMTSPWAQGSLNSNNITVAFNPRQSTSQVAKFTFSYAENSDDNNNSHSGHDSNSSNNNNNNRADYSDAQPSSTAANSRSRQNEFLRKAAAGISGADAVVVDVSARFQGSHGQSNAQYVATVAMANSDASSNARMLFFASMNPANSDSKAQVCAAVASNFPNVPLMNFLDALKANPTSHISADIAFGAQCNAGGHIHADARLSQTQEFQEYAKSRPMAKKCFQLMEKGQALEYACQNATKVANMLNNYEVSVKYDRVSSVFKNVTYSIYSALAQAAYPYHSENMFSQNSNPSGKIDLNARFNYNLRYFNASINTPFFSANVKNVEVHHALRPLVIFHPSLNSLELMSYNENYDYPTCSVSKNSISTFDNKTYSADLEGWHVMFASTPKNYNDNSGRYSASNSQSNSFYKYKKVVVLAKNAGSQRKAVKMLLGENVIDINPSGSESSDNSPNANVQVNGNKVQIANNRMASFDDFDGETLVEISVTDNGEVQVQSSSHGIAVYHDGANFIIDADSYHRGEVRGLCGTYSGDKYTDFTTPNKCIMREARLFAATYALPGSSNSNVEQLKRQADQMTCFRRRHIFANVITSNDYDRSSSSSSSSNRNNNRNNNKNNRSNNNSSERLANPTKLIQDVKNNGDQVCISIRPVPKCQKGFSPAGSSEKEVDYVCMSHGKNAQFWINQIFQGGYVKLEQKQHNATFMKNIPQRCVRDN

>sp|O76823|O76823_BLAGE Vitellogenin precursor Blattella germanica

MTWNALLCCLLVSAASAITPGWLPINSQLDYHVHGRTFSSLFQVANQYTGILYKARLSLDRNEDQLITGKVTEAQFSPVNTQFSSGWDESVPDEKLHWDVVPMSQQPFQIELNSRGEVRKLRVNKFVELWEINMIKAIISQLQVVVDEDKKVYRVFESTVTGRCEALYEVDHLYPTTYLNPWQWTQQHDTKLRIMKTHQFTNCRHNSAYKLHFNAFEYFHLKQHKPETFLSNSAVSRVIADGDNLKNFTFYSGETIHKIVLNPEIYNKQKGMLVSHINVTVERKGRELTVIDYELRNVGDLSYSTSLVKAHSMRNSASMDLSSSSMSSSSSSSSSSSSSSSSSSSSSSSEEHHSHNQKLSKKRQVPLPRPLFEANFDASSGLTTEQPVTFRPRRQLFQGQDMSEEETEQNPEIIPANLLPTYNLIHNTKQVDVDPVGVAVRLSKDIAADLQGEPRVGEDRHILPRFTILVRLLKQLKVSQIMEAARKLYKLENDHPNYMNWDTWRVYRDAVSQAGTWSALNSIQQFISSEMVEPKEASHLITVLPAAVSDKNKAYLHFLFEMTKDPVFKNMTYVNTSLVLAFSEVIHQVEMHQVRDLKIKSVYIPYLVQEFDDAVKENNSIKIQLYTHALGVTGNTHILHYLRPYIIQLKTITHHQRLFMVQSLERVVEHNPRKVIDLLLSLYLDQNEHADIRVEALFLLMKADPSIHVLKMVAELTHTESNNQVLSASQSAIKSAANVEGDIYSEMRRKAKAVEHLLSTRNMDVSYSKSYLYGYKSKKINYDSLYNLNYIGSEDSIYPKSMLLNIFTNNLGRINTHVQKGYMVSSMTDLWEAFHTIYKKDNGSPTDPKTLVKFVEGNLKYFNMGVQKFWAFDNTTFSNASAVIQEFLKTYKKPTNFNHTKLSSSSSITLTLPCAMGLPAYFKMNSPSLWKYNGEFSIQTDAKTDVPMSLENFMNITGSINLMFSQMYHAQLAFSTAFDNKEYISGLDRKVEVHVPVKFQINLDFKNHNGFIRIIPLFTDRDYDVLQWQTIPYTTIHNVPDFETVYMDQLFKLIHVRKTAHFEKKMGENTGIVFKVKYDTDQEFLDTKWFLDEFKVLQLFTGLNYDVPTKDIFYNNLTVYYDHEDTKNHAVSFTVTKEQSKFYETLNPVVQQNLKLSSGKKQKHRNVKSHRIRREYTEDENPAIPKDKQPNSHPRRQEYLSKSMALTGDATAVVLDMTLKFEGPAESYFTTTVSHATSLVNGSSNYLLFYDQHYYEEKKRNQFCLSWSVYKPQVPIMNIYSAFEFDPNSKVHAIMNIGKECENGGSAVANIDMLRLSEHLDYVKNLTVSKLCDHEMRTKRDHVLPACRNSTERASDLNRVHVDINYNLKQHETFKRRVYKVYDFVRTHLYPHVSEDVIVDNPAQFISANFTLKDNTRAFNVSIETPVLSVNATSVRLQSWQSEMLRMNPRTSFAKRFAKWALPLYYKPTCVVDSSYINTFDNFTYSAHHIVQNDAFYTILDIPQKFNMEYFKVAFKPTSPVPNMQREVLVFLRNAKIELKPNQGMPEVYVEGKRVDYNHHHSTDLNVSQDRIGYVYALPTKAAHIVFPSYEIEMFYDGSRIMIQASNMYRNFTKGLCGNMDGEFVNDVLTPWGCYAKDMALFVASYADNSNSEVRKIKATQNEQTCVPQFHQPLVSHQMRLSQVIKLADTSSSSESSSSSESHENNSSPSSESQVNKSKRQPNSRPRSSSSSSSSSSSESNESVLAKKIINNQIGPKPTLIPSQSPMTSDDKCMTQQPRHTYYENQFCVSEKPLDTCMPLICHATESYTIDVNFYCVPLGPAANHYMKLVKKGILPDLSRNRNGKRVVLPVEIPIQCEPVLN

>sp|Q9BPS0.1|VIT2_PERAM RecName: Full=Vitellogenin-2; Short=Vg-2; Flags: Precursor Periplaneta americana

MMWKTLLCCLLAVSAAALDGWEPGKRYEYHVRGRTLTALHEVANQYSGFRFKGKLVIEPHTPSVLRGQLKDTYHMTVHRMLPDGWDQKFEERESNWERVGMKNKPFEVHVGNEFQFNKLIVTEDTPVWETNMIKGVLSQIQVNLKEVGPDPRDEQEDRLRKIFKVHESSVTGRCEVLYDITPITKFNMLPQPLVEIEEENVNVLQVMKTQNFTDCKKLPSYVHGFYNFHNVFPAQNKAGFMSRSQQTRTIVSRNKETGRFTIRSSVTFHEVVLKPELFNSQQGISVSRMNVTLEEIKSQQHIPPPRPPKDVGDLVYRYSAETGEPSQRDSAYALESNSDSSSSSSSSSSEENAANSRHRSSSSSSSSRSSEEMRDSKKHPRASTTESQPRNSRSRRSLQNSKRSINMYNDSSSSSSSSSSEEYLLPRPHIENAPNIPFMPYFVGNQGSKIGEVDPEKIVLLARTISSELQEPDTMVKKNILSRFSILTNLVRAASFSQLEEATKRLYYRVERADNGDESKLDAWKAYRDSVAQAGTPAALKMVHTWIRKEYIKDEEAAKVVAVIPHAADTPTDNYIAYFFEMVKDPVVHGEKYLNSSAVLAFSKLLRLAAVDSEAVRRYPVHVFGRMVPKNFSARVKEYIEYFANKLKNAVKDKDSHKIQVYTRALGNTGHADIIRHFEPYLVGRESVSTHERVTMVFCLDEFVKTQPSVAQYILLRLFENVGETQEIRVAALYLLMKTDVSAELFQRLAEYTKFDKNHQVVSAVQSAIRSAAKVEGPYKKETAKNAQAAVKILSSKPYDDSYSKSFILNNYRREIDVGYSRLYNQIGSRDSFMPKSVFYKLVNIIDGDRDDQAKFGGAVSSVRDVIDFIRQQFKKDDSQDELENSKYAEDDDIWDLREIANLLEMEEENVDPLEGNVHYDYFGAQRFFTLNKTSFEFREELKKYFKKPQITNINKLYNRMELKVGYPNVMGVPFFFTFKRPTLVKLTAKTFIMPLKPCDHGKPHKFPRIFNVTSDVSFVYSFDMHSHMGVVAPFNKKEYVTGIQRKHMIQIPLNVSVHVNLDKNKVAADFKPYYEDNFKVAEARGIPFTTVHDIKSLVPYVEAEHTSYIRVRPSKAYEGNFGKSVGMVYHYNFETDQQFFDYKWFSSNYFLHYPNVAFYYGWEAQPVFYYDFKLYLDSHNSPAKTVQLKASYDNRYTQPEEEEETRQHSKIRRPRSASRKHRRSRHEERAPLENLEVSDTETQREELYDIVLPAVRAGRLYYASVSVAFKGEENVYSKYEVEGALASSQVNEHISTMLRAHSNDAERKHQYAHVRVNVTMPQVPVIDYRKALEFDPTSKIQCEVHFGDTPEKKSKVYFQGKFERTDERKKFVAESDMAQLCSAQQNNKNYLLPACRNVTEEASKLDKYFFKVKYENLSEKCRNRTYKAYSYLRHYFFPYITENVYPDERKTDSVEVQVQFNEEINAVNVSVKAPILNVEFTDVRVYNKYARALFSLNPRYPLLSQVAKTAFPQYYEPTCVVDYSKVNTFDNRTYEHDMLNDWVEVMFHKPRSKIYKQVSVSAKQAHSKMVLKVLRGDEVKFEMKQPRDSSSSPELKMNDKVIEYERSPAFIHYKHELIAVAYALPSKALHLDLLNDSLVFVYDGERVMLHAGNHYRNQVRGLCGTFDGEPSTDFKAPQNCHVRDVEDLILAYTLVRDLDRSRLRDENICVREDVQLVNLTNHRHAEKSGIRPYDIDDDSSSSSSSSSSSSSSSSSSKSNSTSSSSSESNESALPRGENKLHRAQQPSRNCHMHLHRIVTHNGKQCISKLALTECAPLCREESHTTKTEAFVCFPPGPTADHYTKLVRKGVSPDFSRKTDIVNLRVTIPSRCVSKI

>BAB19327.1 vitellogenin [Rhyparobia maderae]

MWYTLLCCLVVGGVAGVNQQPRVLPIPGYPRGWIPVQRRYTYNVEGRTLSAIHEVSNKFTGIFLRGQLILERPEPTLIRGQVREAKYAQVNQDFSNGWKQNIPDSQLKWKDLPLRQDTFDAHINDTSGEVEILYVNSQLQLWEVNVIKGLLSQIQLTTQPSFKPVYRVKESIITGRCHTLYDFSPLLKTEMKLWNYLDNDNLQVTRTQNISHCNSHLFHLKFSGFEHFTDRMNNGGFISNNVVTRMVVDSVENNLTVIASNTVHKVILSPEYYNTQHAMTVSFMNVLSRKSNQLSLHPVSDPRNVGDLVYLETLYERHDQQYLDSSYASASSSSSSSRSRSSSSESSEETDSINIRNRENKQRSPRAISQKKRMALMQELGLTTPLPYPRRLRSISLSAESSSSSSSRSSPEISRERNPRYIKDEENTFLPLTHVLRSDVDPVKAVVQLANDIGHDLIDPDSLPDKDTITKFIIMVRVLRNLQLSEILDIAQQLQVKLDSQMVRKDSPQWEAWKSFRDAVSQTGTHAAVHSIIIFLSRRYISQSEAQDLFNVLPAAVQHHDMQYINNMFDLIKDPVVQQDRHVNETVVIAFSNAYRFIHARLKRPYISPYFIKYLFQEFENAYRRQNTTQMQVYVHTLGNTGDVRIIPYLEPYLLRQIHLSAFQRAHMFKALERVVDANPHLLTRFFLKFLLDQTDHPDVRVQAVFLLMRSDPSVAVLRTMAELTHSEPVNQVVSAIQAAIRTAARLRGTRFYNLAFKAQTVVNLLSDKNLDVSYSKNYMLDQEAREYNLDFQLFYEQIGSQDNLLPKSALLDIFSYVGGAKSDHQTGYTVSSIDKVLNDIQLQFKNFTQQGWTQQQMRDDLTKLVEGNIQYQVLGVQRFWPFDQDSIKSIPNVIQKFVKDYREVKSFNLTKFFTTSTGIYGFPTVMGFPGVYTLHTPSLWKADGELKVTTVPDLEQNPHYLPGIVDVQLRVRPLYAAKLQSKLSVITPFNDMRYTAGVNRHFQLHVPFQVKIHAEMNYNNMNDDKNNNLYAIRANVKQYDSDKDHRVLYMSSIPFTTIHDIRSLNPDSKDDDFQILHVRPMKKYNRDYGQDVGQAVKLQYETEDDYVDLKLSNKNIWLSNALSPLPGWMTSQIIYRKLGVTYATRQCTNNVIELSAVLANDQQNNQYPNTQNDDGHSARKHKARRTRSARKDDRQSSGERSDSNPAIPSDTKPDSDARRQQYLRAAREQVRNNASSYVLDLGVNFKGQNPAYIVFTGAYAKSLVNGNSNHLVFYNQQFLKPEDNKQVCLSANIMKPQMPLNNYDDALQSDPTSQVRMILNAGNKCQEGSGQATVEESFKELRSMKNSSKTGHWLGNVRMTWTSTAIISSEHCQNVTYRADDLKDYTFRAIYDNKLPDFVKERLYQAYALLRNRLHRHVSEDPFKIKANSGQLDLSVQLNNVSKVFNLTLESALGESRFINVPVHDWAGNMLSVNPRTSIAERLAQYELPLYNNPTCALDNSAINTFDNLTIYNRFENKEYTLMQVKDQDTTLRVRKIDVRMKVQDSNKDVKIITEKATVQLKHNNDKPDVYFQDRKISYTNNEATPLMANDHLFGYVYGLPKKSVMVVLSQPNVAFVYENQRFLLQASNIYRNKTRGLCGNMDGEEITDLLTPNECYELDYKKFFEAYTNGNQHYMDKTCIRYFPIDDMNYFPKQQRQRNPAYPSDLSDVLSKSISGTSSQTSSASSNENKQNRHSHSTSSSHSSHSHSHSHSKSHSHSSSSQSHSRPKHSRPEQSRSSSSASRSRHSASRASRASSQNSDSESRERTTTQNPMDNSIRPNIHRKQNMVVITRVVRRDTDVCFSAEPLKTCIDNSRAADTRIQQQQFICLPDSPAFEHYLKLIKKGINPDFTRKKNFVQLEVKIPTKCIKSQ

>BAD72597.1 vitellogenin-2 [Rhyparobia maderae]

MWYTLLCCLVVGGVAGVNQQPRVLPIPGYPRGWIPVQRRYTYNVEGRTLSAIHEVSNKFTGIFLRGQLILERPEPTLIRGQVREAKYAQVNQDFSNGWKQNIPDSQLKWKDLPLRQDTFDAHINDTSGEVEILYVNSQLQLWEVNVIKGLLSQIQLTTQPSFKPVYRVKESIITGRCHTLYDFSPLLKTEMKLWNYLDNDNLQVTRTQNISHCNSHLFHLKFSGFEHFTDRMNNGGFISNNVVTRMVVDSVENNLTVIASNTVHKVILSPEYYNTQHAMTVSFMNVTLEKKSNQLSLHPVSDPRNVGDLVYLETLYERHDQQYLDSSYASASSSSSSSRSRSSSSESSEETDSINIRNRENKQRSPRAISQKKRMALMQELGLTTPLPYPRRLRSISLSAESSSSSSSRSSPEISRERNPRYIKDEENTFLPLTHVLRSDVDPVKAVVQLANDIGHDLIDPDSLPDKDTITKFIIMVRVLRNLQLSEILDIAQQLQVKLDSQMVRKDSPQWEAWKSFRDAVSQTGTHAAVHSIIIFLSRRYISQSEAQDLFNVLPAAVQHHDMQYINNMFDLIKDPVVQQDRHVNETVVIAFSNAYRFIHARLKRPYISPYFIKYLFQEFENAYRRQNTTQMQVYVHALGNTGDVRIIPYLEPYLLRQIHLSAFQRAHMFKALERVVDANPHLLTRFFLKFLLDQTDHPDVRVQAVFLLMRSDPSVAVLRTMAELTHSEPVNQVVSAIQAAIRTAARLRGTRFYNLAFKAQTVVNLLSDKNLDVSYSKNYMLDQEAREYNLDFQLFYEQIGSQDNLLPKSALLDIFSYVGGAKSDHQTGYTVSSIDKVLNDIQLQFKNFTQQGWTQQQMRDDLTKLVEGNIQYQVLGVQRFWPFDQDSIKSIPNVIQKFVKDYREVKSFNLTKFFTTSTGIYGFPTVMGFPGVYTLHTPSLWKADGELKVTTVPDLEQNPHYLPGIVDVQLRVRPLYAAKLQSKLSVITPFNDMRYTAGVNRHFHSRSLPVKIHAEMNYNNMNDDKNNNLYAIRANVKQYDSDKDHRVLYMSSIPFTTIHDIRSLNPDSKDDDFQILHVRPMKKYNRDYGQDVGQAVKLQYETEDDYVDLKLSNKNMWLSNALSPLPGWMTSQIIYRKLDVTYATRQCTNNVIELSAVLANDQQNNQYPNTQNDDGHSARKHKARRTRSARKDDRQSSGERSDSNPAIPSDTKPDSDARRQQYLRAAREQVRNNASSYVLDLGVNFKGQNPAYIVFTGAYAKSLVNGNSNHLVFYNQQFLKPEDNKQVCLSANIMKPQMPLNNYDDALQSDPTSQVRMILNAGNKCQEGSGQATVDGKLQRTKEYEKFIKDWALARECQNDMDKYRNYLLRDCQNVTYRADDLKDYTFRAIYDNKLPDFVKERLYQAYALLRNRLHRHVSEDPFKIKANSGQLDLSVQLNNVSKVFNLTLESALGESRFINVPVHDWAGNMLSVNPRTSIAERLAQYELPLYNNPTCALDNSAINTFDNLTIYNRFENKEYTLMQVKDQDTTLRVRKIDVRMKVQDSNKDVKIITEKATVQLKHNNDKPDVYFQDRKISYTNNEATPLMANDHLFGYVYGLPKKSVMVVLSQPNVAFVYENQRFLLQASNIYRNKTRGLCGNMDGEEITDLLTPNECYELDYKKFFEAYTNGNQHYMDKTCIRYFPIDDMNYFPKQQRQRNPAYPSDLSDVLSKSISGTSSQTSSASSMKISKPSFSLNFVIHSSHSHSIPILIPSHSSSSQSHSRPKHSRPEQSRSSSSASRSRHSASRASRASSQNSDSESRERTTTQNPMDNSIRPNIHRKQNMVVITRVVRRDTDVCFSAEPLKTCIDNSRAADTRIQQQQFICLPDSPASEHYLKLIKKGINPDFTRKKNFVQLEVKIPTKCIKSH

>ADB94560.1 vitellogenin [Actias selene]

MKLLVLAVVIAAVSSYHGDNNPESNPSPWQVGKAYRYNVKSHTLARLEEGPNSGTAFTANFIIRVKSHGRLQARLENPQHAQINEQLPYERDLPENLKYQPIQNLDKPFEISFEGGRITSLNLPSTISLQHENLLKGLISTLQVDLSTYRNIHGSQDNYDQEQQQGLFRKMETDVTGDCEILYTVSPVASEWRRELPKFASEEDPIEITKSKNYGHCHHRVAYHFGIPEGAEWTGTAHNPEEDQFIRRATVSRILAGKLGPIYKAETTSTVQVHPHLYGKQKAEVHSHVHIELESVEQDSEAEWEKPEGSRTVKNLLYAMSTKQIATHDSSSSSSSESHEHAINEEPKQRSRRSMRASKVGAIQNYMSQQKKHRDDSSSSSSSSSSSDSSSAYINDEMPGLNDPVYAALYMSPQTHTDKKQNSVNAQKLLQDIAQQLQNPNNMPKSDFLSKFNILVRLIASMSTEQLSQTSRTIEAGKSSNNNIKKDMWMVYRDAVTQAGTLPAFQQIKSWINSKKIQDEEAAQVVASLSSTLRYPTKEVMIQFFKLARSPEVKDQLYLNTTALIAATRFINMGQVNNYTAHNFYPTHMYGRLARKHDNFVLEQILPPLSEDLKNAIQQQDSVKAQVYVKAIGNLGHPEILKVFAPYLEGQIKVSTYLRAQMVSNLIVLSNQRNKQARAVLYSILRNTAEPYEVRVAAIHNIFISHPTGAMMQAMAEMTHDDPSVHVRSALKSAIECAANLRGPHSWELSRSAQTAQWMLEKNNFGYQYSFKLFNDGYDMENDLEIFSALSHIGSDDSLIPKFLKYSVKSKNTGWNKIQASVSSYKHFAEILKESMFYQQKSKSDHRYSSSKISELLNIKRDQSDPLEASFYVDLVNQQRYFTFSEEDLRQLPNDISEYFKKLEKGVEQHYTKILNQAQVSVMFPVAMGMPFIYKYKEPTLIHIQGKAKGEFTRPTKEQPQYSAQMAKEVQFTYARNIDGDVGFMDTISNQHVSVGVVSKLQLNVPVKLDIQVKPKQFKIRAEPLHPEQDSTIVHYSVWPYSAVQKKDSIVPISLDPTSKVVERQRKILSVDTKFGQATSTVFQFQGYSYSTDFKNFGTVFNSPDFITNIASIFSQQDIAMTHFNLRYLAKQSQNKAVTLRAVYDDYYNQKESGELGPAADQADFSPNSEARRQAIAKRVSAGINTAKAQVVDFSATFEGSHKADYVLTAAISESPVDPKVQYALFAGKNSAQHGKSQFNAVGTVKLPRSNALNFLQVLDNDLKTTFEADIKFNHNANVHLQAEAERSKRYTEELQNHPLAKQCAQDIARNNQYTHTCHRMLVLAHAPDYMKLSVNYKDISNAYKNYTYHAYMFAKHLGFWYADVNPIKTSPEGKVEVELEASYFDQTLNASMLSKYGYVRMENLPIPRAAPAALAIYQPFQPQERVANFYTSHQYQPYCSVDGSKIRTFSNRTYDYTLTSSWHVVMQDEPQEHGIGAEVVVLARKPKANQQEVYISYKSETGKDLEIEIQPAPEGSKQPRVNVKTNAKKVSEGELTIYWNDVEQKPLLEYYYQQDGALMLNIEEYKFRTVYDGQRLVVLASENRQSARGICGSMSGEPRDDYLTPEGLVDKPEHYAASYALNDENSDPRTQELKAKAKQEAYQPKNKYTTVLRSDPQWQQQMSASSSSEEDWGSETVYRSRSYDKQRGPCAVKQQVQYYENHGEICITTEQLPACQSHCHGDEYRIQAAQVSCRPKLDHQYRAYRDQIKQGQNPTVTGVPKVKQFKVPTACKA

>BAE47146.1 vitellogenin [Bombyx mandarina]

MKLFVLAAIIAAVSSDRFSSQSQSTGGQTYPSPWQVGKQYRYEVTSRTLAHLQEGPSSGSAFKAQFTIRVKSPGRLQAKLENPQHGNFNEQLPDPRELPVDLKYQPTPNIDKVFEIEIDGGRIVSFDFPTSVPVPQENLIKGLISALQLDTSAHRVIHDSQNNYDREQQQGLFRKMETDVTGDCETLYTVSPVASEWRRELPKFANEQDPVEVTKSTNYGHCHHRVAYHFGVPVGAEWTGTAHKTQEQQLIGRATYSRILTGKEGPIYKAETTSTVHVHPHLYGKQKAEVYSHVHMELISVDQDSGAEWPRAEAMRPAQSILYSLSTKQMTKHYESSSSSVFFGISTNSISPEQHEHPHQTNQRSRRSYTRSKLVTVHKVLKKRSSESSSGSSSSSADSSSAYINDDIPDIDEPAYAALYMSPQPHADKKQNAMNAQKILQDIAQQLQNPNNMPKSDFLSKFNILVRLIASMSTEQLSQTSRSIETAKTSNNIIKSDMWMIFRDGVTQAGTLPAFKQIQFWMENTKIREEEAAQVVAALLRTLRYPTKQIMTQFFNFARSPAVTDQMFLNSSALMAATKLINLGQVNNYTAHSYYPTHMYGRLTHKHDAFVLEEILPTLAADLKASVEYKDSTKAQVYIQAIGNLGHREILKVFAPYLEGKVEISTYLRTHIVKNLKTLAKLRDRHVRAVLFSILRNTAEPYPVRVAAIQSIFISHPTGEMMQAMAEMTHNDPSVEVRAVLKSAILSAAELQHPRNFDLSRTAQAARYLVTNEEFGYQHSFKFIDDSYDEDNDIGTFVISHIGSEDSLLPKDFKIVTNSKGGAWERNTIEASFSSAERFLDYLRDSVFAPHPKFDRAHKYSAEKIAKLLNIKNDEEEPLEASFYVDFMNNQRLFSFSESDLQQLSQYISEYMKKVESGAEKHYTKVYNQDQVSIMFPVASGMPFIFKYKEPAVIHFQSKLKGKFSFPSKDNKYYEANMNKDVQFTYARNIDGNVGFMDTLSNQYSSVGVVNKLQFNIPFKFGIEIKSGLIKLRVEPLHPDQDQTLVHYSVWPYSASQKKDSLVAISQDPATKIVERRSKVFSVDSKYGQSTHAVIYAQGYTYSSDWRNFGAKFTSRDYFTNLASLLTQEDIALTHFNLKHLCKQSQSKALTITAYYDEYYNQQNSGKLTDATDRNDLSPNSETRRAEMVKLVSAGINKARVRVVDLSASFEGSQDQHYVFTGTWGDSPVDSKVQGMLFAGTKQGNQQINAVFTTTKPEIHSLSFSKALQSDLRAPFGMHFKYGQSGEIRVSGSLDRTKKYTTELENHPLAKQCSQQTTLNNFYQDSCHKAIVMAHAPDHVGFSVSFQDMSPQYRNFSYHTYRLYEYLGYWYTEANPLKLTQHGKMDFNIDFSYFDRTYTVDIASPSGEVRMRDMPIVTMAPGALSFYQPLKAYELVANYFTGHQYQPYCSIDGTRIHTFSNRSYEYPLSRSWHVVMQDESTQRGNWHELAILSRRQQRDQQEIYISYKSESGKDLEIEIQPASGDSAYQVKVTTNTKKITDDDLTMYWDDVKEQPFLQYHTHKDGVLVINIEDDRIRAIYDGQRFVVFTQDYRNSTRGICGRMSGEQRDDYLTPEGLVDKPELYAAAYSLNEENSDPKTQELKALATQQAYYPEYKYTSILRSDPAWQEESQSSGEDQWQSETVYKSRSYDKHKGACEVRQQVQFYENHGDICITTSRVPSCQSHCRAGDYKIQHVQVTCKSKLDHDFRMYKEQIKKGQNPEVSGIPSVKQFKVPVTCQP

>sp|Q27309.1|VIT_BOMMO RecName: Full=Vitellogenin; Contains: RecName: Full=Vitellin light chain; Short=VL; Contains: RecName: Full=Vitellin light chain rare isoform; Contains: RecName: Full=Vitellin heavy chain rare isoform; Contains: RecName: Full=Vitellin heavy chain; Short=VH; Flags: Precursor Bombyx mori

MKLFVLAAIIAAVSSDRFSSQSQSTGGQTYPSPWQVGKQYRYEVTSRTLAHLQEGPSSGSAFKAQFTIRVKSPGRLQAKLENPQHGNFNEQLPDPRELPVDLKYQPTPNIDKVFEIEIDGGRIVSLDFPTSVPVPQENLIKGLISALQLDTSAHRVIHDSQNNYDREQQQGLFRKMETDVTGDCETLYTVSPVASEWRRELPKFANEQDPVEVTKSTNYGHCHHRVAYHFGVPVGAEWTGTAHKTQEQQLIGRATYSRILTGKEGPIYKAETTSTVHVHPHLYGKQKAEVYSHVHMELISVDQDSGAEWPRAGAMRPAQSILYSLSTKQMTKHYESSSSSSSSESHEFNFPEQHEHPHQSNQRSRRSYMRSKLVTVHKVLKKRNSESSSGSSSSSADSSSTYINDDIPDIDEPAYAALYMSPQPHADKKQNAMNAQKILQDIAQQLQNPNNMPKSDFLSKFNILVRLIASMSTEQLSQTSRSIETAKTSNNIIKSDMWMIFRDGVTQAGTLPAFKQIQSWIENKKIQEEEAAQVVVALPRTLRYPTKQIMTQFFNFARSPAVKDQMFLNSSALMAATKLINLGQVNNYTAHSYYPTHMYGRLTHKHDAFVLEEILPTLAADLKATVEYKDSTKAQVYIQAIGNLGHREILKVFAPYLEGKVEISTYLRTHIVKNLKTLAKLRDRHVRAVLFSILRNTAEPYPVRVAAIQSIFISHPTGEMMQAMAEMTHNDPSVEVRAVLKSAILSAAELQHPRNFYLSRTAQAARYLVTNEEFGYQHSFKFIDDSYDEDNDIGTFVISHIGSEDSLLPKDFKIVTNSKGGAWERNTIEASFSSAERFLDYLRDSVFAPHPKFDRAHKYSAEKIAKLLNIKNDEEEPLEASFYVDFMNNQRLFSFSESDLQQLSQYISEYMKKVESGAEKHYTKVYNQDQVSIMFPVASGMPFIFKYKEPAVIHFQSKLKGKFSFPSKDNKYYEANMIKDVQFTYAINIDGNVGFMDTLSNQYSSVGVVNKLQFNIPFKFGIEIKSGLIKFRVEPLHPDQDQTLVHYSVWPYSASQKKDSLVAISQDPATKIVERRSKVFSVDSKYGQSTHAVIYAQGYTYSSDWRNFGAKFTSRDYFTNLASLLTQEDIALTHFNLKHLCKQSQSKALTITAYYDEYYNQQNSGILTDATDRNDLSPNSETRRAEMVKLVSAGINKARVRVVDLSASFEGSQDQNYVLTGTWGDSPVDSKVQGMLFAGTKSATQGNQQINAVFATTKPEIHSLSFSKPLQSDLRAPFGMHFKYGQSGEIRVSGSFDRTKKYTTELENHPLAKQCSQQTTLNNFYQDSCHKAIVMAHAPDHVEFSVSFQDMSPQYRNFSYHTYRLYEYLGYWYTEANPLKLTQNGKMDFKIDFSYFDRTYTVDIASPSGEARMRDMPIATMAPGALSFYQPLKAYELVANYFTGHQYQPYCSIDGTRIHTFSNRSYEYPLSRSWHVVMQDESTQRGNWHELAILSRRQQRDQQEIYISYKSESGQDLEIEIQPASGDSAYQVKVTTNTKKITDDDLTMYWDDVKEQPFLQYHTHKDGVLVINIEDDRIRAIYDGQRFVVFTQDYRNSTRGICGRMSGEQRDDYLTPEGLVDKPELYAAAYSLNEENSDPKTQELKALATQQAYYPEYKYTSILRSDPTWQEESQSCGEDQWQSETVYKSRSYDKHKGACEVRQQVQFYENHGDICITTSRVPSCQSHCRAGDYKIQHVQVTCKSKLDHDFRMYKEQIKKGQNPEVSGIPSVKQFKVPVTCQP

>BAB32641.1 vitellogenin [Samia ricini]

MKLFVLAVLIAAVSSNHGGTKQQSNETPWEAGKVYRYEVKSHTLARLEEGPNSGSAFKARFVIRVKPNGRMQAKLENPQHAQIHEQLPYERVLPDDLKYQPVQNLDKSFEISYDGGRINTLSLPSSFSLPHENLLKGIISTLQADLSTHRTINGSQDNYDKEQKQGLFRKMETDITGDCETLYTVSPVASEWRRELPKFASEEDPIEVTKSKNLGHCHHRVDYHYGVPSDAEWTGTAHKPEEDQFLRRASVSRILGGKQGPIYKAETTSTVQVNPHMYGKQKAEVHSHVHLELESAEQDSEPEWEKPEGNRHIKNLLYSMSTKQIAKHDSSSSSSSSSSESYELRHIDMEPKQRIRRSIKVSKVGAVQNFINEQKKRSHDSSSSSSSSSSSSDSSSAYVNDEMPSSNEPAYAALYMSPQTHTDKKQNSMNAQKLLQEIAQQIQNPNNMPKADFLSKFNILVHLIASMTSEQLSQTSRTIEAAKSSNNNIKADIWMVYRDAVTQAGTLPAFQQIKTWIQSKKIQGEEAAQVVASLSTTLRYPTKDAMLQFFKLAQSPEVKEQQFLNTTAILAATRFINMGQVNNNSAHNFYPTHMYGRFARKHDNFVLKEVLPELAEALKNAIQRQDSVKSQVYIKAIGNLGHPEILQVYAPYLEGDIKVPTYLRVLMVSNLNVLCGQKNRQARAVLYSIVRNTAEPYEVRVAAIHNIFISHPTGAMMQAMAEMTHDDPSVHVRAALKSGIESAAELTGPRSWELSRSAQAAKWMLTKEKFGLQYSLKQFDDGYDMEDDLEIFSALSHIGSDDSLVPKYLKYFVKTKNAGWNKVQASVSSYKRFVEILKEGMFYQQKSKSQHKYPSTKISELLNIKRDQKDPLEASFYIDLVNHQRYFTFSEEDIKQLPHDISEYFKKLEKGVEQHYTKIINQAQVSVMFPVAMGVPFIYKYKEPTLIHIQGKAKGEFIKPSKERPQYSAKMTKEVQFTYARNIDGDVGFMDTISNQHVSVGVVSKLQLNVPVKLDIQVKPKEIKVKAEPLHPEQDNTVVHYSVWPYSTIQKRDSLVPVSLDPNTKVIERQKKSLSVDTKFGHATSTIFHFQGYSYSTDYINFGTIFKSPDFMANMAAAVSQQDLAMTHFNLRYLGKQSQNKAVTFTAAYNEYYNQRESGELGSATDQEDTSPNSETRRQAIAKRVSAGIKNAKATVADFSASFEGSHKADYVLTAAISKSPVDPKVQYAFFAGKNSAQQGKNQVNGVATIKFPNTNALNFLQVLDNDLKTTLQVDIKFNHNGNINMEGEAERSKEYTEELQKHPLAKQCAQDIARNNQYTHTCHKMIVQAHAPDRFKLTTNYKELSNTYKNYTYHAYKLAKYIGFWYTEVNPLKTVPEGKVEIEVAASYLDNTLDALMGSKYGYVRVENMPIPRSAAAAVAVYQPFQPQERVANYYTSYQYQPYCSVDGNMIKTFSNRSYEYTLTSSWHVVMQDEPQENGIGQEVVILARKPKENQQEIYISYKSETGKDLEIEIQPAPEGSKQPQVNVKTDSNKVSEGDFTIYWNDVEEKPLLEYYTQQDGVLMLYIEEDKLRAMYDGQRLVVLASENRESARGICGSMSGEPRDDYLTPEGLVDKPEYYAASYALNDENSDPKTQELKTKAKQQAYQPQNKYTSVLRSDSQWQQEMTATLSSEEDWGSETIYRSRSYDKQKGPCAVKQQVQYYENHGEICITTTQLPACQSHCRGEEYKIQAAQVTCRPKLDQQFRSHRDHIKQGQNPVVSGVPKVKQFKVPTSCNA

>BAD91195.1 vitellogenin [Saturnia japonica]

MKLLVLAVVIAAVSSLHGDNNPEYNLSPWQVGKVYRYDVKSHTLARLEEGTNSGSAFKAYFIIRVKTPGRLQARLENPQHAQIHEQLPYERDLPYNLNYQPVQKLDNPFEISFEGGRINTLSLPSSMSLPHENLLKGLISTLQLDLSTHRNIQGSQNRYDQEQKQGLFRKMETDVTGDCETLYTVSPVASEWRRELPKFASEEDPIEITKSKNYGHCHHRVAYHFGMPEGAEWTGTAHNPEEDQFIRRAAVSRILVGKQGPIYKAETTSTVQVNPHLFGKQNAEVYSHVHIELESVEQDSQPEWETNENNRLIKNLLYSMSTKQIATHDSSSSSSSESYDHSYINEEPKQRLRHSMKVSKVGAVQYYINQQKKHRYDRSSSSSSSSSSSDSSSAYINDEMPSLNDPAYAALHMSPQTHTDKKQNSVNAQKLLQDMAQQLQNPNNMPKSDFLSKFNVLVRLIAAMTTEQLTQTSRTIEAAKSSNNNIKADMWMVYRDAVTQAGTLPAFQQIKNWINSKKIQGEEAAQVVASMPLTLRYPTKEVMIQFFKLARSPEVKDQLYLNTTALIAATKFINMGQVNNDTAHNFYPTHMYGRLARKYDNFVLEQILPPLAEELKNAIQSQDSVKAQVYVKSIGNLGHPEILRVFAPYLEGQIKVSTYLRVQIISNLYALTSQRNRQARAVLFSILRNTAEPYEVRVAAIHNIFNSHPTVAMMQAMAEMTYDDPSVHVRAALKSGIESAANLRSPHSWDLSRSAQMAKWMLQKENYGYQYSFKLFSDGYDMEDELEIFNSLSHIGSDDSLAPKYLKYSVKTKNTGWNKIQASVSSYKHFIEALEESMFYQQRSKSEHKYSSNKISELLNMKRVQRDSLEAFFYVNLANQQRYFTFSEEDLRQLPHDISEYFKKLEKGVEQHYTKTFNQAQVSVMFPVAMGVPFIYKYKEPTLIHIQGRAKGEFTRPSKEQPQYSAQIAKEVQFTYARNIDGDVGFMDTISNQHVSVGVVSKLQFNVPIKLDIQVKPKEFKIKAEPLHPEQDITVIHYSVWPYSTIHKKDSLLPISLDPNTKVIERQKKTLSFDNKFGQATGTIFHYQGYSYSTDYRNIGTIFKSPDFMTNVAAFVSQEDIALTHFNIRYLGKQSQNKAVTFTAVYDEYYNQKEGGEWGPAVEQEDLSPNSEARRQAMAKRVSAGINTANAKVVDFSATFEGSHKADYLLTAAVSQSPVDPKVQYAFFAGKNSAQHGKSQFNVVANAKFPKTNALNFLQVLDNDLKTTFEADIKFNHNGNIHLYAEAERSKKYTEELQKYPLAKQCVEDIARNNQYTDTCHKMIIKAHAPDHFKFTFNYKDISNGYKNYTYNAYMLAKYFGFWYAEVNPAKSLPEGKVEIELTASYLDRTFDASMMSKYGYVRMQNLPLLRSTPYALAIYQPFQPQERVANFYTSYQYQPYCSVDGSKIRTFSNRSYDYILTSSWHVVMYDEPQEHGIGHEVVILARKPKPNYQEVYISYKSETGKDFEVEIQPAPEGSKQPQVNVKTNSKKVSEGEFTIYWNDVEQKPFLEYYTQQDGVLMLNIEEYKLRAMYDGQRLIVLASANRQSARGICGSITGEPRDDYLTPEGLVDNPEYYAASYALYDEYSDPKTQELKTKAKQEAYQPKNKYTTVLRSDPEWQQDKTAASSPEEDWGSETAYRSRSYDKQRGPYVVKHQVQYYENHGEICITTTQLPVCQSHCTVMEYRIQATQVTCRPKLDQQFRSYRDQIKQGQGPVVTGVPKVKQFKVPAICNA

>sp|Q868N5.1|VIT_APIME RecName: Full=Vitellogenin; Flags: Precursor Apis mellifera

MLLLLTLLLFAGTVAADFQHNWQVGNEYTYLVRSRTLTSLGDLSDVHTGILIKALLTVQAKDSNVLAAKVWNGQYARVQQSMPDGWETEISDQMLELRDLPISGKPFQIRMKHGLIRDLIVDRDVPTWEVNILKSIVGQLQVDTQGENAVKVNSVQVPTDDEPYASFKAMEDSVGGKCEVLYDIAPLSDFVIHRSPELVPMPTLKGDGRHMEVIKIKNFDNCDQRINYHFGMTDNSRLEPGTNKNGKFFSRSSTSRIVISESLKHFTIQSSVTTSKMMVSPRLYDRQNGLVLSRMNLTLAKMEKTSKPLPMVDNPESTGNLVYIYNNPFSDVEERRVSKTAMNSNQIVSDNSLSSSEEKLKQDILNLRTDISSSSSSISSSEENDFWQPKPTLEDAPQNSLLPNFVGYKGKHIGKSGKVDVINAAKELIFQIANELEDASNIPVHATLEKFMILCNLMRTMNRKQISELESNMQISPNELKPNDKSQVIKQNTWTVFRDAITQTGTGPAFLTIKEWIERGTTKSMEAANIMSKLPKTVRTPTDSYIRSFFELLQNPKVSNEQFLNTAATLSFCEMIHNAQVNKRSIHNNYPVHTFGRLTSKHDNSLYDEYIPFLERELRKAHQEKDSPRIQTYIMALGMIGEPKILSVFEPYLEGKQQMTVFQRTLMVGSLGKLTETNPKLARSVLYKIYLNTMESHEVRCTAVFLLMKTNPPLSMLQRMAEFTKLDTNRQVNSAVKSTIQSLMKLKSPEWKDLAKKARSVNHLLTHHEYDYELSRGYIDEKILENQNIITHMILNYVGSEDSVIPRILYLTWYSSNGDIKVPSTKVLAMISSVKSFMELSLRSVKDRETIISAAEKIAEELKIVPEELVPLEGNLMINNKYALKFFPFDKHILDKLPTLISNYIEAVKEGKFMNVNMLDTYESVHSFPTETGLPFVYTFNVIKLTKTSGTVQAQINPDFAFIVNSNLRLTFSKNVQGRVGFVTPFEHRHFISGIDSNLHVYAPLKISLDVNTPKGNMQWKIWPMKGEEKSRLFHYSVVPFVSNHDILNLRPLSMEKGTRPMIPDDNTSLALPKNEGPFRLNVETAKTNEEMWELIDTEKLTDRLPYPWTMDNERYVKVDMYMNLEGEQKDPVIFSTSFDSKVMTRPDTDSENWTPKMMAVEPTDKQANSKTRRQEMMREAGRGIESAKSYVVDVRVHVPGESESETVLTLAWSESNVESKGRLLGFWRVEMPRSNADYEVCIGSQIMVSPETLLSYDEKMDQKPKMDFNVDIRYGKNCGKGERIDMNGKLRQSPRLKELVGATSIIKDCVEDMKRGNKILRTCQKAVVLSMLLDEVDISMEVPSDALIALYSQGLFSLSEIDNLDVSLDVSNPKNAGKKKIDVRAKLNEYLDKADVIVNTPIMDAHFKDVKLSDFGFSTEDILDTADEDLLINNVFYEDETSCMLDKTRAQTFDGKDYPLRLGPCWHAVMTTYPRINPDNHNEKLHIPKDKSVSVLSRENEAGQKEVKVLLGSDKIKFVPGTTSQPEVFVNGEKIVVSRNKAYQKVEENEIIFEIYKMGDRFIGLTSDKFDVSLALDGERVMLKASEDYRYSVRGLCGNFDHDSTNDFVGPKNCLFRKPEHFVASYALISNQCEGDSLNVAKSLQDHDCIRQERTQQRNVISDSESGRLDTEMSTWGYHHNVNKHCTIHRTQVKETDDKICFTMRPVVSCASGCTAVETKSKPYKFHCMEKNEAAMKLKKRIEKGANPDLSQKPVSTTEELTVPFVCKA

>ACU00433.1 vitellogenin [Bombus hypocrita]

MWLPLTVLLLAGIVSADYDHGWHVNNEYIYLVRSRTLVSLNELSDQHTGILMKALLTIQVKDPQMLTAKVSQSQYARVLKSLPEGWDTEISDQMLELRDMPLSGKPFNIKLKHGVIRDIIVDRTIPTWEVNILKSIVSQLQVDSLGENAIRTSETQIPTDEYPYGMFRAMEDSVGGKCEVLYDIMPLPEPNLYVQPELVPVPDLKREGHYIDIRKSKNFNKCDQRMNYQFGITGNKYWEAGSNKNGKFFSQSAMSRIIISGTLKSYTIQSAVTTNIMYINPRFYDHEHGMVASRMNLTLAAVKKITNPLPKPNSPESTGNLVYIYSNPYSDMEERRVGKVVEDSDNMMLSDSISSISSSEEATKGQNYRSLSSDSSSSSSFSNSEEDHYWQPKPTMEDAPQNPLSSIYIGYMGKYIGKSNEVDVVAKSKELISQIANEMEDPNDVYKNHILEKYTILCNLLRTMNKEQMLQVDKHVRLSPHELKSMDKTQVLKQNAWTVLKSVVAQAGTGPAFLVIKNWIEMKEVDSKRGADLLAKLPKTVRAPTAEYIMEFFKLATSETVKNDLALNSSAIIAFSELVYNAQVNRKGLHNHYPVHTYGRLTPKHDKTVTEYYIPYLEMELKKAVESGQSTVIQTYIMALGNIGHPKILPVLEPYLEGKVRVTVFQRTLMVSALAKLAENFPKLARSILYKIYLNTMEEHQVRCTAVFILMQTDPPLTMLQRMAEFTKIDNSKHVNSAVKSTLESLANLKDAEYQTLAKKARAAKNLLSPSDYSYHYSHGYITESIMDDGNIISHMMLKYIGSDDSLIPNAIYYAVFSAYGDFKLPPFEVVTMVSSIRSILELNTSPEDKERIRLAAEKIAEQLNIIPDEFIPLEGNIMWNGKYGARFLPFDQTYLSLLRELLLMYLKGESEGKLINRLGSYDITYGFPTETGLPFVYNFELPMLLKITGTMNNDAKNLKTIKMKTDFRILYAMKIQGRMSFVTPFEHQEYIAGIDVDFNLHLPMKLTLDIDLPKQNFEIKIWPLKGEDKARLLHYSVVPYVANHNILSLRPLLTEKTAQMIIPDDIYLDTILDSDLLKVVLEADKSYDYNNWLDLDIDNFFNVVIAPWSSDNDNYCKVDVFMNLKREQVAPFVLKMSYDFMEMAPTAEDAKLWTPKATATEPSDKHPDSEVRRKQWMNEAAKGVKSAKSQVIDIRLEVPVTSEETIMNVITVATSNSEIEKKGRTLVYWSCETLFEVCAASQTKVTPDNTVFYEEVAQLKPKVEFNADFRIGKVCSTGEQLNINSVATQSKELRERIKNSSLIKTCEKQMQQGNKILRACQNAAAISMILDQITISVDFQSQHFINFITKALNVMMNIDYLNEVAYIHTELSDLKVAGKRKLDIVANLTNNLESADVLISTPNMNIYANDIDLSALEISVEDVLMAADENMDIQNLLYNEDEPACILDKTRAQTFDSKEYPLRLGKCWHVVMTTYPRVNPNNPNEKMRMNKFDSVSILTRDMENGQREMKVLLGDKEMKFVPTSSQPKIFVNEQLVKVTKDMSWQERMDDEVLYEIFQINDHSVGLVSDEYELNLVYDGKRIMMKAGDKFRKAIRGLCGNYDGKLINDFMAPEKCVFRKPEQFIASYALNKEECESEFFENSKLPIDQDCVYEEKNHLSNVISDKESGRRDTEESNWGYHKQTKSKQCTIMRTRIKETGNMICFTIRQVPSCAPGCWATEMKSKDYQYHCMKRNAASLALKARIEKGAKPDLSQKSVTLTEPINVPLACKA

>ACM46019.1 vitellogenin [Bombus ignitus]

MWLPLTVLLLAGIVSADYDHGWHVNNGYIYLVRSRTLVNLNELSDQHTGILMKALLTIQVKDPQMLTAKVSQSQYARILKSLPEGWDAEISDQMLELRDMPLSGKPFNIKLKHGVIRDIIVDRTIPTWEVNILKSIVSQLQVDSLGENAIRTSEMQIPTDEHPYGMFRAMEDSVGGKCEVLYDITPLPEQNVYVQPELVPVPDLKREGQYIDIRKSKNFNKCDQRMNYQFGITGNKYWEAGSNKNGKFFSQSAMSRIIISGTLKSYTIQSAVTTNIMYISPTFYDHEHGMVTSRMNLTLAAVKKITNPLPKPNSPESTGNLVYIYSNPYSDMEERGVGKVVEDSDNMMLSDSISSISSSEEATKGQNYRSLSSDSSSSSSFSNSEEDHYWQPKPTIEDAPQNPLSSIYIGYMGKYIGKSNEVDVVEKSKELISQIANEMEDPNDVYENHILEKYTVLCNLLRTMNKEQMLQVDKHVRLSPHELKSMDKTQVLKQNAWTVLKSAVAQAGTGPAFLVIKNWIEMKEVDSKRGADLLAKLPKTARAPTAEYIMEFFKLATGETVKNDLALNSSAIIAFSELVYNAQVSRKGLHNHYPVHTYGRLTPKHNKAVTEYYIPYLETELKKAVESGQSTVIQTYIMALGNIGHPKILPVLEPYLEGKVRMTVFQRTLMVSTLAKLAENFPKLARSILYKIYLNTMEEHQVRCTAVFILMQTDPPLTMLQRMAEFTKIDKNKYVNSAVKSTLESLANLKEREYQTLAKKARVAKNLLSPSDYSYHYSRGYITESIMDEGNIISHMMLKSIGSDDSLIPNAIYYAVFSTYGDFKLPPFEVVTMVSSIRSILELNTSPEEKERIKLAAEKIAEQLNIISDEFIPLEGNIMWNGKYGARFLPFDQSCLSLLRELLLMYLKGESEGKLINRLGSYDITYGFPTETGLPFVYNFELPMLLKITGAMNNEAKNLKTIKMKTDFRILYAMKIQGRMSFVTPFEHQEYIAGVDVDFNLHLPMKLTLDIDLPKQSFEIKIWPLKEGEDKAQLLHYSVVPYVANHNILSLRPLLTEKTAQMIIPDDIYLDTISDSDLLKVVLETDKSYDYKNWLDLDIDDLFNVVIAPWSSDNDNYRKVDVFLNLKREQVAPFVLKMSYDFMEMAPTAEDAKLWTPKATAMEPSDKHPDSEVRRKQWMDEAAKGVKSAKSQVIDIRLEVPVTSEETIMNVITVATSNSEIEKKGRTLVYWNFETLFEVCAASQTKVTPDNTVFYEEVAQLKPKVEFNADFRIGKVCSTGEQLNINSVATQSKELRERIKNSSLIKTCEKQMQQGNKILRACQNAAAISMILDQITISVDFQSQHFINFITKALNVMMNIDYLNEIAYIHTELSDLKVAGKRKIDIVANLTNDFESADVLISTPNMNIYANDIDLSALEISAEDVLMAADEDMDIQNLLYNEDEPACILDKTRAQTFDSKEYPLRLGKCWHVVVTTYPRVNPNNPNEKMRMHKFDSVSILTRDMENGQREMKVLLGDKELKFVPTSSQPKIFVNEQLIKVTKDMSWQEKMDDEVLYEIFQINDHSVGLVSDEYELNLVYDGKRIMMKAGDKFRKAIRGLCGNYDGKLINDFMAPEKCVFRKPEQFIASYALTKEECESEFFENSKLLMDQDCVYEEKNHLSNVISSKESGRRDIEESNWGYHKQTKSKQCTIMKAHMKEIEHMICFTIRQVPSCAPGCWATEMKPKDYQYHCMKRNAASLALKARIEKGAKPDLSQKSVTLTEPINVPLACKA

>AAT48601.1 vitellogenin [Encarsia formosa]

MILSHALLLLLVGAACAQSSLEGWKNGKVYEYKVRSRTMTSFNKQSKHFSGIVMEGSLTVQPKGQDTLRAKISKARYAQVHTRLDNGWDSEIPRNQMNSQGLPLSNQPFEIKIKNGVVRDLIVNKNVPTWEVNILKAIVSQLQVDARGQNAEYSKHNQLPEGKQPYAIFKTREDSVSGRSEVTYDISPLPEEVLQSQPELVPMPELRGDDGEHISIIKTRNFSNTHQRISYHSGISGKNSFKPGSNDNGKYLSRSSVSRIVISGELKKFHIQSSVTINKVMHGADSNGNQLGMVASKMNLTLQDVKDSEEQLEAPSDPQSTGSLVYNYNNPLDKTSRRPNRPSPDESRRNSQDKRSEDRENSDESSSSSDDTSKSLYNNDEDNYMQPKPKLTEAPQTPLLPFYVGNGGHSIQKDGKVDVVKSAKNIARQIGQELQKPDSMVEERTLEKFTILTRLLRTMNAEQMAEVKRDLYEPRKSSNQLNQDSKDQRERRNAWEAMRDATAQAGTGPALVNIKQWIQNKELEDNEAAEVIDALAKSARTPTPEYMDALFELVKQQESPKESALRDSSVLSFANLIRRAIVNKRSAHNRYPVHVFGRMFNRENSNLHEKYIPYISEKLKKAVDEGDSKKIQLYTTAMGRTGDSHMLSVFEPYLEGRKQVSPYQRFIMVFSLSKLAETKPKLARSVLFKIYSNTADHHEIRTAAVFLLMKTNPPASMLQRMAEFTNQDSNKHVNSAVKSVIKSLSELDDDDDQSLSRAAQSARPLLTSESFGPEYSRAVIKEYKNPLTQSSVSVEAKYIGGGDSIIPKGAHVSISSSMMGMEDSSTELGYAVSSVQDLMEFVRQQLSADKKQSRRNQSNQEQKFSPEKIARLLGIQGKEPNQVEGLAFLQGTLANRVQAFDNHTLEKIPSELKRMVDEMKQGKSYEKTKLLNHEITVSFPTETGLPFYSTYKTPTVMSVRGEAKLRADSDSQSSIPKSVTASGETRVVLGMKVQERFGFVVPFENQEYIVGLDKNLQVHLPIRSEVEFDKNKKEIRIRLQPRKDAQEVKVLQFKTQPFTAKHDITNLEPVSSDKNTNILHQEKAKKSSVEFNDQNQKQRVQFTWERRTNDPKQEDESSEKKRQNAMESAKKMARSIASMLSPQSTETEYEKYSVKVSPQNDMSMEIRVSADSLSTEQNQSSESDSGSPNAKAPHLERSLGQRERKEKLMKEASKKINSAEANAIDISVQLGTETEPVVSITAAMARSNTDEKSRVLVYAATNLKNNENHHLSAAVESKTPNTLSMDFEETLKSNTPREFDAEVQYGKRSEDSSENSQNKIKIQGKAKQTENRKNEIRQSRDAQECSKEKSRTGNKMSPACERANQRAASVNIGEMTITLESESRLKSLLLRLVDAAESLARDSDRLEVSKDREDKEEKNKLKVSFETSSDDEEIDVTVKTPEAKYQFNDIKMSSGNKKKNQENDRDSEGKEDSENDSLIKSVCFIDKTQVKSFDNRRFPSKLGKCWHVAMTTYPKNDADSPSQQREIPEDKRVSILTRENDEGKKEMKITLGDNEVELKPAPESDSESKDVVAKVNGEKIKLSQSKSHQHKKDEEVEWELYRLHDRSAKLVSKKFDVYIIFDGSRTKVKVGNRYRDSVRGLCGNNDGESVDDQQTPQGYLIQNPLEFAATYALTNEEQCEGPARQNAEKAKKSPRVSLSARPGNVISDREAGRKNEEDSSERKGNESEKQCMKHRTMVQRNKDQICFSKRPVPSCSSRCSEAETRNKKIEFHCVPKSDASEKVADRVEKGANPDLTQKNTSKTEFQKIPVSCKA

>AIU68826.1 vitellogenin [Osmia cornifrons]

MWLPLTLLVLAGLVSADYEHGWKTGNEYTYLVRSRTLTSLDQLSKQYTGILMKGLLTVQAKDSNRLTAKLSKPSSQYARIHTDLPEGWESHISDQMLDLRELPISEKPFEIKLKHGVIVDLIVDKDVPTWEVNLLKSIISQLQVDTQGENIIKDLVKDPSIQIPDDNEPYGSFKSMEDTVSGKCEVLYDITPLAEHVIHMKPELVPKPELKGDGLHIDILKTKNFDRCDQRMNYHFGITAQTNWEPGSNKNGKFLSKSSTSRIVISGNLKRFTIQSTVTTSKMFISPRLYDNQKGSVVNKMNLTLADVKKISNPLTSPSNPESTGNLVYVFDNPFSDMEERRPSQGIGQVDPKQVRTSDSISSVSSSEEALKNQPNLRGSGSSSASSSSISSSEENKFWQPKPTLEDAPQNPLLPNFIGYKGKFIGKSGEIDVVKAIKELVFQIANELEDPSNMVTQETLEKFSILTSLIRIMNRKQIAEVETKMHISPNELKSNDKTQAVNQNAWAIFRDAITQAGTGPALLTIKNWIERRDITTMEAVDILSRLPSTARAPTAKYVNTIFELTTNPKVMNTATLNTTALMAFTELVYQSQVNGRSIHNLYPVHTFGRLTSKHDQTLVREYIPFLAKELKNAVEDGDSPRIQTYIMALGNIGHPKILSVFEPYLEGKEEMSVFQRSLIVGALDKLARNVPKLARNVLYKIYLNTMESHEVRCMAVYLLPLTNPPRSMLQRMAEFTNYDTNKQVNSAVKSTIESLADLTDPEWTSLASKARSVKSLLNTEDYSFRYSHGFVLSQNKPEKNLITDWVINYIGSDDTFIPRALYLATMASYGDFKTPPTELVAMISSVQSLFDTVYKIGRDKHMPEKMAAERIAEELNIITDKPVELEGNLIWKTKFLSRFMPFDGHDVRGMHNYLVKKVMAMKDGGYMNLNKFASYDITLSFPTETGLPFVYTLKVPVLNKMSGSGHLKMGRDHSVNAKAEVRLVYARKIQGRIGFVTPFEHHHYIAGIDVNTQAYAPVKVSLDINVPTKSTQLKLWPLKGEEKSRLVHYSVVPYTSVHNILSLRPLQTEKGTHKVHIDSPKTVTLPVTTTKGLRINLEADKSNEEFWNLGTNNVVNMFPWNLEDDTYRKADIFIDLKQELKEPLILSVSYANMKVAPGTEDTKQWSSIAKAVEPTSREANSEDRRKQFLKEAAKGIKLAKSHVLDLQLQIPGEAKSRNTLTVAWSNSDAETKDRALIYWGFDMPSEEEKTELCAAIQLMANPPYSLFFVEAILSQPKKEFDIDIRYGETCSKGEKINIKGKATQSDDLKEDIKNSPLAKECKRQMEQGNKLLKVCQDAAALALTVNDLDLSVDTRSENLENIVTRAITIVGTKYSDDHTMAFVKPKNAGKNRIDIEAKFSKDMKSADVIVHTPTVDMRLNDLDLNPLDDSTERLSADELGVNALESDQSEPSCVLDATRAQTFDGKDYPLRLGKCWNVLMTTYPKANPNKPEENLALSEENSVSILGRDTQDGQKEVKVMLGQNEIKLLPTGQLPEVIVNGQKVQISRELSHQERKDDDVLFEIFEVGDRSIGIVSNKFEVDLAFDGKRILIKASNKFRNSLRGLCGNYDGEITNDFTSPSNCMLRQPDQFIASYTLTKDQCEGEDLENAKSLKQLDICLPQISSRQSNVISSLESGKSFPESTNWGYHQNKQTDENKRCTLFRTKVIEKNGKICFTTRPVVSCSPTCSPTETTTKNYSFYCTHKNEASLNMKQRIEKGANPDFSQKAVSMSESLEVPLACTA

>AAC32024.1 vitellogenin [Pimpla nipponica]

MWCPLFLVLLAGAATAEHLQAWKTDTEYQYAVRGRTLSALHDVADQYSGIIMRALLTIQPKSDGTLEAKVTKARYAQIHTKLPGGWNSEIPDHKLEMKQFPMSEKVFEIKMKHGVVRDLIVDKDVPTWEVNVLKSIVSQLQVDTQGENLMASKYNQEPEDEGVTAMFKTMEDTVGGRCEVLYDINPLPEFVLQKRPELVPMPDIRGDGEIIDIVKTKNYSNCEQRSGYHFGIPGTNKWEPSSGASGNFLSRSSVSRVIVTGNLKSFTIQSSVSTNKVILSPNFHENRKGMVSSRVNVTLVKIGAPSTGDWSTPANPESTGNLVYNYNNPFAGVGEDRRASRPQDNRNSEEKVRSLYNLYRRNRINDNDDDSSASDSSKSAKSLESNEEQLYWQPKPTLNEAPAMAMLPFFIGNHGKSIHKTDEIEPITMAKTLASQIGSDFQDPNSITDEQTLEKFTLLVRIIRTMSTQQIAEAERDLYQSNNEIDPHDESQSVRRGTWAAFRDAVAQAGTAPALVTITLWIKQKKIRGVEAASVVGVLAKTARTPTREYIDVFFELATMPETIHEPFLNTTALFTFSELVRYSQMDNPSSHTRYPVHTFGRFSDKINPEVFRMYVPYLAEKLKTSIEKNENAKAHTYIVSLGNIAHPKILAVFEPYLEGKMPASTFQRLLMVISLNKLATLKPKLARGVFYRIYKNTGEAHQLRCAAVTALMSTNPPASMLQRMAEFTNEDHSKHVNAAVKSAIESASELETPQWQELAENARNAKPLLNKESYGFEYSKLYLTDFIEREMNVAYQAQASFISSDDSYVPEALFVKARAIFGGFTFPRTSAGAMVSSAKDLLSAFEDAFKGEKTSHKHSHVEFSPENVAKLLKIKSEPNEDVEGAFMLQSAYNNKFMSLDKNTLKNLPELSSKALKALKDGHHFNTARIESYEMTMSFPMESGFPFVYTMKVPSMLKLAGSVKGDSNEDSLDGHVHLRAVYSVQFQSKLGFVTPFEHQHYMAGINKNYQAYVPLRVNVNYEDSDKRVTLKIQPIEQNEKFKLWHHSVVPFTSRHDILKMSPVLKDKETQKIITEEVSKNEYNFGSDKHGVKFTVLAEADNDNSPLNFEDAKWERNPLTQLFHLFVEQPVYHKVDIFMQPSKSSEQSIILSTAFETLQDDTTKYDEDRSKHIKAEAPKVENKKLNDPQRRRKLLKEAAKGIAAADAFAVDIGLEYPGQAPVQAFATLAFASSEVDEKSRGLVYWRLTDAGNDFEFESCTSLEARSPRTSDFGKDMLDQPEFRSRKFDIDLRYGQTCEKGYKVEIQGSQEQTEKYRDTIKRLPTAIECIRAAKKGMKSLPACHDVSVKMTMLDKTRITIKYDKDSREMYQLFDDYLDSMSDADDDLKSSKKRLNAVKDDDKAYDDTVKIAVDLSPNDNNGRFSYESDFVKLEARDVPINGIGPLVNVHPDLETAERLDMDMSDEPVRQRICVLDSHSATTFDESTYPINLGKCWHVVMTTFPKTNKNSGSAEPIDEDMALSILVRDAGDKKKDIKVTLGDKELQFSHADSKNKVTLDGKKVDLSEKRSYRHKNGKDIDFEVLQRPDGTLGLVSSKYDIDAWYDGQRVQIKASGKYRSDIRGLCGNFDGEPDNDFTSPKDCVLLKPEEFAASYALTTKDCHGSALEHARKASQAVCSQKSPRPGNVVSDRDAGRKYSENSNWGYHSRQNSDDEQGKNDSSDHKRCNTLRTKVIEEEDQICFSLRPLPTCAEGCTANRTKPKVMPMHCMPKNIAAERMADRIKQGANPDFSQKSYTKKNGFDIPVGCHAA

>ABO70318.1 vitellogenin [Pteromalus puparum]

MLWSPAILLLLAGAAFASPQNGWKDGKEYTYKIRSRTLAAFNPQSKQYTGIVMEARLTVQPNGDDLLRAKILMPRYTQIHTRLENGWDSEIPQNQMNMQTFPLSGKPFEIKSKNGVVRDLIVDKNVPTWEVNVLKSIVSQLQVDTQGENAIRSKHNQFPEGKQPYALFKVMEDSVGGMCEVLYDVSALPDRVVQTNPELVPIPELREDGDIISLVKTKNYSNCDQRVSYHFGLNGRNKWEPSGNGNSKYLSRSSVSRVIVSGNLKRYTIQSSVTTNKVVLNPDQQENQQGIVASRMNLTLHEVKDISEQVPPPSNPQSTGNLVYNYNSPTESLSTRRPNKLNFQRKHDHKSSEQKHSAESSSESIDSIADNNDDSYLQKKPKLTEAPQSPMLPFFIGNHGNMIQKNEKIDAVKSAKSVAQEIGNEMEKPDLMPETQTLEKVTILSRLIRTMNAEQIAEVQRDLFQQRQSSNQLHQRNRAENSRRNAWVAFRDAVAPAGTGPALVNIKQWIQNKQIQGTEAAFVVDATAKSARTPTTEYMDAFFEMVSMQETKKQRPLRDASILAFADLIRHAMVNQRSAHNRYPVHAFGRLISKDSKNLLDKYLSYMADELKTAIDMGDSRKIQVYVAAIGRTAHPRMLSIFEPYLEGKKPVSQFQRLEMVMAMYKLATSHPKLARPVLYKIYSNSADHYEIRCAALFTLIKTNPPASMLQRIADFTNYDVNKHVNAAVKSVIESLAQLQDEDFREISNAAKAALPLLTSEKYGPQYSRVVMKTFKNSETNSGFQLVATYMGSEDSIIPKGGLLVLSPVFRGMKVPKIQIGGMVNSIQDTWKFVEQKFKNFQKESQSSRKAQQQKFSPENISKMLGIHGQEPEQIEGHFFLTHRNGDHFISFDNHTLEQIPEQLREMAESMKKGLDFEETRLSGYEVTISFPMETGYPFTFTLKVPTILSVNAQSKLRTDSELSNNEIPNSATVSGKARLVYGLKVQKRLGFVIPFEHQEYIAGLDKNIQLYVPLQSEVAFDKSKNEARFKLQPHEDEKEYKVLQFKSQPFTAKHDILSLQPVSTDQNTHTVNKERASPLSFELNDQSGKQRVQFTWVSQNDNQNDDSRNNKERNAIVAATDLASSVASLYYPISSEKATYEKYSIKLTPSSDMNVEVKASYDSLITENKESESSENWSPNAKAPHLSQSLSQGERKEKLLSEVAKNINSAKAKSVDVSLKLNAGIQASVALTAAVSFSNVDQKSRALVFASAKDQDGQNYHFSAGYEAKNPDVDTLDFEETLKANTRREFDAELHYGKGSGDSSDDFKDIIRIQGEAKQSEERKNQIRQSREAEECNREHNKSGNKMTLACQQANKRASSVDSGEITITFENRSPLKQWGMGLVDNAEMVSQDYAKVQKNREDKHDSNKIKIDFQMSPRDDKMDITLKTPEGKIEILNIDTMVDNSQQNLGSQRSQNDMQTSESNIFQISSTCSLDKTMAKTFDNHRLNLNLGKCWHVAMTSFPKNDPDRPSQQLSIPNNMHVTVLTRDNENGQKELKITLGDKEIELTTSGPHEPRAKVNGESIRVSKQKSYQEKQDNQIAFEILELSDKSIKVVSDKYNVKIVYDGSRAQIKAGDRYRNSVRGLCGNNDLEPENDQQTPRGCMLQKSDEFSATFALTSESQCQGPAVQLSEKAKNSQCTFESTRPGNVISDTEAGRASQSGRDSDSDDDDSRESRRCMTHRTKVVMKNNQICFSLRPLPTCSSSCRSAETKPRAVQFHCVARSSASEKVAERVEKGANPDLTQKSVSYTETYQLPISCRA

>AER70365.1 vitellogenin [Vespula vulgaris]

MRSRFILLLLGVAVSNLDNNIEHGWKVGEEYQYLVRSRTLTGLQTLSDQYAGILMKATLRIQCNSPDTLRAQLLKPQYAQIHKKLPDGWDSRISDQMLEHKHLPLSNEPFVIKLKHGVIRDLIVSKNVPTWEVNIIKSIISQFQADTQGENLKGNKNTQIPEDDNPFATFRVMEDCVSGKCEVLYDVVPLTEDVLQHRPHILPKPELRGNGEHIYITKTRNYDKCEQRMDYYFGISGNANWESDIRNNDKIMKKSSTSNMVISGNLKNFVIQTAVTTTEIIMKPRLVDDQESIVISKMNVTLVSVNKVSSPIPAPNNPESTGNLVYTYNDPFSKTVQRRLGRPSVSPNSMSNELDSSADSDEDLRLMREKYGKSDKMNVVSDEDKAFRQMKPTLHEAPKNPMLPLFIGNNGKAVVMSDKINSGKMVMTLVQEIASEMEDPNVMPDRETLEKFTIVSRLISSMSLEQINKAEGSLHSVWNEIGSDETNRMKKENARAVFRDAIANAGTGPALMTIKRWIEKKEIEGCEAADVLASIPKTARTPTAEYVDAFFSLASNPEVQKQTCLNSSAVLSFAELVHRAQVSNSSIYNHYPVNVFGRLSSRRNDAVLRKYIPFFAEELKKAIKDGDSPRIQVYILALGLTGHPKILNVLEPYLEGKERVSTYQRFLMVMSLRKLCEVKPSLARSVLYKIYLNTWDVHQIRCAAVNLIMRTNPPLDMLTRMAQFTNTDFSGQVNSAVKSAIESAANLNYPEWEELTRNARKVLHLMNTESDKYYYSESHFTEMEENDQLSYRMMLNYIGSVDNVIPLSTHFALQPSYNGFLSPVYELDMSISSVKSLLEMYWHKSEKGDTEESFAEKTAKMLHIESDNVEQVEGNVFFKTPYLNRYFSFDNHTIERILHDMVSPRHSHHVNMNKLLSYDITLSFPTETGLPFVYSLHVPTIKKFSVISKPDMKSKFDVRLLTSTKHQGRVGFITPFDHQAFLSGIDNNMQVFLPCKLDFHLNNEKSRLDAALQPLKHNSKTRLGHFSVIPYTSQYEIMSLRPLLLEKNTHRIQEKKTTHIRIPQNPNSIFSVEVEADNLAEKIQQWLRSENKWEDMLSPSSLALGTYEKIDLFVKPDLQENEAVKFTATLDTKEIRSNNLDTNDESWKSGNKVLKTMHQALDSPARRKEFLQEVVKGINSGKAYVIDAGLEVPGLWKSNHACTLCLASSNDENKFRSIFYWYTNIPSQDITYQMCVNGQTRSSPTTPFDYKKILDSNPTNEFSINIQSGRTCTDNSLVTIKGQIKQSEDYKTYVQESRIVKRCDENVRNSVKDCQKAAEMAKNLNEIDMTITKHNSAEESDTELEKIFHGTKKMLTGLNVRVVSEIQEHSDVNDNDIRVQIKLSPNMTSAEGMASKSGQILTFSDIHIDMGIDNDDEMNEIEKGILHGATCTLRNNEAETFDGKVYPLKLGKCAHVLFTTYPRNAPNEPNKRMSIPENMKVTVIAEETENNKKELQILLGNDEILFKSSGTEVSAWVNGQKVKCSQKESYQHIKNDETLFEIFELPGPAIKLISDKYDIKLAYDTDHVQIEVPHTYQQSVRGLCGDFDGRSENDFVTPKNCMLQKPEEFAATYILKERCEGPALNNAKKAERSKCIRKVLRFSDVISDGEAGRPYVNWKQWGYHKKENKKQCNTYRTQIITKDDNICFTIRPVPTCSSGCKSVVTKLKEYQLYCLPKNDSSLGMKKRIEQGANPDLSQRTPTDNAMISVPLECVAT

>AAA27740.1 vitellogenin [Anthonomus grandis]

MWSTVALCLLVGLSYVSSSSPAWKDNTEYVYSVNGRTLTGLEETADQYSGVFLEAKLHLSIRPDGKLQGRISEPKFAQILSQLPDGWKSEIPDSQISYKQLQLSQKPFQLVLENGLIKRLIVEKDTLNWEANIIKSIVSQFQMDLQGENALQNPTSSFPTNEYMDAVFKTMEETVTGKTETIYDIHRLPEYLVQSQPWIAPQYKLKGEGDLIEVIKSKNYTNARDRPSYHYGFGEIEESEPTANKMGQFFIRQSNSRAILTGKPSRYIIQSTYTVNKIMVNPILKNKEMGSITSMVNVTLLEINNQQQQPEELSNPLDIGNLVYTYGQPKNNQVHSKLNENLMEDSSSEESSEQEMTHRRFRRSANSLTKQWRESSEEWNQQQQQPRPQLTRAPHSPLLPSMVGYHGKSIKENKDFDIRQNVENLVTEISDEIKQSEKTISKHTLDKYTILNTLVRLMDEDDIQFVAEQMYSQMKNGQQRYTWSIFRDSVAEAGTGPALLNIKKWIETKKIQKTEAAQVIGTLAQSTRFPTEEYMRKFFELATETQVRQQETLNQTCILSYTNLVHKVYINRNESHNQFPVHAFGSFYTKKGREFVKTTVIPHLKQELEKAISNADNNKIHVMIRALGNIGHKSILNVFQPYFEGEKQVSQFQRLMMVACMDRLADCYPHIARSVFYKIYQNTAELPEIRVVAVHQLIRANPPVEMLQRMAQYTNTDSQEEVNAAVKSVIESSCKLESSKHAELRKAAQSARPLLTKKQYGMEQSYINLRDYVAEQMGLELHVQRTSHSSAESSFPKIMKFQLHQHNHGMKQHILSTGGMISSIRELLNVLYRQTEVFQQEKSQRSQEQGKDNEWSSANIARLMNYERDEREQLEAIIYAQVEDVQKLWSFDNQTLEHLPEVIRQQEEIYRQGKDFSYVKLKQLNEMALSFPTEMGLPFLYTYDVPVLMKVEGKIRALANPAISRNNKLTKPEQISTEIKARVTCTGKTQSHLSFVTPFDHQIYMAGYDKNMYVSIPVNARLEMDVKSKEAKIEFEVEQQQQDSRLVHITSTPYTSRSDVMAISPVALRPNTYVIKSHRNNHRYFDFNFGKKETGLTFRGWGHHPEQSIGFNDLVSMWQSRGVAGVWEQLWDKCSTEYSEATISFIPSQSTTRKATFRINVDQKYQKQPETQSPEDLLTLNQLSSKLQKDEPKQRQQEIKKHVGSGINSALLSCSDISLEFEGDKKYEHVVGFAVAKSNADPKSRVMFYYKNKNENKQGALEIRSEIPNTNGLNLDDSLDTEPSTKYNMRLQYGNSENDAFEISAQAQLSRSQERKQYLINQDPLYHVCKEQMQQKNFQLPACQNMTIKANFLDHIKYQVQYQKLNWKLVETLEGMFKGLRVLYYPMTEIKSISSVGQNVVEGEVQFQPEDFRQVNVTVRNTDEETVFFNISLNNELLRTLLVPHPVFHAKCRFAGLMQGQQNYRPTCVIDQTTAQTFSNKTYSVNLDKEPTVVMQYVPKDARVNGQQSKSVEQLLRESIENYVVLVRQVAANQKEVIINLNHPRTQGKTVKIEMKPSEDRQKSARNPAAKVTIDGQEMHFDDKQIADKCDGYVQVYALPNGEVKLEVEDAFYLIYDGQRVKVTATGNKLRDSVYGLCGRFSQDKHEDFTVPSNCVTRDTRKFVESYQVEKGQQWRNSPSEQCIKKVLPLYTNVISNQNGSQMRTKLASGTVMKHRYIEENGEICFTIRPLPVCNTSVKQVVTKNVPVHCIQGTKTAYYYKSLIDQGGNPDFSRKSETRTARMEVAAQCN

>AMK38869.1 vitellogenin 1 [Colaphellus bowringi]

MWSQVVFCLLVGLALGSNTPGWKDNTEYVYKVRGRTLASIDEVSSQYSGILLRATLRIQTRPDGKLHCVITKPEYSQIHSQLHDGWKTFIPDSELTWKPLVMSDKPFQVEMKYGVITDVIVNKHVSNWEANVIRGIMSQFQLNTSAAKLNSLSENQQESAVFRIMEDTVTGNTDTLYEIKQLPEYMLQSKPWEARHLQWKTNGDIIEVVKHKNYTDSVELPAYFFGFEGLKNWYPATNQMGEFFIRDSMSRAILTGTLKRFTIHNSYTVNRIMVNPTLTDKQKGSVISLLNVTLIDVKSQEQILEEIYDPVRLGNLVYSYETPYTHSEVREKKPYQTTEEWERDQRPAWQRLRRSIYSSDEESFNDVSEESYKREFSSMSEAPEWPFLPFTSGYKGKSIKFAIDIVNSVEELTREIAKELQDPVKTLEQSSASKFVTLTSLIRIMNEEELKKVSHSLYTTVEEGIKYDTWVIFRDAVAESGTAPAFRLIQSWIKTDKINGQEASHILSTMTKAVRYPTPQFMKAYFELIKSHEVRSEWPLNSTAVLSYADLVHKVYFNKEYKKQFPTKSFTNLRTKEGIQFVRKTFIPYLTQQLHEAISQAETQKIHTYIRALGNVGEPSILLSFEPYLEGKKQCSQYQRLLMLISLDTLVRDYPDEVRPVLFRIYQNPGETQELRVAAVYLLIQAQPTTEMLQFMASYTNVDTQEYVNAAVTSSIRNAANLRGAIHRNLHFAAIAAEPLLTKKVYGLQYGSHFLRSYFVEELKTEIRQALKTWGSDDQYYPKGFQMDIFTNWGGLRSKLAHVQAMISSVEELVRVGEEKTLKYQQRKQKQEENAEEQAQYPWSSQNIASILNMKPEIREQLEGSIYFGELGSIIKMFSFDNQTFDKLPEIIRMYEQEFEKERKVNYLKLVNRRDLAVSFPIEMGLPFLYTYDTPAIVRVQGKVRAAATPEISQGGKLYAPERVNFKSDMFFTVASKEQGRLSFTTPFDHQQYVAGYDKHWQIHVPLKSKVEIDVKNVQMKVEIEPKEAHHDAQLFHYSSWPYTSKADLLNFGFSQINTHVILPREMRRFDVTLGQRQTGLALRVRMDYERRSFDSSILENLFSKQGLQTDDFFALWDNAAIHYSHLNVTYLPNKSTTRKIVMKFGYQGKYAEEGYDSSSSEEITSEKIMQKVGTGIKNARIEALDASVEFKGERDIKYTLAGAFGKSNVHPKSRMAVLFKMNSNDDEFKPWEAHFESESRIPNTGAFDLNEALETEPEVDTKVEFSFGHSGKPFTKIQADIEHRRSEERKQHLKGLPMYLQCKHEMRDGNKQLPACVNATMESNLLDHIKVKVQYENLSPAFINAYQNAMRYVHLKYWLTFEESRPELSLESNEMVMEARFHPDLKFVNVSVESPLGRSFVRDIAVSDVARRAMVVHPVFHLKNRLFSKALELHTFRPSCVVDKTKASTFNNLTYPIDISKHWTVMFQYVPKLAQRHYQQEYVEEQLNTQTNNYVVLVRKNSESKQWKDVKITLSTPQTESKLIDITMMPRPQVTSGIKALVKVAGQEIEVSQTESYDFHNGYIQIYALPTGEVKVEVYNTFYIIYDGMRVRLTMLNGQLKNDLRGLCGQFNELPTEDFFTPQNCFARDPTKFVRSYEVEGHQGKEARKELAENRQECFRKITPIYADVISEIPARPEESEECEFTQTKYVQEGEKICFTTTPVLTCSRQCQAHGYLTKNVPVHCLAKSNIALLWKSQIDKGLSPNFSQKGVHKKIQMQVPQGCYE

>AKR04341.1 vitellogenin [Octodonta nipae]

MWLQIALCLSVGLAYATPNSAWRENSEYHYEVHGRTLTSLHEISDQYSGILYKADLKVQPKPDGHLQAIISNSQYSQVQLPLGDGFTTHIPHSELSWKPMPMSSEPFQFQMENGAIKGLTVPKGIANWEANMIKGIVSQFQLKINERSESPEEHATHFQVVEDTVTGETETIYKIFRLPEHMIQQEPQAERLMQEKRNEDVYEVTKQKNYTSSVELPSYFYGFAELEQGYPATNKMGSFLTRGSFSRAILTGNLNRHTIQNSLTISEVLVSPTVSDKNKGSVYSMVNVTLIKHQQQQQEQIPEVSNPVHLDSLVYSYGKPFDRSNEVQERSPQSFNKHRSAKIQISENMIGRLRRSLEEADFRPGSEENYKQEYLELQEAPESPLLSFTEGFKGQSIKHKINVVQKAKDLANEIATESHESIDDHKNEESLNKFTILTALVRVMDASEIKNVADELYQNDRQGEGRQSWQVIRDSVAEAGTGPALLVMQEWIKSGKIEGVQASFVLATMSRAVRQPSVQYIKTYFELIKDPKVQSEWPVNDTAILSFSQLLRRVYVDREHSAAQYPVKSFRNFRNAEGLQLLKNTIIPYMKEQLQRAVSQAQTHKIHVYIRALGNIGSPEILAAFEPYLEGEKQASQFQRLLMVLAMDRLAEKYPQEALSALLRLYHNTAETQEIRVAAVYQIIRTKPQPQLLQYMATXTNIDPHEHVNAAVKSSIETLCSLRGQEYQELRQAAVAAKPLLTQKTFGLQFGGKYIHQHIIDEMKVKYKEMWQFIGSDDQFYPKSMKFSLHWNAAGLKKKIISAQAMISSIDELVHVFQQKTSSFSQKHEEQKQKHQQQLQHQFSSQKIVELLGLKPEEREQLEGNLYIEGGAAESMFSFDNQTINRIIKEITKFETELERGKQIRFLKMSNEEEMAIAFPTETGLPFLYTYDTPFMMKVHGHIKAGANPKIGQEKLQMPDSVQMEADLRIVVTSKIQGRISFITPFDHQQYVSGYNKYLHYHVPVKGKLELDVKKMQVKAEFEKGGSQEPTVLSHYCTKPFVSRVDIVKCEPVDKKSNPHTHFIEQRNRQTVEKKFGEEQTGLAMHVRIDHEKPLFNFAKMKDMYRREGATAAFLGLLKDSDIQNSEVTIRSLPQESSTRKCVISVAYQSKYQPKEARGEYSDSRNDQPGQSEFDKWQSGTPQERQRDAFNRVSNGIRDSRMQSADVSIAFQGDRNIKYTINGAYAMSNTDAKSRMRLTYRRRSDKSSDEPYIVVFAADNVAEHTNGLNMEYSMENEAKIESKMDIAFGPESGRPAKISAQMEFTRSEERKDYLKEQPMYKQCRSEMNSGNNQLPACVNITRAANLLDRVQARVQYENLPQELEGALKTLYYAAQYEYFPSTLSERQQRSQRDNEVSLQAKFDPDLRSVNVSVATANKGLQIENIPVNEWVKEVLVLNPAFSTRARLMAKTWGMNSYGPYCTVDQTQISTFNNKTYPADVEDKWTVMLQYLPKEARFEEHRHSLVEQLKHQQENYIVLVKRNGVSSKDVKITLSSPQTKFQIVDITMTPQQHQSQQSGRAKVTVDGQQIQIDEKHSYSDENGYIQIYPLPNGEIKAQIMDEFYVIYDGQRVMLTVLNGKFKDLTRGICGQFNNAKSEDFLAPDLCYTADHHKFIKSYEIEGSQGKQVRQELKSGKDRQCIRKDFPLYVDVVTGGNTQSWQAQVSGQCTEFRTKYVQQNGEVCFTIRPMPVCVEGCEPTESVERDVPVHCVQKSSVAQLWKGQIDNGGNPDFTHKSESKTLAIQIPKRCV

>ALN38803.1 vitellogenin [Rhynchophorus ferrugineus]

MWSPLALLFLVGYALASTPAFKDNTEYVYDVNGRTLSSLHEVSDQYSGIFLKAKLHLSKRSDDKVQGRISDPQYAPIHSHLCDGWDTEVPESQLSYKQLALSGKPFEIGMDSAGLIKNIVVEKEVSNMEANIIKSICSQYQLDVRGKNAIDSPINDLPSEDKLDGVFKTMEETVTGETETTYKMHPLPLYILQSQPWLVRKDDLQQDNDRVVEVIKSKNYTNSEEKPSYHYGFGDIHEHEPTANSLGQFLTRQSHSRAILTGKPSRFTIQNSYTVNKIMIRPTFNNKERGSVISMVNVTLREVKNQDQKPQDLSNPNDIGNLIYTYDNPFSQNKDAKQKRMEKYHSSEERSDSEEETSWGRRSRRHISQHIHRHQEGNHDPKDSQQQQQKPRLEQAPSSPLLPYYMGYHGKSVKQSSDFDVKQNVQNMAQDIAENLMDPDKILKQDTLSKYVMLSSLMRLMDKDEIKQVSEQLYSPSGKGKDRLTWEVYRDALAVSGTGPAFLHIKELIESKKITKGEAADVVATMAKSVRTPTEDYMKQFFELTKNSRIMEEEKLNQTAVLAYCNLLYRVYMNRNESHSQYPVHSFGSFNSREGRKYVRETVIPYYKQQLDRAISEGQSNKIHLFITALGDIGDRDILSAFKPYLEGTKQCSQFQRMLMVSCLRRLARSQRNAALPVLYKIYQNAGELPDVRISAAYVLFRDPQVLSERLQSMAENTHIEYQEQLNAAVKSAIESASRLESRSRHNLRDAAQTAVPLLNDKLYGADKSHINFRDYVIPEMDSEFHHDFVEIGSPDSYWPKAMKAYARGHVNEIPQQYYDFKAMTSSIKELFDVLYEKTSGSKQAKKLRSQERDGDSKWSSANIAQQMNYFKEEREQLENYIYAEIAGLQSMWSYDNRSIDNLPQAIREYEDTYSKGKEFSYTKLRQIKDLALSVPTEMGLPFLYTSDKPVLVRWAGKIEARATPQISDGQKLSRPDKIKVKISSAFTFSVKDQSHLSFVTPCDHQIYIAGFDRNVQAHLPLSADVDIDVKEGEATVECEVQNPDKDARLLHYSTWPYTSKGDLMSTSPVSLRPNTQIIPPKESQSRYFDTQFGKSQTGMTFRAWGHHPVQSVNLGDLLHMYKAGDMKTLLKWVWDRSSLTQTEMSVAYVPNQSSTKKVTLRFSHKKQYKEQPEKKEQEDFLSYSQLNQKCQGEPKKRQENLLKYVEAGINNGQSHAYEVWMEFDGDKKRQHGCGVTFCKSNVDSKSRTMIYYTGNGDSEKQCSLDVKANIPNTNGLDLTDSLKNAPEAKYEMRMQCGPNENDAAQVSGKVNFKRSQQRKDRVTQKPLYNVCKRQMKEGNFQLPACQNMTIEANYMDVIQCKWQYNNIDRKYSDIVKSVYEGFKVYYYPETKIESIDDQKNNIQVEVRYEPEELRRVNVSIASGDERTTIYNVSLGSDYAKALLVPHPVFHVKSRLVGVLQGWQLHRPTCVIDQSAIQTFSNNTYPLSLGNDWTVAVQYIPQEARRRDQPKQPSVFEQLKDQQENYAILVRQASEDTKEVMITFNHEESEGKTVEINLKCEQSRQRKRSGSDPAATVYIDGKQIQFTDKQSYDLYNGFVQIYALRNGEVKVEIQGAFYTIYDGKRIKVTSTGGKLRDSNRGLCGKFSNDKYEDFTVPADCVVSDPRKFTDSYQVEKSKRPQRDSQECVAKVMPLYARVGFRKSGEARMNLRTRYAEQNGEICFSLQPLPTCKGSPRRTESEPVEAHCIQKTKSALYFKAQIDQGANPDFSQKSLTRHVDMKVHKQCN

>AAU20328.2 vitellogenin precursor [Tenebrio molitor]

MWSPILLCLIVAFAYASEHPAWEENKLYTYEVRGRSFASLNQISNVNSGILIKARLAVQLNEDGKLGGVITDAQYVQINRELSNGWETDIPDSEVNYEPLDGLSYTPFEIELNKGAIRDIFVEKKMRNWEVNMVKGIVSQLQLDTKASNLMVSSINILTQDDSNTAVFKTTEETVTGVAETLYEIHPYPNYVLQTTPWVVPQKELIHNDQVIEVVKHKNYTYSPRQVLPSYHADFEGIHGYQPQNKIADFMSRTSVSQAVVTGSLKAFVIQSSVTTEEIVLRPTLADQQKGSVNTKLTLTLSSVLAPLDQDKLSLSDPVGVGVVYGYNNPFGTDNGPRPADPYDHSVPDVSSESQSADSENSYEKSSRTKRSTKYRKDVTKKLNFASYQADNLDSEKWHQKKPKINKAPASPLLPFTVAHGGQAIKKQQNIVETVRKLADQVGGEFQDPNEILRQHTVGKFVTLVSLVRTMDHEEVNKVATQLYPASLGQSSPAWVAYRDAVAQSGTSPALLNIKEWIKSGKVSGREAAEVIASTANAPRAPTEQHIKEFFEIIKDEDILSKPYVNESAILSYTDLVYKVHSNAEASQGKFPCTVSDPSILKVGQDFVRKTVIPHLTRKLHEAISKADTRKIHVYIRALGNVGQQYILEAFEPYLEGTKKASHFQRVLMVTALDRLVESNPTVGRSVFYKIYRNPSEAEPVRVAAVFQLMRTNPPADMLQRMASYTNVDSSNYVNAAVKSSIESASQLEEARLAPLREAAESAKPLLTTKEFGVQFSQGYLRSYVFKELQPGYEQYLQSFGGEDYAVPKGIKYSLSKVFGGVSQPVLNMQAMTSSVEDLINVYQQQTKEYQDKLREKQNAEVGSRNPWISENTAKRLDIKNEQREQLEGNLYLQANAWQEIFSFDNHTLANLSQVVQELEEEFSKGKSYKYNKMLIGQESSLALPTALGLPFVYVYDRPALVRIQGDVKAHAEPRISTEGHIQKPDRVHAEFEIKATVTGRVQTQLAIITPFNHQRSSAGYIKNYQINVPLSGKLELNVQKNTVKVEVQNPAVNQEKIRLAHYSSWPYTSQGDVFSPRPELNIIRSKNPYMINQIFGQKSTGVALYLHITSDEIIDTAFIYDKLRSQDPLSAILSPWVDDNIQYVHVDVGIDGSLSTAQQVNAEFIYRKEYQPEKPSTNNQEDDQEPDPKPDEAEELDNIPPYPDDPERRINAFLDKLRGQIRSSSLYAADATAEFLGPSKMKYSATVGISKSNVSPRGRFMALFRQLEKQNPKTLAYLHGWSYIINTNGLNMDYAMKMNPVASFRVKGLLDREERLSKASSDIDFFKSKERVHYLKQLEDYKKCKREMQEGNNQLPVCAKMTALANLLDKFTVRTKYSDMDPKVINATYTAYSVLRHSLYPRIHENIVNRDFDGHLDVDGQFSPDLHALNVSVRSEYGRAEIQNVPMGPWYRQLLVQQPVFHAKTRLAAQAYKYDTYRPICVIDKLSASTFDNKTYPARLRPEWTVALQYIPRRPSNEKGKPYQTVLEQLTQQIESYVVYVRAPQNSNSQKEVLIALQMPSTQGKPATISMKPRRSANQPPRVYVNDQEVIPKLDISADFYNGNIQIYRLPNNELKVEVHDAFYVIFDGQTVKLTAVNSKFRDASRGLCGTFDGEQETDFLAPNNCILHKPVNFVQSYTLFWLNQRYTTRRAAAQQQEEEQKCYQKKVTFANYISNRDAGRTDRTHERQSRDSCSRMQTQYMEQGNEVCFTLRPLPLCKDGCAPRATVSKQVDVYCASRSMRSVGVWMNEILKGASPDFSEQTASRKVRMEVPQSCMPRH

>XP_971398.1 PREDICTED: vitellogenin [Tribolium castaneum]

MWSQILLCLLVGLAFASENPGWNNNYHYVYEVRGRSLAGLNQVSNEYSGIIFKAQLYIQPRSDGNLGAKISNAQYAKIHEELSEGWETEIPDSQVSYQQLPLSSKPFQIGLYKGVIRNVMVDKQIKNWEANMIKSIVSQLQLDTKATNLIPSSINILTQEDSNTAVFKTMEETVTGVCETLYEIHPYPEYVLQSKPWVVPHKHLIEDGDVIEIVKNKNFTHSEQSPSYHAGLDGIYGYQPGNKVGKFLSRTSVSQAVITGTLDKYTVQSSVTVEQIVIRPTLADQKKGSVNSKLNVTLLQVAQPVSENEYDVTSPVDVGVVYAYDSPYSTDNSPRPAKQYDHGVAPGSSESQYDTNQYYGRTKRAITRPNLQYTSFSLDNYQPDSIEEEDWHQDKPHFNEAPASPLLPFTVGFDGQAFKKQKNIIETVRKLAEEIGQEFPHEKEILRQHTVGKFVTLASLVRTMTQSEIQQVASQLYSGKAQGLKSPSWVAFRDAVAQAGTGPALYNIKEWILSGKIDGREAAQVIAVAANAARQPTEEYIKFFNDMIQDEKIMSQQHLNESALLSYTNLVRQVYANRGDSHAKYPVYSFGSFRTTRGQEYVKKNVIPHLTRKLNEAINSADTQKIHIYIRALGNVGHQQILEAFEPYLEGQKKASHFQRVLMVVALDRLVEANPRVARSVLFKIYQNPSEYEQVRVAAVYQLMRTKPTSAMLQRMASYTNVDTSDYVNAAVKSSIEFAADLEAPEHYRFRQAAHSAKPLLTSKQYGVQYSQGYLRNYITKESHSLFEQNLQMLGGEDHAVPRGMKYLLEKQFGGVYQQVINAHAMVSSIEDLVSVFQQQTEEYKRQQQEKQHEQSVNYPWSSENIAKLLHLQNEQREQLEGSLYLQMGALQTIFSFDNHTMENLPELVRKWEDEFRQNKHYAYSKMMIAQESSLALPTVLGLPFIYTYDRPALIRVEGNVKVETNPHISSGDSLKKPDNVNVDFDIHAVISGRVQTQFCIFTPFDHQRYSAGYNKNFQLVVPLNGKMELDVQKKQLKMELQNPGSQESVRLAHYSSWPYTSQGDIYTPKPDLKIIHSQRPHRINTVVGDKSTGVAVYLQITSDHRIDPAFMYERLHHHDLVSAVLDPWIDDTIQYVHIDVGIDSGKSTAQQVNLHLGYKYKYQSTQAQEKNANDQITDIPAFADSPEKRQQEFVDKLGKYINNPHVFVADATADFQGQYKVKYSATFGVAKSNVDPQSRFMGYARKVEKQNPQVLAYVQGVSHVRNTNGLNLDYAMEFDPTSTAYIQGLLRKEQRLSRVDAHIELSKSEQRKEYLKQQEEYKECKHQMQEGNTQLPICAKMGIRANLLDTFSIKMKYSDMDPRVINATYKTYSVLRHFLYPRVEENIVEPSSDNHLDIQGQFSPDLHAVNFSINSEYGNVQVKNVQVNKWYRDLFVSQPVFHARARLQGQALKYDTYRPICVVDKTQTSTWDNKTYPSSFSNGWTVLLHYVPRRPSSSQNKPYESVQEQLNELVESYIVYARASEQSHSQKEIQIVLQMPCTNGKVVKIAMKPSSKGPKVLIDEQEVKYDTEHASDAYDGAIQIYGLPNQEVKLEIRDAFYAIFNGQTLKLTATNSKFRDASRGLCGTFTGEQETDFLGPDNCIIHAPEKFIESYTIGQQLMQRGSKMRRSDNEKQCYFKKVYYANYISNQDAGRVIDAERRGRGSCSKLQTRYVEENGEICFTIRPLPVCKSRCQQRGTIYKNVQVYCSRSTSSTNLWKNEIQKGASPDFSLQQVSKTIQMEMPKECLP

>AhVg1 [gene=AhVg1] [protein=Vitellogenin] Agasicles hygrophila Vitellogenin 1 (AhVg1), complete sequence.

MWSLLVFVLAVGVGLGSNAPAWKTNLEYIYQIQGRALASLDTTDKFSGILTEASLKLQARPDGRLQGLILTPQYAQIFSQLSGGWMAHIPEQQVNWKPLRLSRQPFQIELNNGLITDLIFNKGTPNWEVNMVKGMISQFQLNIYQGDLQKGNQNSAKFQVYEETVTGHTDTMYEVSPLPEFKTFDESIPEFQRVDGDQDLILEVLKHKNFSEHSELPSYVFGFGSLPGARAATNTMGKFFIRNSMSRTLVTGSLQKFTIQHSLALNEILASPTLGDKQKAAVYTMWKTTLLKVKPQGQIFDEIPAPIRVGLVYSYDKPYAKSNEIQAKIKNLCKQMQTPLGSAVDTPFLPFSVGYEGRAAKLKLNGVNSAIKLIEQIAKDTQTPSKIPEKNTHGKFIMLISLLRTFDEAELKQVSKELLLSDITSWNIFRDAAAETGYGPALLLIQSWIETHTINGTEAAHVVASMAKSAQLPTTAYIKTFWKLVTNSAVTDQWPLNETAIFAFTELTRKIYFEKQLSFFETHYPINAFEQIRDKEGLKLIRETVIPHFSQQLDKAVGTANTNNIHVFIRALGDIGEPEILKSFEDYLEGKKHCSQYQRTLMVVSMAKLVKSYPKLMTDVLIRIIENTGETSATRVAAVFQFMRTGPSIEMLQHLASSTQTERDEYVNAAIHTSIESISTLNLPEFMELRKSAQLAKPLLKNKQYGIQLGGNFLRSYILENLGIEYEDILQFSNTEQLIPNDLRYSLTANIGGLKTHIWNIQYLVSSIDNLINVFKEQTEEFQNEKRQQREQAEQQGRYPYSSLNVAKILDMKKDVQEQLEGNVFLMDLVSPIRILSIDNRTIEQLPEILKSLERKLRNKQTINYLKFANTDHMVLAVPNEMGLPVIFSYNAPVFVKAEGTLKATSKPELSQNGKIQTPHTLQAHINISLTVGAKIEGHLSFVTPFEARQYISGFDKNLQFQLPVDTKVNIDLEKNEIEIEAKTETAQNENTLLHYSTWPFIGKKEILDQRHNTIIKGEGVRTYQRTLGKEYLGLAFNIDMKHKRNTIKMSDYRFFCEYGIINGLKHLWDDSNIQYSYLNVSYLPRESSTQKIVLKLRYKQRYTQNIPKQPINWLKIAKSTKSLERLEIIANTTASGLKNAASTAIDASLKLKGKPNIEYVISGASSSSELDAKSRTYAYYKMEMEDKQPFQMTFEKNDDVPKVAVMDLQEALKTTPNAKTNMELTYGLGSEMSKIKATVQFSRSEQRKSYIKDKYLYQECIRDERKGNKQSPACFNLTLEANLLNRISADIHYQNIDDSIQNVLEIAYDHLRYKYYPNLEMSTEGLTINDRINVKAEFHQDLRFVNVTVNTEKQSTIFTDIEVNEIVKKAMVVHPTINLQKRVWSYILGIQEYAHECVVDHTQVTTLSNVTYKVNLTENWTVLAEYVPLVSDETQQVISVAQQLRSEPDNFILFIRKTRTSIDERELKIMISGEQTKFSTIDIVMSPSIQSWSPGVAITVNGRPANEYDNGNGFVQIHRLPHGEAQVDVRNRFVLIFDGKRAKFIPTTEGVRKKLIGICGAYNDQPLDDFLTPQNCISRSTDKFIKSYEVNSPDGKQVRQDFQKNSSECLYKSIPSYVNVIKRSDINIFGEDEDPQGNQCLWYQTKYEQINNEICFTIRAVPTCKKHCQTMGYFLKSVPAHCASLSNAAKLWKTQIDDGLNPDFSHKPETRKIDLKFPQLCSK*

>AhVg2 [gene=AhVg2] [protein=Vitellogenin] Agasicles hygrophila Vitellogenin 2 (AhVg2), complete sequence.

MWSQVVLCLLVGIAYASQNPTWTENKEFVYKIEGRTLTGFKEISNQFAGFFFRGNLKVQPRSGGKLQSIILNAEYSPINKVLDNGWKTDIPEEELTWKPLGLSQKPFTMNIVNGKIHSMTVSKKIPIWELNMVKGVASVFQLNTNGENVLPSTINVLPNDNSNNAIFMAMEDTVSGQTETLYEIRPVPEHLIQGKLDQEGLEFPQMSEHDEIIEVFKHMNFSNQEILPVYAHGFGNPRAGTKPGRTHMGDYFTRETGGRALLVGSLRHYTVLQSSIVSRVMMKPTVTDEKFGQVISVIVAKLEDVKSEFEKIESVTEPVELDNLVYEYDPNPFSSDNSVSGLTKRLSQDEESQRFSGAYDRMRTKRSASHDFDTLEQPEVSEAARTPFLGMTTGYVGKSIKNAVNVVKEAQTIIKKLASDILDAEKDHHEKSLERFSDLVTLIRLMNREELDQLSHLLYTQAEEGPEAVMWTIFLNAAAESGTGTALLLFDTLIESDKIDADEAAEAIATLADSAVHPTMAFMKSFFDFVKKDKVMAKAPLNETVLIAFGSLARTAVIDKSYSRSEFPVKSFGSYRTEEHKHFIREEVIPYITEMLHQAVERAEARTVQVIIKVLGSIADLRILTAFEPYLEGKKQASQFQRFLMIVALDELAMIHPEETGAVLYRVYQNPGEHPAVRIAAVYKIMSCEPTAELFQLMAQNTNIETNEHVNSAVKHTIVQLSQLDANLFPELKKAAQAAVPLLTESSFGLNYGANYLRDFVKDELNKNQLEIASFIPSEDSYTPKGYRYSSLSSWNGFFHRDMDVQAYVSSIDQLLDVAYQQTKKTQQEMKEQERFSAEQLRNPWSSISIAKKMNMKAEEREQLEGYLSWGFGPLTKILNFDNETIEQAPKMIRKIEEEARKGKKMNFLKFTQTEEMTLAVPTAMGIPLVYTYKNPMLVKFRGQIQASADPQISDGSSLHAPNQVKLTAQISATVSGKIQGRLAFVTPFEHKVYISGFDKHLQANLPKTQAEIELDLEKQTVEIMVEPKQETPKQLLHYRTWPYTGIKDLLNFEPLTSQQDVKEIEPRNMQGFKTTVGEKSGFALDIELNHERAFVSNLKLVNSFSKNGLYDGLRALWVDSEIQSSQLNIRYNPQKSFARKITLKSRWDEIYIERAVPLPGPWKLNDEASQKERQEQLMQSVSAGIQSAKVVSLDTIMVVEGNKQIKYILTAAAAKSNIDPQSRAKFSFKRTSRGEIIKDYEAHILLRNEAPNTNGLDVEFTLRNPPKMETEIEAHFGYSKEKLSIVKVEIKHNRSEERVEFLRGSDRFETCKQEMEEGNKALNACTQLIMEANLLDRVEIKVKHENLKEKVKNLIERVYDAVRVHLLPVIDIKARLEKSIIPENEIMMKVKFHEDLRFVNVTVQTKNEKTKAVNIPVNEIAREIVVAHPVFHVRSRVLSSVFGVNAYRDFCTIDNNRASTFSNKTYPAQISNEWTLMALYAPKQARDIDAQQPEEYVAKLLKTQTENLAILVRKSQQSPKMKEFKIVVSSPETNFKVVEINLIPSAGQIKVSVNGQLNDRLSFDLYQGFIQIYPLPNKEAKVEIRGFVYVIYDGERARVTAVSDKLKDATRGICGQFNDQDGEDFLVSENCIARKPKKFVKSFEVQSQEGEQVRQQFSGNDKQCVEKQVPLYVNVITSRESRRKQHSHTPSVSKSNCRMEQTRYVHKNGQICFTTVPLTTCKSGCRPEGSRLKTVGVHCVARSNVSDMWKKQIDNDISPDFSTKRATDTAEVHIPEECHA*

>AhVg3 [gene=AhVg3] [protein=Vitellogenin] Agasicles hygrophila Vitellogenin 3 (AhVg3), complete sequence.

MWSQIVLCLFVGLALASNDNPGWKTGKEYVYKIKGRTLASIEDISNQRSGFVYTATLRIQPITNGKLRAQIVDPQYDEIMGELPEWTASIPKEQHNWQPLKMSGRPFEVEMNNGIIVDLLLTKETPVWEANIIKSMVSQFQLDTTGSNDLKKKWHTLPHNEDDDNDAVFFTKEDTVTGNVETLYQIRPLPDHIVQGNPDEANYLDETKDGQVIEVLKHRNYSNTEELPAYSFGFEGLDWGKPASNKMGNILSRESTSRIVLTGSLKEFVIRKAVTINEIYVSPSLSDKKSAFVGSSVNATLVEIKAPGQPIPQVIRPVRMGNLVYSYEKPFSQSNVVRPKQQRQEEASSEEEADTSSNSLRDQLEQESRIHPKLAVRKPRSVPSSNDKFDYKQSSPKIREAPESPMLPLTMGYEGLSVKHGMNVVQEAQKWARKLGRAMVDTAGHQEEIVDMFASLVSLVRVMNEQEIEQYVEALYSNKPAGIERATWVVARDTVVYSGTSPALLVTKKWIETKKIQGMEASDALTALINSARHPTFAFVKTLFELVKLPKVQSQDILNDTALIQFTDLIRRVYINKEESKRRYPTKSFESFRTEEGELFIIHEVLPYLKEQLAKAMERDETHRIHVLVRSIGNTGTYRFLPIFEPFLEGKKPASQFQRLLMVLAMDKLVDVHPRTAQGILFRIYQNIGERREIRIAAVLQLMRSVPTPEMLQALASYTHLDSDVYVIATVKTAITTAANLKGEQFNRLREGAAAALPMLASHEFGLHDGGNYLRDYVVEELNKVYKSNMQVFPSEDSQLPKGIRYSLRRHMAGLKRRLVEVTAIVSSVENLVQVLSEQTTEYQRQEKEQKRHASEQARNKYSSQNIAKLLDLQPESDEVLEAFLLAQMGSQKGVISFDNRTIEGLTEVIRQMEDEYQRGKEINYYKLSTEEIAVAFPTETGIPFLFTYDRPSFIRIQGKVKASTQPKIAQKNGIQLPEEIKLEADLEIVSSIKIQGRSSFVAPFNHQQYFAGFDQHWQLNLPVSIEATVDTEKMDAEIGFRMQSSKKFNVVHWATRPYISKRAVTNLAPLSTLPETKIIVQDDVQEFKATFGKKSTGIALKVTMQHKREFVDSIALGTIFVEQNPLDIFQTLWDSNTVQHSFINVEYIPEESSIKKTVIRLSYEQEYFPQPKSNSPREWTLNENVPATQRQAEIMREASASIKNVDVASIDVAVEFKGQQNIKYIATGAVAKSNVDPKSRFMVSYKRISNGSPKPQEFHFDVKRSFPTRDTLNMEHALEMQPTGESKVNIRFGNTQEPLSKIAAHLQFNRTEEYVEFLKDLPMYKQCKEEMKQGNKQLPACLNMTMSANLLDAVNVTIKHENLKPTMIGIIERYFHALQAFSGPSAEIKNKDYELKENEITMKAHFHSDLYGLNVTVKTQKQKTIFSNIPVGEWTKQILVAHPDFSVPRRLISKMYNGDEGYVPTCVVDKTHINTFSNRTYPAEISKQWTLMLQYVAGNSHKNEQSLEEQLRRQVENYVVLVRQNQAIPTKKEFKILVSSNSTDFKIVEIDILPARERTLAKAEVRVNGQKIPVNERESYDVEDGYIQIFLLPNHEVKIEINNAFTVVADGSRVRISSDEGKFKNNILGLCGKFNGIEIEDFMTPQTCIAREPKDFVESMILEGSGKQARDTMRGQTKKCMDKKIPAYIDVITSRDYRVKQQYDKTKRCTYFQTRFVEDNKKTCFTIHPQPSCSSHCHPTRDLTKDIPVHCVHRHSPEAELWKKQIENISSPDLSGKEIHKTVQMSIPESCTN*

>sp|Q16927.2|VIT1_AEDAE RecName: Full=Vitellogenin-A1; Short=VG; AltName: Full=PVG1; Contains: RecName: Full=Vitellin light chain; Short=VL; Contains: RecName: Full=Vitellin heavy chain; Short=VH; Flags: Precursor Aedes aegypti

MATDGITSRFGFNERRRTHNRNSCRILEDKMLAKLLLLALAGLTAAYQYENSFKGYNPGYKGYDAGYKGYGYDAGYKYNNQGYSYKNGFEYGYQNAYQAAFYKHRPNVTEFEFSSWMPNYEYVYNVTSKTMTALAELDDQWTGVFTRAYLVIRPKSRDYVVAYVKQPEYAVFNERLPYGYATKFYHDMFKFQPMPMSSKPFGIRYHKGAIKGLYVEKTIPNNEVNILKAWISQLQVDTRGANLMHSSKPIHPSKNEWNGHYKVMEPLVTGECETHYDVNLIPAYMIQAHKQWVPQGQLRGEDGQFIQVTKTQNFDRCDQRMGYHFGFTGYSDFRPNTNQMGNVASKSLVSYMYLTGNWYNFTIQSSSMINKVAIAPSLVNKEPALVYAQVNMTLNDVHPYDKVPMGPAEDLKVFVDLVYSYNMPSDKKNYVRPGNETSSSSSSSSSSSSSSSESSSSSSESVENPKISPVEQYKPLLDKVEKRGNRYRRDLNAIKEKKYYEAYKMDQYRLHRLNDTSSDSSSSDSSSSSSSESKEHRNGTSSYSSSSSSSSSSSSSESSSYSSSSSSSSESYSISSEEYYYQPTPANFSYAPEAPFLPFFTGYKGYNIFYARNVDAIRSVGKLVEEIASDLENPSDLPKSNTMSKFNILTRAIRAMGYEDIYELAQKYFVSQKERQVAQFSDKKFSKRVDAWVTLRDAVAEAGTPSAFKLIFDFIKEKKLRGYEAATVIASLAQSIRYPTEHLLHEFFLLVTSDVVLHQEYLNATALFAYSNFVNQAHVSNRSAYNYYPVFSFGRLADADYKIIEHKIVPWFAHQLREAVNEGDSVKIQVYIRSLGNLGHPQILSVFEPYLEGTIQITDFQRLAIMVALDNLVIYYPSLARSVLYRAYQNTADVHEVRCAAVHLLMRTDPPADMLQRMAEFTHHDPSLYVRAAVKSAIETAALADDYDEDSKLAINAKAAINFLNPEDVSIQYSFNHIRDYALENLELSYRLHYGEIASNDHRYPSGLFYHLRQNFGGFKKYTSFYYLVSSMEAFFDIFKKQYNTKYFADYYKSADYSTNYYNFDKYSKYYKQYYYSKDSEYYQKFYGQKKDYYNDKEPFKFTAPRIAKLLNIDAEEAEQLEGQLLFKLFNGYFFTAFDNQTIENLPHKMRHLFENLEDGYAFDVTKFYQQQDVVLAWPLATGFPFIYTLKAPTVFKFEVDASAKTHPQVYKMPAGHPETENDDFFYMPQSINGSVDVNLLYHRMVDAKVGFVTPFDHQRYIAGYQKKLHGYLPFNVELGLDFVKDEYEFEFKFLEPKDDHLLFHMSSWPYTGYKDITDMRPIAENPNAKIVHDDNQSTKTMEHTFGQDMTGVALRFHAKYDFDLINFQQFWSLVQKNDFVSAVNYPFAYQPYEYHQFNLFYDSQRTHAKSFKFYAYQKFGAPSFEETGPKHPANRHSYSGNYYESNYAQPFVYSPGSQRRYEQFFRNAASGIRNSFVRYYDFGFEFYAPQYKSEFTFTTAFADSPVDKTSRQLYYFYASPMFPSQSYFKDIPFSGKQFQFCATATSEFPRVPYLKFSDFDKYYGDASQYFDFLYGESCQGGAHIAVKGKQKQTGKYREYLRFSDVAKACKEQMANGYYQFEECQQAIDQAYYYDFYDYAIEYKDVGSVAKNLTNKFYNYFQYAFYPYFESNFFYHGKSNYIKAEFEFAPYGDYYNASFFGPSYAFQVQNYPVFNDYSTYFPYFFKYTFFPRYQPYYMHRLPAHKPRNRPYYELSNYEQFAVFDRKPQYPSCSFSNDYFYTFDNKKYFYDMGECWHAVMYTVKPDYDFYAQQSHFYNSDFEYKYKNGFEEYEQFAALARRGSDNQLYFKFLFGDNYIEVFPNNGGIPFVKYNGRPYDISKSNIAHFEYKEGYPSFPFFYAFAYPNKDLEVSFFGGKLKFATDGYRARFFSDYSFYNNFVGLCGTNNGEYFDEFVTPDQCYMRKPEFFAASYAITGQNCTGPAKAFNYAYQQKAKQECVKREVYYGDIIYNQEYYHPRYRYYNHNVEESSSSSSSSSSDSSSSSSSSESSSRSRSGSSSSSSSSEEQKEFHPHKQEHSMKECPVQHQHQFFEQGDRTCFSLRPLPVCHSKCVATEKISKYFDVHCFEKDSTQAKKYKSDIGRGYTPDFKSFAPHKTYKFNYPKSCVYKAY

>AAQ92366.1 vitellogenin-C [Aedes aegypti]

MLVKLFLLALVGISTAYQYSYRSEFPYGRPDNKTGFEFGAWEPNRQYVYNVTSKTMTALPDLEDQWTGTFTRAYLVIRPKSPDYVFGYVKQPEYAVFNEYLPQGINTELSHRSLKWRPMPMSSKPIAIRYQKGTIKGFYVEQTVPNHEVNILKAWLSQFQLDTQGHHTYKSKYNQFPGNNSFTGVYKVMEPVVTGKCKTLYDVSVVPPYMIQANKQWVPQPQLREEGQYFFQVVKTQNFDHCQQRMGYHFGFSGYSDFRPNTNSMGNVASKSAVTNMYLTGTWYNYTIQSSSTVNKIAVAPSLINKQKAIVYAAVNCTLNRVEEYKQIPTGPAEDQKVFVDLVYSYNMPSDKKNNVRPTNATSSSSSSSSSSSSSSSSSSESSSSDSSSSESQENPKISPVYQYKAQLDEVEKRGNRNRRDLNAFKEKKYYEAYKLDQYRLSRKNDTSSDSSSSDDSSSSSSSSSSQESNERNNSSSSSSSSSSSSSSSQSYSSSSSSESYSLSSEEFYYQPAPESFKDAPQAPFLPFFTGYKGYNIQYARNVDGQRSIYRLVQEAVDELQNPSTLPKSNTLSKFNIVSRIFRTMNYQDIYEVAQKYFVSQQERKEGNNKSEKFAKRVDAWIFIRDALAEAGTPPAFKVIKEFIEEKKLRGYEAAGVFSTLVNSIRYPTESLLHEFFLLATSDTVQHQEGLNTTALFTYSYFVNQAHVNNRSAYNYYPVYSFGRFADADYKIVAHKIVPWFSHQLREAVNARDSVKAQVYIRCLGNLGHPEILNVFEPYLEGKYQVSDYQRLAMVVAFDKLVENYPHLARSILYKVYQNIGDIHQIRCAAVHMLMRANPPADMLQRMAEYTYYDPSRYVRAAVKSALESAAESYDYDYYNEFAENAKAAVKFLNPEDFSFQYSSSYIRDYAFENQEMSYRMYYGQIAADDHVMPNGMFFQLRNNFGGYKKYSSSYYLFSSMEAFFDLVDKQCDRSYFKDDYKSSDYYYKYYKQFPNKKSEYFDKYYKSHGPQSDYYQKFAKAEYNDKEPQKYSTTRIAKLLNIDPREAEELEGQFLVKLFNGYHFYAFNNQTIENSPQYIKKLFRELEDGLNFNYTKFYQQEEASLAFPLATGFPFVYTLKTPTVFKFETEAKVKTYPRVHQKPTGHPENENDDFIYWPKLLNGSIDVNLLYHRMSDAKVGFVTPFDHQRYIAGYQKKLQVNLPFSVDLSLNFETDEYEIELSPLEPKKDQLFFHLSSWPYTGYKDITEMRPIADSPNVRIVHDDDQTTKSFEHTFGQDMTGFGLRFHAKYDQDFFNFEQFYDFYKQHDFYSAFFYPFATQPYEYHQFNVYYDAQRTDIEKIKFSAHYKEGDFDQDFQDSDVKHPKGRHGYSGYYNEANYAQPIVFSAASHRRQDQFIKNAAAGIRNSDVGVFDFGVTFEGKKQKAEYVFTTAYADSPVDEKSRFLFFFSGSPYYQSNYFFGMPYQGKQFQMCFSATNEFPNVPKLNFLNALNQNEDADLKWELSYGEKCQGGAQVSIKGKLHQTDMYRHHLRTSRVGSTCKDQMDRGFYQLKECQNATRQASYFDQYFFKFDFKNFDSASQNLTYKFFNFFQQFAYPYYESNYFYKGKDNQAQFNFELAPYADCFNASFFGPEFAFKVENYPIYNYYYRYFATVHSDLSFFDRFATYAYRGQYHPSCSISSKYVATFDGKTYDYTMGDCWHVVLHTVKPDYEYYAYQSHFQNADSEYRFKNGFYEDEQISVLGRSGPSNEMYFKVILGQYKQNDYNIDIIPNGADLPKVYINGKPQQIHDKYAVELYTNDNGGEQPLFRCYALPGNELEINIRNDGLKIVHDGYRARFFADQSYYNNFAGLCGTNNGEYYDDFVTPDQCYMRKPEYFAASYAITGQNCTGPAKAFNYAYQQKAKEECVKREVFYGNVIYERDFYRQRYRYYNHNVEESDSSSSSSSSSSSDSSSSSSDSSSSSSSESRSRSHSGSSSSSSSEENKEYHPHQQQHSVKECEVKHQHQFFEQGSQICFSVRPLPACPSHCAATDKTPKYFDVHCRSASDPAAQLYKSQIQKGYSPDMKSRSVSKTVKFNIPKTCVHSQ

>AEO51020.1 vitellogenin 1 [Anopheles culicifacies]

MIAKLLLLTFVGLCTAYQYSYEYEFPSSRPFNKTGFEFGAWEPNREYVYNVTTKTMTALPDLEDYWTGIVTHGYLVIRPKDHNYVVAYIDRPMYAAFNEYLPRGYRTELSHFNLKWQPMPFSSKPFGIYYNKGAVKGFYVEKSVPNHEVNMLKGWVSQFQLDTQGAYVIKSEFNQFPENNTLTGVYKTMEPSVTGECETLYDVNPVPEYHFQSHKEWVPQPQWLEEDQHVFHVVKSRNFDRCEQRMGFHFGFSGFSDFKPNTNQMGNIMTKSEVTQMYLTGNWYNYTIQSVSTVNKVVVSPSLVNSQKAMSTLQVNMTLNEITPYNKYPEGPADDRQVFVDLVYSYNMAHDKKNFVRPANETDDSSSSSSDSSSDSSSSSDSSSSSEEEHENFKISPSEQYKKQVKEVEHRGNRNRRDLNAFKEKQYYESYKRDQYRLRKHNDTSSDSSSSDDSNSSSSSSSSDESDEHDFYSSSESDSHSLSSEEDFYQPIPESMKEAPQTPFLPYFTGYKGYSVQYAHNVDASRYAYKLAYEIAEELQEMSQVPKSNTLNKFTILARVLRTMHYQDIYDVCQKLFVSQKEREEGSNHSESFAKKCDAWNTFRDALAQAGTPPAFKVIKELIEEKKLRGDEAASVIATLPKTIRYPTETVMHEYFLLVTSNAVQHQEYLNTTALISFCDFLNRAQVNNRSAYNYYPVHSFGRLADADYKIVAHKVVPWFAHQLREAVKAGDSVKVQVYIRCLGHLGHPEILNVFEPYLEGKIPVTHFQRLAFIVALDRLVENYPRLARSVLFKVYQNTGDAHEVRCAAVYLLIRTKPPVYMLQRMAEQTHYDPSTYVRAAVKTALESASEADEFDDDYEFSQNAQAAIKHLNPRDFSLQYSGTYLRDFAFKELELSYRMYFSQIAADDHYVPSGFFFHLRKNMGGLKRFSTFYYLVSSMETFFDLLDKQYDSYNKHSEYKSSDYYYKYYKQYPSLFKDYFSQYSKNHKYQNDYYEQFGNKNQEEFQKWSTTRIAKLLNIDPEEAEELEGQFMFQIFNGERFFAFNNQTIEQFPSFVKKYFEDFEDGFAYNVTKFYQQNVVTMAFPLATGLPFTYSLKTPTLMKFEFEATATTHPSIYKTPTGYPEKEYDDFIHLPRWFNGSADVNMAYSRLVDAKVGFITPFDHQRYVAGYQKKFQGYLPFSFDFGFDFENNDFEVNVQPLEPKKDALLFHMSSWPYTGYKDITDLRPMAEQPSVHILHDRAQTTKSFETSFGHELTGVALRFQAKYDKDFIDYAYLMKHIEQHDYWSALVYPFASETYHYHQLNLYYDAQRTSVKNVKFVLQHKQADYDQDFQTADVKHPKGRHGFSGYYNEFNYAQPFVYYAGSQRRQEQFMRNAGAGIRNSDVNVFDFGIVFEGKQQKAEFVFTTAYADSPVDEKERLLMFLSFSPYVSSSAFYEFIPFSGKQFQMCFSATNQYPNMPKLNFLNVLNFDKIGSMDWELSYGEKCQGGSHVSMKGKLIQSEPYRHFLRISEAGQRCKQQMDKGYFQLPACQNATRQAGYFDQYSFNFEYKDVSNYAKNLTYQFFDYARYFSFPYWSEDYFFQGKHNQFQIDFQLAPYFDYYNASFYGTDRSFAIQNYPIESEYARYFFSIHPDFDYYERMFNYAYRGNYHPSCVVSNKFVNTFDGKTYDYELGNCWHVVLHTVKPDYYFYAQDSHFMNSDYEYNWKNGFGEDEQITILARHGEDNQLFLKAILGQYKQNDYNIDIIPHGHELPMVYINGKPQQIHEKYAVEMYTNDDGGDQPLIRVYALPGNELEISFRDDDIKIVFDGYRARFFADQSYFNNFVGLCGTNNGEGEDDFITPDQCVMRKPEYFAASYALSGMNCSGPAQAYFTEYHQKAQEHCVKPQYYFGNVISEQEAGRQRYNYYYKDFDLSDSSSSESSSSSSSSESDESDDSNSSSSEEQKPNREHFFEKQQYTEKECPVKHQAQYVEQGDKICFTSRPLPACASQCKATEKVPKYVDVHCRDVTDSVAQLYKQQIRKGVNPDMSNKSVTKTVKFFLPKKCVHVY

>XP_313104.2 AGAP004203-PA [Anopheles gambiae str. PEST]

MIAKLLLLTLVGLCTAYQYSYEYEFPSSRPFNKTGFEFGAWEPNKEYVYNVTTKTMTALPDLEDYWTGIVTHGYLVIRPKDHNYVVAYIDRPMYAAFNEYLPRGYRTELSRFNLKWQPMPFSSKPFGIYYNKGAVKGFYVEKTVPNHEVNMLKGWVSQLQLDTQGAYVIKSEFNQFPENNTLTGVYKTMEPSVTGECETLYDVNPVPEFHFQSHKEWVPQPQWLEEDQHVFHVVKSRNFDHCEQRMGFHFGFSGFSDFKPNTNQMGNIMTKSEVTQMYLTGNWYNYTIQSVSTVNKVVVSPSLVNSQKAMVYAQVNMTLNEITPYDKYPEGPADDRQVFVDLVYSYNMAHDKNNFVRPANETDDSSSSSSSSSSDSDSDSSSSSDSSSSSSEEEAENFKISPAEQYKKQAKEVERRGNRNRRDLNAFKEKQYYEAYKRDQYRLRKQNDTSSDSSSSDDSSSSSSSSSSSESDEHDFYSSSESDSDSLSSEEFYQPIPESMKDAPQTPFLPYFTGYKGYSVQYAHNVDASRYAYKLAYEIADELQEISQVPKSNTLNKFTILARVLRTMHYQDIYDVCQKLFVSQKEREEGSNHSESFAKKVDAWNTFRDALAQAGTPPAFKVIKELIEEKKLRGDEAASVIATLPKTIRYPTETVMHEYFLLVTSNAVQHQEYLNTTALISYCDFLNRAQVNNRSAYNYYPVYSFGRLADADYKIVAHKVVPWFAHQLREAVKAGDSVKVQVYIRCLGHLGHPEILNVFEPYLEGKIPVTHFQRLAFIVALDRLVENYPRLARSVLFKVYQNTGDAHEVRCAAVYLLIRTKPPVYMLQRMAEQTHYDPSTYVRAAVKTALESASEADEFDDDYEFSQNAQAAVKHLNPRDFSLQYSGTFLRDFAFKELELSYRMYFSQIAADDHYVPSGFFFHLRKNMGGLKRFSTFYYLISSMETFFDLLDKQYDSYNKHQEYKSSDYYYKYYKQYPHLFKDYFSQYNKNHKYQNDYYEQFGNKNQEEFQKWSTTRIAKLLNIDPEEAEELEGQFMFQIFNGERFFAFNNQTIEQFPSLVKKYFEDFEDGFAYNMTKFYQQNVVTMAFPLATGLPFHYSLKTPTLMKFEFEASATTYPSIFKTPTGYPEKENDDFIHMPRWFNGSADVNMAYSRLVDAKVGFITPFDHQRYVAGYQKKFQGYLPFSFDFGFDFENNDFEVNVQPLEPKKDVLLFHMSSWPYTGYKDIADLRPMAEQPSVHILHDRAQTTKSFEQSFGQSLTGVALRFQAKYDKDFIDYAYLMKHIEQHDYWSALVYPFASETYHYHQFNLYYDAQRTSVKNVKFVLQHKQADYDQDFQTADVKHPKSRHGFSGFYNEYNYAQPFVYYAGSQRRQEQFMRNAGAGIRNSDVNVYDFGIVFEGKQQKAEFVFTTAYADSPVDEKERLLMFLSFSPYVSSSAFFEFIPFSGKQFQMCFSATNQYPNMPKLNFLNVLNFDKVGSMNWELAYGEKCQGGSHVSMKGKLIQSEPYRHFLRISEAGQSCKQQMDQGYFQLPACQNATRQAGYFDQYSFNFEYKDVSNYAKNLTYQFFDYARYFTFPYWNEDYFFQGKHNQFQIDFQLAPYFDYYNASFYGSDRSFAIQNYPIESEYARYFFSVHPDFDYYERMFNYAYRGNYHPSCAVSNKFVNTFDGKTYDYELGNCWHVVLHTVKPDYYFYAQDSHFMNSDYEYNWKNGFGEDEQITILARHGEDNQLFLKAILGQYKQNDYNIDIIPHGHELPMVYINGKPQQIHEKYAVEMYTNDDGGDQPLIRVYALPGNELEISFRDDDIKIVFDGYRARFFADQSYFNNFVGLCGTNNGEGEDDFITPDQCVMRKPEYFAASYALTGMNCSGPAQAYFTEYHQKAQQHCVKPQYYFGNVISEQEAGRQRYNYYYKDFDLSDSSSSESSSSSDESDDSNSSSSEERKPNREHFFEKQQYTEKECAMQHRVQYIEQGDKICFTNRALPTCSSQCKAFEKIQKYVDVHCRDATDSAAQLFKQQIRKGVNPDMSNKSVTKTVKYFFPKKCVFAN

>XP_001843135.1 vitellogenin-A1 [Culex quinquefasciatus]

MMWWKFVLIVLVGTCFAEQQQFKRPQSYDEAAQRYEKLMDGGAKHYLHDNLAWEPNQVYWYNVTTKTMTALPDVTEQWTGVLTRAKMVINPKSDGYVVGRIDRAHYAQFNQYLADGHRSELSDLKLTWKPMPLSSKPFGIKYKKGAIKGLYVEKTVPNHEVNILKSWVSQLQLDLFGANLIKSKYNQLPENETANAVYKTMEPSVSGECETLYDVNVLPKYKIQSHDEWVPQPQYMQQDDEIFEIVKAKNFSNCDRRMGYHFGITGASDIKPNTNQMGNILSKSAVSQIFVVGKIHNHTIQSASTTSKILVSPSLVHTEKAMVLAQVNVTLNQIKRSQKEPVGPAEDRAVFVGLVYSYNLPEDKNNWARPENETSSSSSSSSSSSSSSSSDSSSSSDSSSSSSSSSSSSSESSSSSSSSSSEEKDNKKISPAEQHKDALKQVEKRERSTRNRRDLNAQKEKKYYEAYKMDQYRLSRDNDTSSDSSSSDDSSSSSSSSSSSESREHRKNGTLADNSSSSSSSSSSSSSSSDSKSSSSSSSSSSSESLDSSEEYYQPSPKDLDNAPAAPLLPFATGYKGSSIQNARNVDAPRIVAQLVKTIAEDFQNPSIIPKSNTLAQFNHLTRLIRTMDHQELYDCAQKLFVSEKERQQGDKHSEKFAIRCDAWNVFRDAMAEAGTPPVFKVIKQYIEEKKLRGIEAASVVATLPKRIRYPTETLMHEFFLLATSTAVQHQETLNATALIAFSDFLNRAHVNNQSALNYYPVNSFGRLADSKYKIVAHKAVPWLAHQLREAVQEADSERIQVYIRAIGNLGHPEILNVFEPYLEGKIPVTNFQRFAIVMSLDRLVENFPKLARTVLYRVYQNNADVDEVRCAAAMLLMRTSPPVAMLQRMAEKTDENNSPQVSALVKSLIESAANTDEFDDDSELAQNARAAVKMLNPNEYGLQYSSAHFRQYAMKELDISYRLQAGQIASDNHPVPTGAWLHWHENLGGLKRLSSYHYIVSNMDALFDLLDNKVMTVEEQKKEWRQESRQSRANEKQEKASKKQDNKAEQKWSTGRIAKLLNIDPEDVEQVEGQLWLEIFNAPHLIAFDNNTIDELPRVIKKFMKDLEVNWSVNITKVYQQGLVTVAMPLETGFPFTFSTQSPTLVKLEVDASAETMPNMARKPAGHPENGNDEHIHIPLSANVTADVNVVYSRLIDAKVGFNTPFDHQRYIAGFQKKHHIHAPLRLEAQLDNAQNEYELNIQPLEPKKDILLAHISSWPYTAYKDITDIRPIAESPNAQILHDAVRRTKTVEGTIGQQLTGVALRYQAKYDKPALVFGDIVEHIQQHDLMSALLFPLHASQPCHYHQLNLWYDAQRSPVKNIKLSLQQTTANETEDFSSSDIKHPKARHQSEGYYNEKNLAQPFVFKPASQRRQEQFLKNAGAGIRNSLVSVWDLGAEFEGRQNKAEFVLTFAKASSPVDEKERTLVFASASPYIAVGSKKQHQACLSLTEKYPSVPMLNYITALQNDVTSEIDLELSFGEKCAGGAQVSVNGMLRQTDLWRETLRSSAIGRKCKNQMAEGYFALPECQNATRLASALDHYTFDIEFKEIPSSVRNMTNKALNWVQSAVITRWEEDCVSHKGKEGKAQLKIELSPRVSHINVTLATPNRKIEIENLPVENEWMKSLVLVHPDLAWNERLASYAYNGEMNPSCVVAPKYVDTFDGRTYDYETGTCWHVAMHTVKPELEVSPEHSHFYASDVEQRWSNGFDEHEQITVLTRTVENNQQHLKVVLGQQEQWDYNIDIVPNGAQLPIVYVNDEPLQVHDKYTIPMYTADEGEQPLVRVHALAGKELVVDIRDGQIVIVCDGYRAQILTGQTFYDNTVGLCGTNNKQEEDDFITPQQCVMRKPEYFAASWAVTGQNCTGPAKAFAIASQQKQKEACLKVEYMYGNVVSDVEAGRKRYRYYNHNVDSSSSSESDSSSSSSSSESSESNSKSDSSSSSSSSGSSSSSSSESKEHDPSQQKYSMKECDMVHQVQYVEKDNELCFSKRPLPVCNSRCKAVEKEAKYVDVHCRSVQDSAAQMLKQQIRKGVNPDMSAKSVSKTMKFAVPKQCVHIH

>ADH04224.1 vitellogenin 1a [Culex tarsalis]

MFAKLLLLALVGISAAYQYEYQNEFYNQRPENKTGFEFGAWEPNREYVYNVTSRTMTALADLADQWTGVITRARLIVRPKDPDYVVCYVKQAEYANFNEELPQGYRTNIWRELGSKLKWQPMPFSSKPFAIRYRKGAIKGLYVEQTVPNHEVNILKAWASQLQLDTRGANVVKSKYNQFPENTTFTGVYKVMEPLVTGECETLYDVNVVPEHVIKGNKYFVPRPDLREENQYFVEVHKTQNFNRCEQRMGYHFGFSGHSDFKPNSNQMGNVASKAVVGHMFLTGTWYNYTIQSSSTTNKVVVAPSLVDDQKAMVYSQVNMTLDKVTPYEKIPMGPAEDRQVFVDLVYSYNMANDKKNNVRPGNETDSSSSSSSSSSSSSSSSSSSSSSESDSLSSSDSSSSSSEEQNNQKDNFKISPAEQHKKQLKEVKAHRPRRDLNAQKEKKYYEAYKMDQYRLSRNNDTSSDSSSSDDSKSSSSSSSSSESNEKNARISRFTNSPSSSSSSSSSSSSSSQSDSSSSSSESNSLSSEELYYQPMPESFKDAPQAPFLPFYTGYKGYTVQYARNVDGPRAVYKLVQEIADELQNPSSLPQSNTLNKFNILCRAIRTMDYKEIYEVAQKYFVSQKERKEGDNNSEKFSKRVDAWNTFRDALAEAGTPPAFKVIKEFIEEKKLRGAEAASVIATLPQSIRYPTEPLMHEFFLLVTSDTVQHQDSLNVTALISFTQFVNQAQVNNRSAYNYYPVHSFGRLADADYKIVAHKVVPWLAHQLREAVNEGDSIKVQVFIRALGNLGHPEILNVFEPYLEGKIPVSDFQRLCIVAGMDKLVENFPKLARSVLFKVFQNTGDVYEVRCAAVHLLIRAEPPVEMMQRMAQQTNEEPSLYVRAAVKTALEYAAQADEYEADSSFANNAKAAIKLLDPEVYGLQYSSNYLRDVALNNLEMAFRMYVGQIASDDHWLPNGMIWHLRKNLGGVKRHTTFWYLVSSMETFLDLVDKQYDSSSISEDYKSADYYYKYYQQFPDKKSEYFEKYYKAHESQNEMNKKYNEQNGGKYFQKYSTTRIAKLLNIDPQEAEELEGQLFFPLFNGKYFAAFNNQTIENIPRKLKELADDLEDGMSFNVTKFYQYQPLTLAFPLASGLPFVYTLKNPTVVKFETEATIKTHPSIFKKPAGHPETENDDFIHVPRVLNGSVDINLVFHRLIDSKIGFITPFDHQRYISGVQKKVQAFLPLSLELNWDLENDQYETELEILDKKEDKLLFHLSAWPYTGFKDITDMRPIAENPNAKIIHANEHNTQTFQQTVGQDMFGFALRVHAKYDQDIIDVQSILEHVQEGDYLAALFHPFAYQPLYYHQLNVYYDAQRTGAKKARFSAHYNEGDFDQDFQESDIKQPKGRHSYAGYFNENNLAQPFVFTAGSQRRQEQFIKNAAAGIRNSDVAVFDFGFVFEGRERKAEYVLTTAYADSPVDEKSRMLFFFSGSPFNPSERFFGVPHNGKQFQMCFSATNEFPNMPKLNFLNALNHDMDSALRWELSYGEKCQGGAQVSMKGKLKQSDEYRHKLRISEVGQRCKQQMDQGRFQLPECQNATRQAGYLDQYKFEVDFKDVGSWAKNWTHKAVDWVQHMTYPWFEPNYLFKGKNNKVEFEFEMSPYGDYFNVSAWAPEYAFEIENYPVDSFWARYFASAHTDLAWYERLGTYAYQGNYKSSCAVSSKYVDTFDGRNYQYNMGECWHVVLHTVKPDYEYYAQQSHFQNADTEYRFKNGFSEDEQISVLARSGPSQELYFKVVLGQYKQNDYNIDVLPKGADLPEVFINGKPQQIHEKYAVELYTNDNGGDQPLFRCYALPGKELEISIRNDDVKIVFDGQRARIFANQKYFDNFVGLCGTNNGELYDDFVTPDQCFMSKPEFFAASYALTGQNCSGPAKAFNVFYQQKVKEECVKEEVFYGNVISEQEAGRKRYRYYNHNVEDSSSSSDSSDSSSSSSSSSSSSESDSSSDSSSNSSSSSSEEEKRTRDINKESKDTYKEFHADRQKHTLKECDIQHQHQIVEQGDKTCITKRPLPVCPSHCVASNKLQKYVDVHCNNSDDQSVKLYKNQISKGYNPDMSSHPVTKTIKFNIPKTCVFAQ

>ADH04225.1 vitellogenin 1b [Culex tarsalis]

MFAKLLLLALVGISAAYQYEYQNEFYNQRPENKTGFEFGAWEPNREYVYNVTSRTMTALADLADQWTGVITRARLIVRPKDPDYVVCYVKQAEYANFNEELPQGYRTNIWRELGSKLKWQPMPFSSKPFAIRYRKGAIKGLYVEQTVPNHEVNILKAWASQLQLDTRGANVVKSKYNQFPENTTFTGVYKVMEPLVTGECETLYDVNVVPEHVIKGNKYFVPRPDLREENQYFVEVHKTQNFNRCEQRMGYHFGFSGHSDFKPNSNQMGNVASKAVVGHMFLTGTWYNYTIQSSSTTNKVVVAPSLVDDQKAMVYSQVNMTLDKVTPYEKIPMGPAEDRQVFVDLVYSYNMANDKKNSVRPGNETDSSSSSSSSSSSSSSSSSSSSSSSSESDSSSSSDSSSSSSEEQNNQKDNFKISPAEQHKKQLKEVKARRTRRDLNAQKEKKYYEAYKMDQYRLSRNNDTSSDSSSSDDSKSSSSSSSSSESNEKNARISRFTNSPSSSSSSSSSSSSSSQSDSSSSSSESNSLSSEELYYQPMPESFKDAPQAPFLPFYTGYKGYTVQYARNVDGPRAVSKLVQEIADELQNPSSLPQSNTLNKFNILCRAIRTMDYKEIYEVAQKYFVSQKERKEGDNNSEEFSKRVDAWNTFRDALAEAGTPPAFKVIKEFIEEKKLRGAEAASVIATLPQSIRYPTEPLMHEFFLLVTSDTVQHQDSLNVTALISFTQFVNQAQVNNRSAYNYYPVHSFGRLADADYKIVAHKVVPWLAHQLREAVNEGDSIKVQVYIRALGNLGHPEILNVFEPYLEGKIPVSDFQRLCIVAGMDKLVENFPKLARSVLFKVFQNTGDVHEVRCAAVHLLIRAEPPVEMMQRMAQQTNEEPSLYVRAAVKTALEYAAQADEYEADSSFANNAKAAIKLLDPEVYGLQYSSNYLRDVALNNLEMAFRMYVGQIASDDHWLPNGMIWHLRKNLGGVKRHTTFWYLVSSMETFLDLVDKQYDSSSISEDYKSADYYYKYYQQFPDKKSEYFEKYYKAHESQNEMNKKYNEQNGGKYFQKYSTTRIAKLLNIDPQEAEELEGQLFFPLFNGKYFAAFNNQTIENIPRKLKELADDLEDGMSFNVTKFYQYQPLTLAFPLASGLPFVYTLKNPTVVKFETEATTKTHPSIVKKPAGHPETENDDFIHVPRVLNGSVNVNLVFHRLIDSKIGFITPFDHQRYISGVQKKVQAFLPLSLELNWDLENDQYETELEILDKKEDKLLFHLSAWPYTGFKDITDMRPIAENPNAKIIHANEHNTQTFQQTVGQDMFGFALRVHAKYDQDIIDVQSILEHVQEGDYLAALFHPFAYQPLYYHQLNVYYDAQRTGAKKARFSAHYNEGDFDQDFQESDIKQPKGRHSYAGYFNENNLAQPFVFTAGSQRRQEQFIKNAAAGIRNSDVAVFDFGFVFEGRERKAEYVLTTAYADSPVDEKSRMLFFFSGSPFNPSERFFGVPHNGKQFQMCFSATNEFPNMPKLNFLNALNHDMDSALRWELSYGEKCQGGAQVSMKGKLKQSDEYRHKLRISEVGQRCKQQMDQGRFQLPECQNATRQAGYLDQYKFEVDFKDVGSWAKNWTHKAVDWVQHMTYPWFEPNYLFKGKNNKVEFEFEMSPYGDYFNVSAWAPEYALEIENYPVDSFRARYFAAAHTDLAWYERLGTYAYQGNYKSSCAVSSKYVDTFDGRNYQYNMGECWHVVLHTVKPDYEYYAQQSHFQNADTEYRFKNGFSEDEQISVLARSGPSQELYFKVVLGQYKQNDYNIDVLPKGAELPEVFINGKPQQIHEKYAVELYTNDNGGDQPLFRCYALPGKELEISIRDDDVKIVFDGQRARIFADQKYFDNFVGLCGTNNGELYDDFVTPDQCFMSKPEFFAASYALTGQNCSGPAKAFNVFYQQKVKEECVKEEVFYGNVISEQEAGRKRYRYYNHNVEDSSSSSDSSDSSSSSSSSSSSSESDSSSDSSSNSSSSSSEEEKRTRDINKESKDTYKEFHADRQKHTLKECEIQHQHQIVEQGDKTCITKRPLPVCPSHCVASNKLQKYVDVHCNNSDDQSVKLYKNQISKGYNPDMSSHPVTKTIKFNIPKTCVFAQ

>ADH04226.1 vitellogenin 2a [Culex tarsalis]

MWWKFVLIVLVGTCFAEQQQRPQNYDEAVKRYEKLMDGGAKHYLRDNLAWEPNQVYLYNVTTKTMTALPDSTEQWTGVLTRARMIINPKSDGYVVGRIENAQYTQFNQYLEDGHRTDFSDLKLTWRPMPLASKPFGIKYKKGAIKGMYVEKTIPNHEVNILKSWVSQLQLDLFGANLIKSKYNQLPENETANAVFKTMEPSVSGECETLYDVNVLPKYKIQSHDEWVPRPQYMQQDDEIFEIVKAKNFSNCERRMGYHFGITGASDIKPNTNQMGNILSKSAISQIFVSGKIHNHTIQSASTTSKILVSPSLVHTEKAMVLAQVNVTLNQIKRSQKEPTGPAEGRAVFVGLVYSYNLPEDKNNWARPENETSSSSSSSSSSSSSSSSSSDSSSSSSSSSSSSSESSSSSSSSEEKDNKKISPAEQYKDSLKQVEKNERTNRNRRDLNAQKEKKYYEAYKMDQYRLSRNNDNSSDSSSSDDSSSSSSSSSLESREQRQNGTLADNSSSSSSSSSSSDSKSSSSSSSSSSSSSSSESLDSSEEYYQPAPTGLDNAPAVPLLPYATGYKGSSIQNARNVDAPRIVAQLVKDIAEGFQNPSTIPKSNTLSKFNHLARLLRTMDHQELYDCAQKLFVSEKERQQGDKQSEKFAIRVDAWNVFRDAMAEAGTPPVFKVIKQYIEEKKLRGIEAASVIATLPQRIRYPTETLMHEFFLLATSSAVQRQETLNATALIAFSDFLNRAQVNNQSALNYYPVNSFGRLADSKYKIVAHKAVPWLAHQLREAVQDADSERIQVYIRAIGNLGHPEILNVFEPYLEGKLPVTNFQRFAIVMSLDRLVENFPKLARTVLYRVYQNNADVDEVRCAAAMLLMRTSPPVAMLQRMAEKTDENNSPQVSALVKSLIESAANTEQFDDDSELAQNARAAVKMLNPNEYGLQYSSAHFRQYAMKELDMSYRLQAGQFASDNHPVPTGAWVHWHENLGGLKRLSSYHYIVSNMDALFDLLDKKVMTVEEQQKEWRQESRQSRANEKQEKAWKKQDNKAEQKWSTGRIAKLLNIDPEDVEQVEGQLMLEIFNAPYLIAFDNNTIGELPRVIKKWMKDLEVNWGVNITKVYQQGLLTVAMPLETGFPFTFTTQSPTLVKFEVDASAQTIPNMAKKPAGHPENGNDEHIHIPLSANISADVNVVYSRLIDAKVGFNTPFDHQRYIAGFQKKHHVQAPLRLEAQLNNAANEYELNIQPLEPKKDILLAHISSWPYTAYKDVTDIRPIADSPNAKILHDAVRRTKSIEGTIGQQLTGVALRYQAKYDKPALVFGDIAEHIQQHDWVSALLFPLHASQPCHYHQLNLWYDAQRSPVKNIKLSLQQTTANESEDFSSSDIKHPKARHQSEGYYNEKNLAQPFVFKPASQRRQEQFLKNAGAGIRNSLISVWDLGAEFEGRQNKAEFVLTLAKASSPVDEKERTLMFASASPYIAVGSKKRYQACLSLTEKYPSVPMLNYFTALQNDVSSQIDLELSFGEKCAGGAQVSINGKLYQTDLWRDNLRSSEIGKKCKNQMAEGYYALPECQNATRLASALDQYTFDIEFKEIPSSVRNMTNKALNWVQSAVITRWEEDHVSHKGKEGKAQLKIELSPRVSHINVTLATPNRKIEIENLPVENEWVKNLVQVHPDLDWNERLASYAYNGEMNPSCVVAPKYVNTFDGRTYDYETGTCWHVAMHTVKPELEVSPDQSHFYAADMDQRASNGFDENEQITVLTRTVENNQQQLKVVLGQQGQWDYNIDIVPNGAQLPIVYVNDEPLQVHDKYTIPMYTSDEGEQPLVRVHALAGKELVVDIRDGQVVIVCDGYRAQILTGQVFYDNTVGLCGTNNKQEEDDFITPQQCVMRKPEYFAASWAVTGQNCTGPAKAFAIASQQKQNEACLKVEYLYGNVVSDADAGRKRYRFYNHNVDSSSSSESDSSSSSSSSDSSESNNKSDSSSSSSSSESKEYNPAQQKYSGKECDITHQVQYVERGSEICFSKRPLPVCNSNCKAIEKDTKYVDVHCRQAQESAAQMLKQQIRKGVNPDMSAKSVSKTVKFAVPKKCVHVL

>ADH04227.1 vitellogenin 2b [Culex tarsalis]

MWWKFVLIVLVGTCFAEQQQQQQRPQNYDEAAKRYEKLMDGGAKHYLHDNLAWEPNQVYLYNVTTKTMTALPDITEQWTGVLTRAKMIINPKSDGYVVGRIENAQYTQFNQYLEDGHRTDFSDLKLTWRPMPLASKPFGIKYKKGAIKGLYVEKTIPNHEVNILKSWVSQLQLDLFGANLIKSKYNQLPENETANAVFKTMEPSVSGECETLYDVNVLPKYKIQSHDEWVPRPQYMQQDDEIFEIVKAKNFSNCERRMGYHFGITGASDIKPNTNQMGNILSKSAISQIFVSGKIHNHTIQSASTTSKILVSPSLVHTEKAMVLAQVNVTLNQIKRSQKEPTGPAEDRAVFVGLVYSYNLPEDKNNWARPENETSSSSSSSSSSSSSSSSSSDSSSSSDSSSSSSSSSSSSSESSSSSSSSEEKDNKKISPAEQYKDSLKQVEKNERTNRNRRDLNAQKEKKYYEAYKMDQYRLSRNNDTSSDSSSSDDSSSSSSSSSSESREQRQNGTLADNSSSSSSSSSSSDSKSSSSSSSSSSSESLDSSEEYYQPAPTGLDNAPAAPLLPYATGYKGSSIQNARNVDAPRIVAQLVKDIAEDFQNPSTIPKSNALSKFNHLARLLRTMDHQELYDCAQKLFVSEKERQQGDKQSEKFAIRVDAWNVFRDAMAEAGTPPVFKVIKQYIEEKKLRGIEAASVIATLPQRIRYPTETLMHEFFLLATSSAVQRQETLNATALIAFSDFLNRAQVNNQSALNYYPVNSFGRLADSKYKIVAHKAVPWLAHQLREAVQEADSERIQVYIRAIGNLGHPEILNVFEPYLEGKLPVTNFQRFAIVMSLDRLVENFPKLARTVLYRVYQNNADVDEVRCAAAMLLMRTSPPVAMLQRMAEKTDENNSPQVSALVKSLIESAANTEQFDDDSELAQNARAAVKMLNPNEYGLQYSSAHFRQYAMKELDMSYRLQAGQFASDNHPVPTGAWVHWHENLGGLKRLSSYHYIVSNMDALFDLLDNKVMTLEEQQKEWHQESRQSRANEKQEKAWKKQDNKAEQKWSTGRIAKLLNIDPEDVEQVEGQLMLEIFNAPYLIAFDNNTIEELPRVIKKWMKDLEVNWSVNITKVYQQGLLTVAMPLETGFPFTFTTQSPTLVKFEVDASAQTMPNMAKKPAGHPENGNDEHIHIPLSANISADVNVVYSRLIDAKVGFNTPFDHQRYIAGFQKKHHVQAPLRLEAQLNSAANEYELNIQPLEPKKDILLAHISSWPYTAYKDITDIRPIADSPNAKILHDAVRRTKSIEGTIGQQLTGVALRYQAKYDKPALVFGDIAEHIQQHDWVSALLFPLHASQPCHYHQLNLWYDAQRSPVKNIKLSLQQTTANESEDFSSSDIKHPKARHQSEGYYNEKNLAQPFVFKPASQRRLEQFLKNAGAGIRNSLISVWDLGAEFEGRQNKAELVLTLAKASSPVDEKERTLMFASASPYIAVGSKKQYQACLSLTEKYPSVPMLNYFTALQNDVSSQIDLELSFGEKCAGGAQVSINGKLYQTDLWRDNLRSSEIGKKCKNQMAEGYYALPECQNATRLASALDQYTFDIEFKEIPSSVRNMTNKALNWVQSAVITRWEEDHVSHKGKEGKAQLKIELSPRVSHINVTLTTPNRKIEIENLPVENEWVKNLVQVHPDLDWNERLASYAYNGEMNPSCVVAPKYVDTFDGRTYDYETGTCWHVAMHTVKPELEVSPDQSHFYAADMDQRASNGFDENEQITVLTRTVENNQQQLKVVLGQQGQWDYNIDIVPNGAQLPIVYVNDEPLQVHDKYTIPMYTSDEGEQPLVRVHALAGKELVVDIRDGQVVIVCDGYRAQILTGQVFYDNTVGLCGTNNKQEEDDFITPQQCVMRKPEYFAASWAVTGQNCTGPAKAFAIASQQKQNEACLKVEYLYGNVVSDADAGRKRYRFYNHNVDSSSSSESDSSSSSSSSDSSESNNKSDSSSSSSSSESKEYNPAQQKYSGKECDITHQVQYVERGSEICFSKRPLPVCNSNCKAIDVVNKYYDFHCHPLEDTTAQLWKEQIRKGVNPDLSVKAVSKTIKMTAPKKCVHVK

Supplementary Table 3. Concentrations of dsRNAs targeting *AhVg1*, *AhVg2*, and *AhVg3* before concentration adjustment.

| **Gene** **name** | **ds*EGFP*** | | **ds*Vg1*** | | **ds*Vg2*** | | **ds*Vg3*** | |
| --- | --- | --- | --- | --- | --- | --- | --- | --- |
|  | **A** | **B** | **A** | **B** | **A** | **B** | **A** | **B** |
| **Concentration (ng/ul)** | **14500** | **13210** | **12500** | **12200** | **11460** | **11150** | **16900** | **147400** |
| **Concentration after**  **Controlled (ng/ul)** | **10000** | **10000** | **10000** | **10000** | **10000** | **10000** | **10000** | **10000** |

Supplementary Table 4. Amount of ds*Vg1*, ds*Vg2*, and ds*Vg3* injected in each group.

| **Gene name of** **RNAi** | **ds*EGFP* (ug)** | | **ds*Vg1* (ug)** | | **ds*Vg2* (ug)** | | **ds*Vg3* (ug)** | |
| --- | --- | --- | --- | --- | --- | --- | --- | --- |
|  | **A** | **B** | **A** | **B** | **A** | **B** | **A** | **B** |
| **d*sVg1*** | **1** | **1** | **1** | **1** |  |  |  |  |
| **ds*Vg2*** | **1** | **1** |  |  | **1** | **1** |  |  |
| **ds*Vg3*** | **1** | **1** |  |  |  |  | **1** | **1** |
| **ds*Vg1*&*2*** | **2** | **2** | **1** | **1** | **1** | **1** |  |  |
| **ds*Vg1*&*3*** | **2** | **2** | **1** | **1** |  |  | **1** | **1** |
| **ds*Vg2*&*3*** | **2** | **2** |  |  | **1** | **1** | **1** | **1** |
| **ds*Vg1*&*2*&*3*** | **3** | **3** | **1** | **1** | **1** | **1** | **1** | **1** |
